# Genetic associations of protein-coding variants in human disease

**DOI:** 10.1038/s41586-022-04394-w

**Published:** 2022-02-23

**Authors:** Benjamin B. Sun, Mitja I. Kurki, Christopher N. Foley, Asma Mechakra, Chia-Yen Chen, Eric Marshall, Jemma B. Wilk, Biogen Biobank Team, Benjamin B. Sun, Chia-Yen Ghen, Eric Marshall, Jemma B. Wilk, Heiko Runz, Mohamed Chahine, Philippe Chevalier, Georges Christé, FinnGen, Mitja I. Kurki, Aarno Palotie, Mark J. Daly, Aarno Palotie, Mark J. Daly, Heiko Runz

**Affiliations:** 1 Translational Biology, Research and Development, Biogen Inc., Cambridge, MA USA; 2 BHF Cardiovascular Epidemiology Unit, Department of Public Health and Primary Care, University of Cambridge, Cambridge, UK; 3 Psychiatric and Neurodevelopmental Genetics Unit, Massachusetts General Hospital, Boston, MA USA; 4 The Stanley Center for Psychiatric Research, The Broad Institute of MIT and Harvard, Cambridge, MA USA; 5 Institute for Molecular Medicine Finland (FIMM), University of Helsinki, Helsinki, Finland; 6 Analytic and Translational Genetics Unit, Department of Medicine, Massachusetts General Hospital, Boston, MA USA; 7 MRC Biostatistics Unit, School of Clinical Medicine, University of Cambridge, Cambridge, UK; 8 Optima Partners, Edinburgh, UK; 9 Université de Lyon 1, Université Lyon 1, INSERM, CNRS, INMG, Lyon, France; 10 CERVO Brain Research Center and Department of Medicine, Faculty of Medicine, Université Laval, Quebec City, Quebec Canada

**Keywords:** Genetic association study, Genetic variation, Diseases, Genetic predisposition to disease

## Abstract

Genome-wide association studies (GWAS) have identified thousands of genetic variants linked to the risk of human disease. However, GWAS have so far remained largely underpowered in relation to identifying associations in the rare and low-frequency allelic spectrum and have lacked the resolution to trace causal mechanisms to underlying genes^1^. Here we combined whole-exome sequencing in 392,814 UK Biobank participants with imputed genotypes from 260,405 FinnGen participants (653,219 total individuals) to conduct association meta-analyses for 744 disease endpoints across the protein-coding allelic frequency spectrum, bridging the gap between common and rare variant studies. We identified 975 associations, with more than one-third being previously unreported. We demonstrate population-level relevance for mutations previously ascribed to causing single-gene disorders, map GWAS associations to likely causal genes, explain disease mechanisms, and systematically relate disease associations to levels of 117 biomarkers and clinical-stage drug targets. Combining sequencing and genotyping in two population biobanks enabled us to benefit from increased power to detect and explain disease associations, validate findings through replication and propose medical actionability for rare genetic variants. Our study provides a compendium of protein-coding variant associations for future insights into disease biology and drug discovery.

## Abstract (web summary)

A meta-analysis combining whole-exome sequencing data from UK Biobank participants and imputed genotypes from FinnGen participants enables identification of genetic associations with human disease in the rare and low-frequency allelic spectrum

## Main

Inherited variations in protein-coding and non-coding DNA have a role in the risk, onset and progression of human disease. Traditionally, geneticists have dichotomized diseases as either caused by coding mutations in single genes that tend to be rare, highly penetrant and frequently compromise survival and reproduction (often termed ‘Mendelian’ diseases), or as common diseases that show a complex pattern of inheritance influenced by the joint contributions of hundreds of low-impact, typically non-coding genetic variants (often termed ‘complex’ diseases). For both rare and common conditions, large human cohorts systematically characterized for a respective trait of interest have enabled the identification of thousands of disease-relevant variants through either sequencing-based approaches or GWAS. Nevertheless, the exact causal alleles and mechanisms that underlie associations of genetic variants to disease have so far remained largely elusive^1^.

In recent years, population biobanks have been added to the toolkit for disease gene discovery. Biobanks provide the opportunity to simultaneously investigate multiple traits and diseases at once and uncover relationships between previously unconnected phenotypes. For instance, the UK Biobank (UKB) is a resource that captures detailed phenotype information matched to genetic data for more than 500,000 individuals and, since its inception, has facilitated biomedical discoveries at an unprecedented scale^2^. We and others have recently reported on the ongoing efforts to sequence the exomes of all UKB participants and link genetic findings to a broad range of phenotypes^3–6^. We also established FinnGen (FG) (https://www.finngen.fi), an academic–industry collaboration to identify genotype–phenotype correlations in the Finnish founder population with the aim of better understanding how the genome affects health. Finland is a well-established genetic isolate and a unique gene pool distinguishes Finns from other Europeans^7^. The distinct Finnish haplotype structure is characterized by large blocks of co-inherited DNA in linkage disequilibrium and an enrichment for alleles that are rare in other populations, but can still be confidently imputed from genotyping data even in the rare and ultra-rare allele frequency spectrum^8–10^. Through combining imputed genotypes with detailed phenotypes ascertained through national registries, FG holds the promise to provide particular insights into the so far little examined allele frequency spectrum between 0.1 and 2%, where both sequencing studies and GWAS have so far remained largely underpowered in relation to identifying associations with disease. This spectrum includes many coding variants with moderate to large effect sizes that can help identify causal genes in GWAS loci, provide mechanistic insights into disease pathologies, and potentially bridge rare and common diseases.

Here, we have leveraged the combined power of UKB and FG to investigate how rare and low-frequency variants in protein-coding regions of the genome contribute to the risk for human traits and diseases. Using data from a total of 653,219 individuals, we tested how approximately 48,000 coding variants identified in both biobanks through either whole-exome sequencing or genotype imputation associate with 744 distinct disease endpoints. Disease associations were compared against information from rare disease, biomarker and drug target resources and complemented by deep dives into distinct disease mechanisms of individual genes and coding variants. Our results showcase the benefits of combining large population cohorts to discover and replicate novel associations, explain disease mechanisms across a range of common and rare diseases, and shed light on a substantial gap in the allelic spectrum that neither genotyping nor sequencing studies have previously been able to address.

## Coding associations with human disease

**Extended Data Fig. 1:**
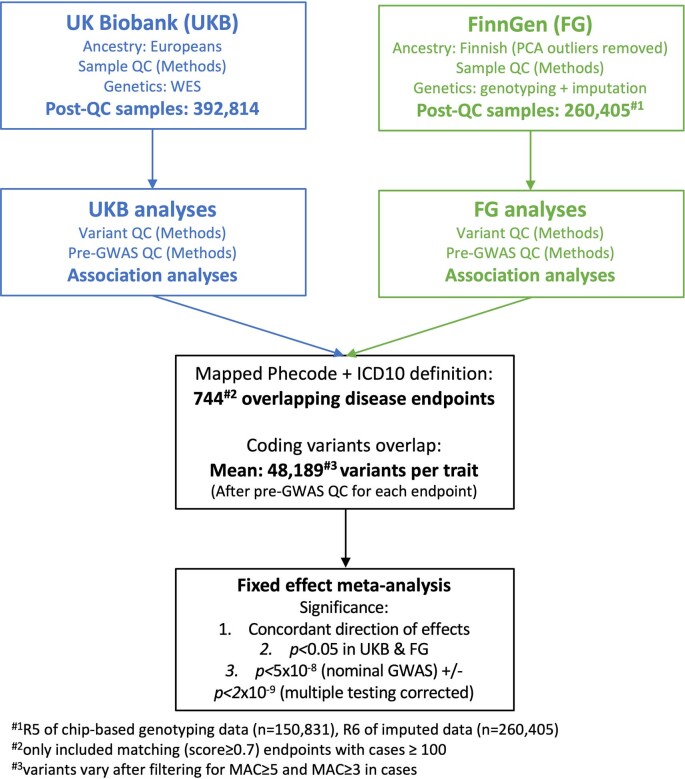
UKB and FG study overview.

**Extended Data Fig. 2:**
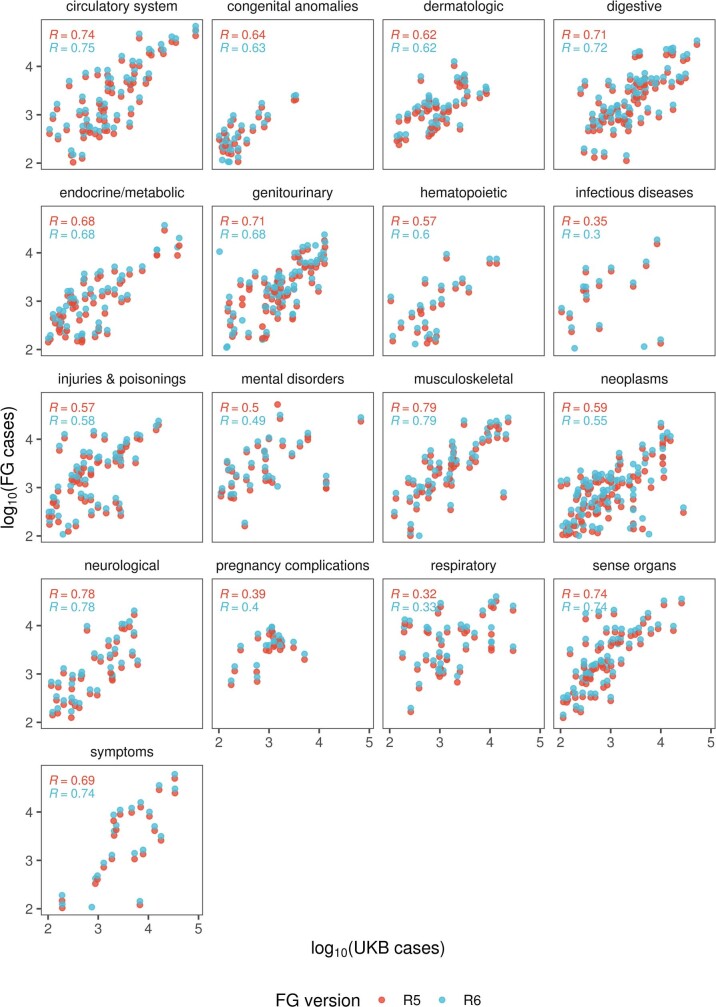
Case count comparison between UKB and FG across disease groups. Diseases within each group are listed in Supplementary Table 2. Only cases >100 in UKB/FG are included. *R: Spearman’s correlation for FG R5 (red) and R6 (blue)*.

An overview of the study design and basic demographics are provided in Extended Data Fig. 1, Supplementary Table 1. In brief, we systematically harmonised disease phenotypes across UKB and FG using Phecode and ICD10 mappings and retained 744 specific disease endpoints grouped into 580 disease clusters (Methods, Supplementary Table 2). Disease case counts relative to cohort size showed good correlations both overall between UKB and FG (Spearman’s *ρ* = 0.65, *P* < 5.3 × 10^−90^) and across distinct disease groups (Extended Data Fig. 2).

We performed coding-wide association studies (CWAS) across 744 disease endpoints over a mean of 48,189 (range: 25,309–89,993) (Methods, Supplementary Table 2) post-quality control coding variants across the allele frequency spectrum derived from whole-exome sequencing of 392,814 European ancestry individuals in UKB and meta-analysed these data with summary results from up to 260,405 individuals in FG (Methods, Supplementary Table 2).

**Fig. 1:**
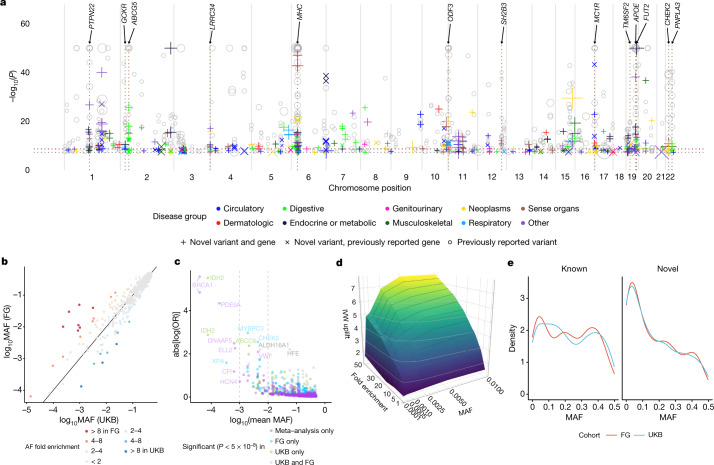
Coding genetic associations with disease. **a**, Summary of sentinel variant associations. Size of the point is proportional to effect size. −log_10_(*P*) capped at −log_10_(10^−50^). Labels highlight pleiotropic associations (≥5 trait clusters). Colours indicate disease groups. Shapes indicate novel and known (grey circles) associations. Dotted horizontal lines indicate –log_10_(2 × 10^−9^) (brown) and −log_10_(5 × 10^−8^) (grey). **b**, Comparison of sentinel variant MAF between UKB and FG data.** c**, Effect size against MAF of sentinel variants. Dashed lines indicate MAF of 0.1% (left) and 1% (right). Genes for coding variant associations with absolute effect size greater than 2 or MAF less than 0.1% are labelled. **d**, Surface plot of effects of cohort specific allele enrichment on inverse variant weighted (IVW) meta-analysis *z*-scores (IVW uplift) across MAFs (up to MAF 1%). Uplift is defined as the ratio of meta-analysed IVW *z*-score to the *z*-score of an individual study (details in Supplementary Information). **e**, Density plot of MAF for sentinel variants for known versus novel associations. Interactive Manhattan plot for novel associations and allelic enrichment surface plots are provided as Supplementary Files 1, 2.

**Extended Data Fig. 3:**
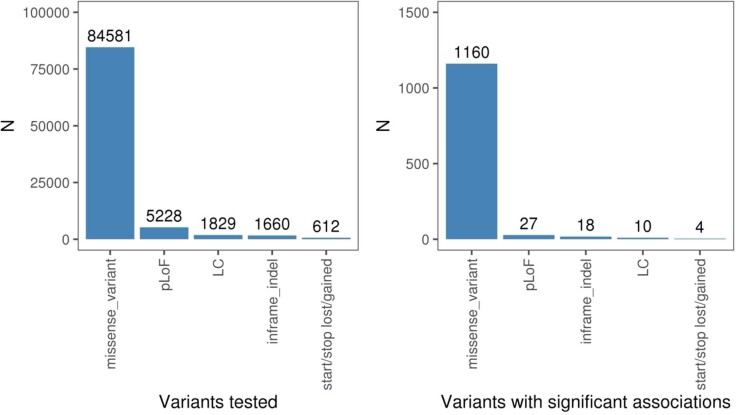
Distribution of variant annotation categories. **Left**: all variants tested. **Right**: variants with at least 1 significant association (*p* < 5 × 10^−8^). pLOF: predicted loss of function. LC: low confidence loss of function.

**Extended Data Fig. 4:**
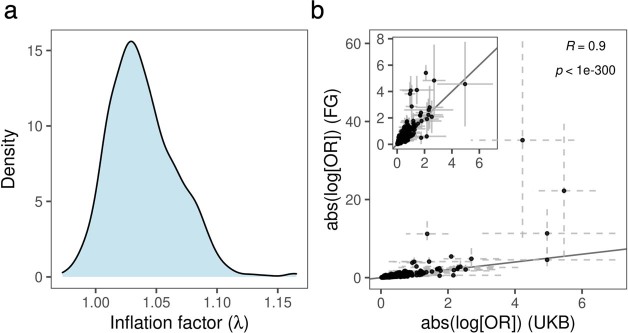
Inflation factors and FG-UKB effect size comparisons. (**a**) Distribution of inflation factors of CWAS meta-analysis. (**b**) Effect size comparison between UKB and FG. Inset: zoomed in on small effect sizes. *R: Spearman’s rank correlation (two-sided test), p =* 4.4 × 10^−351^.

We identified 975 associations (534 variants in 301 distinct regions across 148 disease clusters; 620 distinct region-disease cluster associations) meeting genome-wide significance (*P* < 5 × 10^−8^), and 717 associations (378 variants in 231 distinct regions across 121 disease clusters; 445 distinct region-disease cluster associations) at a conservative (Bonferroni) multiple testing threshold of *P* < 2 × 10^−9^ (correcting for the number of approximate independent tests) (Methods, Fig. 1a, Supplementary File 1 (interactive), Supplementary Table 3). The distributions of coding variant annotation categories were largely similar for variants with at least one significant association (*P* < 5 × 10^−8^) relative to all variants tested, with missense variants showing a higher fraction of significant variants than in-frame insertion–deletions or predicted loss-of-function (pLOF) variants (Extended Data Fig. 3). Inflation was well controlled with a mean genomic inflation factor of 1.04 (5th–95th percentiles: 1.00–1.09; Extended Data Fig. 4a). Effect sizes were generally well aligned between UKB and FG (Spearman’s *ρ* = 0.90, *P* < 10^−300^) (Extended Data Fig. 4b). Minor allele frequencies (MAFs) of lead variants correlated well overall between UKB and FG (Spearman’s *ρ* = 0.97, *P* < 10^−300^) (Fig. 1b), especially for variants with MAF > 1%, yet as expected^9^ from genetic differences between Finns and non-Finnish Europeans (NFEs) this correlation was reduced for variants with MAF < 1% (Spearman’s *ρ* = 0.32, *P* = 0.023).

Across all diseases, we found generally larger effect sizes for low frequency and rare variants (Fig. 1c). Of the 975 identified assocations, 387 (39.7% at *P* < 5 × 10^−8^, 270 out of 717 (37.7%) at *P* < 2 × 10^−9^) would not have been detected if analysed in UKB (61.5% at *P* > 5 × 10^−8^; 60.1% at *P* > 2 × 10^−9^) or FG (59.6% at *P* > 5 × 10^−8^; 58.6% at *P* > 2 × 10^−9^) alone. We found 13 associations (across 11 genes) with log odds ratios greater than 2 (Fig. 1c). Of these, 12 associated variants had MAF < 1%, and only the haemochromatosis variant rs1800562 showed frequency ranges traditionally interrogated in GWAS (MAF of 7.9% (UKB) and 3.7% (FG)). Several variants with large effect sizes reside in well studied disease genes such as *BRCA1* (breast cancer), *IDH2* (myeloid leukaemia), *VWF* (von Willebrand disease) or *HFE* (disorders or iron metabolism), proposing that carriers could benefit from clinical monitoring for associated conditions. Association testing within UKB and FG individually would have yielded 318 and 479 associations, respectively, at *P* < 5 × 10^−8^ **(**Supplementary Tables 4, 5). Thus, our combined approach using both biobanks increased the number of significant findings by approximately threefold for UKB and twofold for FG. Of the 318 and 479 significant sentinel variants in UKB and FG, 252 (72.6%) and 258 (53.9%) replicated at *P* < 0.05 in FG and UKB, respectively **(**Supplementary Tables 4, 5), further highlighting the strength of our approach to yield results that are more robust through replication than findings derived from a single biobank.

**Extended Data Fig. 5:**
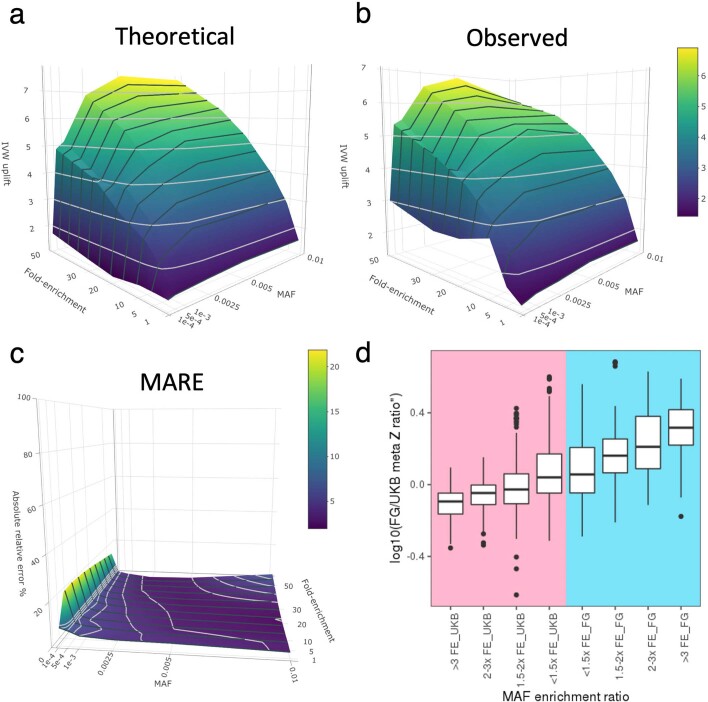
Surface plot of effects of cohort specific allele enrichment on inverse variant weighted meta-analysis z-scores (IVW uplift) across MAFs (up to MAF 1%). Uplift is defined as the ratio of meta-analysed IVW Z-score to the Z-score of an individual study. **(a)** Theoretically predicted IVW uplift. **(b)** Observed IVW uplift. **(c)** Median absolute relative error (MARE, %) between simulated and theoretical IVW uplift values. For each combination of MAF and allelic enrichment, we simulated 1000 datasets for two binary variables reflecting disease status for two studies. Study sample size and disease prevalence were fixed (matching values estimates from UKB and FG), genomic effects were randomly sampled from the set of positive effect sizes in UKB and FG (Supplementary Table 3), MAF was varied from 0.01% to 1% and allele enrichment (in the smaller study) ranged from 1 to 50. **(d)** Comparison of Z-scores for randomly subsetted UKB data meta-analysed with FinnGen (UKBxFG) against subsetted UKB meta-analysed with sample size matched UKB cohort (UKBxUKB') across allele fold enrichments for sentinel associations (Supplementary Table 3) with MAF<0.1. Y-axis (log_10_(FG/UKB meta Z ratio): log_10_(Z_UKBxFG_/Z_UKBxUKB'_). X-axis (MAF enrichment ratio): allelic fold enrichment (FE) where pink side denotes greater enrichment in UKB, blue side denotes greater enrichment in FG. Each box plot presents the median, first and third quartiles, with upper and lower whiskers representing 1.5x inter-quartile range above and below the third and first quartiles respectively. N for each boxplot from left to right: 88, 99, 212, 453, 213, 71, 126, 158.

Our study benefits from population enrichment of rare alleles in Finns versus NFEs (and vice versa) that increases the power for association discovery. Using a combination of theoretical analyses and empirical simulations, we show that by leveraging population-enriched variants we could increase inverse-variance weighted meta-analysis *Z*-scores and hence our ability to detect underlying associations (Supplementary Information). The gain in power from enriched alleles was present across a range of rare MAFs (0.01–1%), with the strongest power gain in the rare and ultra-rare MAF range of 0.01% to 0.25% (Fig. 1d, Extended Data Fig. 5, Supplementary Information, Supplementary Files 2a–c (interactive)). Notably, we demonstrate both theoretically and in practice that gains in power due to allele enrichment remain even after adjusting for power gains due to increased sample size (Supplementary Fig. 2 (MAF enrichment on *Z*-scores), Extended Data Fig. 5d). Of the sentinel variants, we found 73 (33 in UKB and 40 in FG) to be enriched by more than twofold and 23 (8 in UKB and 15 in FG) by more than fourfold relative to the other biobank (Fig. 1b, Supplementary Table 6). Most highly population-enriched variants are rare (MAF<1%) or low frequency (MAF 1–5%), whereby 20 out of 23 variants with more than fourfold population enrichment (13 in FG and 7 in UKB) had MAF <1% (Table 1, Supplementary Table 6). In comparison, 52 out of the total of 534 (9.7%) sentinel variants had MAF<1% in either UKB or FG, of which 15 and 23 were enriched by more than twofold in UKB and FG, respectively (Supplementary Table 6).

**Table 1:** Genes with sentinel variants enriched more than fourfold in either UKB or FG datasets

Gene	rs ID (amino acid change)	Chr	*A*_0_/*A*_1_	*A*_1_ frequency (UKB; FG (%))	log_2_ FE (FG/UKB)	OMIM gene–phenotype relationships	CWAS gene–phenotype relationships
*CHEK2*	rs17879961 (I200T) rs555607708^a^ (T410fs)	22	A/G AG/–	0.04%; 2.99% 0.24%; 0.64%	6.25 1.42	Cancer (breast, prostate, colorectal, osteosarcoma); Li–Fraumeni syndrome	rs17879961:G, benign meningeal neoplasm rs555607708:del (2.8× FG enriched), cancer (breast, thyroid, colorectal (benign)); uterine leiomyoma; ovarian cysts; PCOS
*DBH*	rs77273740 (R79W)	9	C/T	0.10%; 4.95%	5.69	Orthostatic hypotension	Hypertension (IA)
*PITX2*	rs143452464 (P41S)	4	G/A	0.02%; 1.01%	5.42	Anterior segment dysgenesis; Axenfeld–Rieger syndrome; ring dermoid of cornea	Arrythmia and AF
*SLC24A5*	rs1426654 (T111A)	15	A/G	0.09%; 1.13%	3.67	Skin, hair, eye pigmentation (dark); oculocutaneous albinism	Non-epithelial cancer of skin (other) (IA)
*CFHR5*	rs565457964 (E163fs)	1	C/CAA	0.32%; 3.96%	3.66	Nephropathy due to CFHR5 deficiency	Degeneration of macula and posterior pole of retina (IA)
*ANKH*	rs146886108 (R187Q)	5	C/T	0.72%; 0.07%	-3.28	Chondrocalcinosis; craniometaphyseal dysplasia	Type 2 diabetes mellitus (IA)
*ALDH16A1*	rs150414818 (P527R)	19	C/G	0.10%; 0.95%	3.23	–	Gout
*LRRK1*	rs41531245 (T967M)	15	C/T	0.09%; 0.76%	3.15	–	Contracture of palmar fascia; fasciitis; umbilical hernia
*CFI*	rs141853578 (G119R)	4	C/T	0.11%; 0.01%	−3.10	Atypical haemolytic uremic syndrome; age-related macular degeneration; CFI deficiency	Retinal disorders (other)
*FLG*	rs61816761 (R501*) rs138381300^a^ (S761fs)	1	G/A CACTG/−	2.45%; 0.29% 2.45%; 1.35%	−3.10 −0.85	Atopic dermatitis; ichthyosis vulgaris	rs61816761:A, dermatitis (other) rs138381300:del (1.8× UKB enriched), asthma; non-epithelial cancer of skin (other)
*SOS2*	rs72681869 (P191R)	14	G/C	1.09%; 0.15%	−2.84	Noonan syndrome	Hypertension (IA)
*XPA*	rs144725456 (H244R)	9	T/C	0.01%; 0.06%	2.61	Xeroderma pigmentosum	Non-epithelial cancer of skin (other)
*CDC25A*	rs146179438 (Q24H)	3	C/A	1.52%; 8.72%	2.52	−	Kidney and urinary stones (IA)
*F10*	rs61753266 (E142K)	13	G/A	0.33%; 1.83%	2.46	Factor X deficiency	PE and pulmonary heart disease (inverse association)
*TNXB*	rs61745355 (G2848R) rs10947230^a^ (R2704H) rs11507521 (T302A)	6	C/T C/T T/C	2.22%; 11.86% 5.96%; 14.75% 13.29%; 9.17%	2.42 2.31 −0.54	Ehlers–Danlos syndrome; vesicoureteral reflux	rs61745355:T, lymphoma rs10947230:T, lichen planus rs1150752:C, chronic hepatitis; other inflammatory liver diseases; atherosclerosis
*SLC39A8*	rs13107325 (A391T)	4	C/T	7.40%; 1.46%	−2.35	Congenital disorder of glycosylation	Shoulder lesions
*CLPTM1*	rs150484293 (L140F)	19	C/T	0.35%; 0.07%	−2.33	−	Dementia
*ELL2*	rs141299831 (S18L)	5	G/A	0.02%; 0.12%	2.29	−	Benign neoplasm of other and ill-defined parts of digestive system
*CASP7*	rs141266925 (F214L)	10	T/C	0.31%; 1.5%	2.29	−	Cataracts
*BRCA1*	rs80357906 (Q1777fs)	17	T/TG	0.001%; 0.01%	2.21	Cancer (breast, ovarian, pancreatic); Fanconi anaemia	Breast cancer
*SCN5A*	rs45620037 (T220I)	3	G/A	0.11%; 0.49%	2.20	Sudden infant death syndrome; dilated cardiomyopathy; arrythmia^b^	Arrythmia and AF
*CACNA1D*	rs1250342280 (F1943del)	3	CCTT/C	0.60%; 0.14%	−2.09	Primary aldosteronism, seizures, and neurologic abnormalities; sinoatrial node dysfunction and deafness	Hypertension
*WNT10A*	rs121908120 (F228I)	2	T/A	2.72%; 0.65%	−2.06	Odontoonychodermal dysplasia; Schopf–Schulz–Passarge syndrome; selective tooth agenesis	Follicular cysts of skin and subcutaneous tissue (IA)

^a^Other sentinel variants in the gene with greater than fourfold enrichment.

^b^Sudden infant death syndrome; atrial fibrillation; Brugada syndrome; progressive and non-progressive heart block; long QT syndrome, sick sinus syndrome; ventricular fibrillation.

All enrichment indicated by two-sided Fisher’s test; unadjusted *P* < 5 × 10^−5^.

AF, atrial fibrillation; Chr, chromosome; FE, fold enrichment; IA, inverse association; PCOS, polycystic ovarian syndrome; PE, pulmonary embolism; *A*_0_, reference allele; *A*_1_, alternative (effect) allele.

We systematically cross-referenced our results with previously described GWAS associations (via GWAS Catalog^11^ and PhenoScanner^12^) and disease relevance as reported in ClinVar^13^ (Methods). In total, we found that 216 out of 620 (34.8%) distinct region–disease cluster associations (at *P* < 5 × 10^−8^) had not previously been reported (130 out of 445 (29.2%) at *P* < 2 × 10^−9^). Out of the 216 distinct loci, 177 (104 out of 130 at *P* < 2 × 10^−9^) were in genes not previously mapped to the respective diseases (Fig. 1a, Supplementary Table 3, Supplementary File 1 (interactive)). Of the novel associations at GWAS significance (*P* < 5 × 10^−8^), roughly one-third had MAF < 5% in either UKB or FG and 15% had MAF < 1% (Supplementary Table 3). Notably, in UKB, 17% of known (19% in FG) and 31% of novel (28% in FG) associations had a MAF < 5%. Correspondingly, in UKB, 5% of known (6% in FG) and 15% of novel (10% in FG) associations had a MAF < 1%, highlighting the power gained through our approach especially in the low and rare allele frequency spectrum (Fig. 1e, Supplementary Table 3).

**Extended Data Fig. 6:**
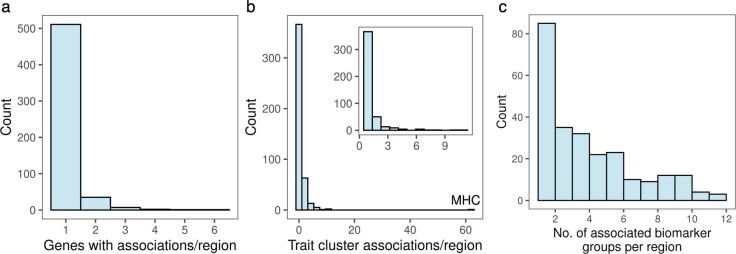
Histogram of genes with associations, disease and biomarker associations per region. **(a)** Number of genes with coding associations per region. Each disease cluster counted separately. MHC region excluded. **(b)** Number of associated trait clusters (*p* < 5 × 10^−8^) per region. Inset shows zoomed in x-scale between 0-12 trait cluster associations per region. **(c)** Number of associated biomarker groups per locus (*p*< 1 × 10^−6^). *MHC: Major Histocompatibility Complex*.

Mapping associations to genes, we found a total of 482 unique genes associated with the 148 disease clusters. Approximately 92% of the associated regions for each disease cluster (excluding the major histocompatibility complex (*MHC*) cluster) harbour a single gene with coding associations (Extended Data Fig. 6a). The majority of gene loci (81.2% at *P* < 5 × 10^−8^; *MHC* region counted as one locus) were associated with a single disease cluster (Extended Data Fig. 6b). Thirteen loci were associated with at least five trait clusters (at *P* < 5 × 10^−8^), including well established pleiotropic regions such as the *MHC*, *APOE*, *PTPN22*, *GCKR*, *SH2B3* and *FUT2* (Fig. 1a). For instance, in addition to a known association with breast cancer, we found variants in *CHEK2* to be associated with the risk of colorectal and thyroid cancers, uterine leiomyoma, benign meningeal tumours and ovarian cysts. Also, in addition to a known association with prostate hyperplasia, we found an *ODF3* missense variant (rs72878024, MAF = 7.5% (UKB) and 7.7% (FG)) to be associated with risk of uterine leiomyoma, benign meningeal tumour, lipoma and polyps in the female genital tract (Supplementary Table 3).

Harnessing the added power of UKB and FG, we were able to detect GWAS associations for rare variants previously only annotated as causal for single-gene diseases, establishing a disease relevance for these variants at the population level. Of the 534 distinct variants with significant disease associations in our study (*P* < 5 × 10^−8^), 152 (28.5%) had previously been linked to diseases in ClinVar. For 46 (30.3%) of these variants, the associated disease cluster matched with a previously reported phenotype in ClinVar. Notably, only 7 of these 46 variants (in *GJB2*, *ABCC6*, *BRCA1*, *SERPINA1*, *FLG*, *IDH2* and *MYOC*) had a previous annotation as either pathogenic or probably pathogenic (Supplementary Table 7), with 15 others annotated as benign. For the remaining 106 ClinVar-listed variants, 29 (27.4%) showed associations to conditions putatively related to those listed in ClinVar (Supplementary Table 7, Methods). For 17 variants, the medical relevance had been reported in ClinVar for the associated conditions, with 3 being classified as pathogenic or probably pathogenic and 14 classified either as benign or having ‘conflicting interpretation of pathogenicity’ for the associated trait (Supplementary Tables 3, 7). For instance, we found a rare missense variant annotated as showing conflicting pathogenicity in ClinVar in *VWF* (rs1800386:C; Tyr1584Cys; MAF = 0.44% (UKB) and 0.47% (FG)) to be associated with the risk of von Willebrand disease^13^ (log(odds ratio (OR)) = 2.09, *P* = 8.7 × 10^−9^). We also assessed the medical actionability of associated genes as defined in the latest American College of Medical Genetics and Genomics (ACMG) guidelines^14^ and found 15 coding variants with significant associations in 11 ACMG genes (Supplementary Table 7). Thirteen of these associations (one pathogenic (*BRCA1*), four conflicting evidence of pathogenicity and eight benign or probably benign) had prior ClinVar reports to a matching or putatively related condition, and for several our results proposed extended phenotypes. For example, we found that carriers of the rare missense variant rs370890951 (Ile1131Thr; MAF = 0.097% (UKB) and 0.29% (FG)) in *MYBPC3*, in which mutations cause hypertrophic cardiomyopathy, showed an approximately threefold increased risk (*P* = 9.8 × 10^−13^) for coagulation defects (Supplementary Tables 3, 7). Together, these findings highlight that population-scale analyses like ours can help refine pathogenicity assignments through contributing quantitative, rather than qualitative, information on relative disease risks for variant carriers, or establish an ‘allelic series’ for medically actionable genes.

Seventeen of the twenty-three genes with highly population-enriched sentinel variants (Table 1) were listed as disease genes at Online Mendelian Inheritance in Man (OMIM). Of these, ten (*CHEK2*, *DBH*, *SCL24A5*, *CFI*, *FLG*, *XPA*, *F10*, *BRCA1*, *SCN5A* and *CACNA1D*) showed associations with conditions identical or related to the respective Mendelian disease, revealing a relevance of the associated variants on the population level. For instance, we found the missense variant rs77273740 in *DBH* (enriched by more than 50-fold in FG)—a gene associated with orthostatic hypotension—to be associated with reduced risk of hypertension (log(OR) = −0.19, *P* = 1.3 × 10^−23^), and an in-frame deletion (rs1250342280) in *CACNA1D* (enriched by 4.3× in UKB)—a gene associated with primary aldosteronism—was associated with increased risk of hypertension (log(OR) = 0.19, *P* = 2.0 × 10^−8^) (Table 1).

## Biomedical insights through CWAS

We leveraged the coding variant associations identified in our study to generate biological insights for a range of distinct genes, pathways and diseases and in the following exemplify the broad utility of our resource with a set of selected use cases.

### Coagulation proteins in pulmonary embolism

**Extended Data Fig. 7:**
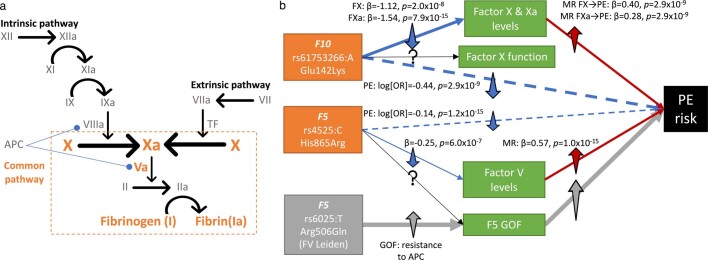
**(a) Simplified diagram of the coagulation cascade**. Factors (in roman numerals, “a” represents activated) with genetic association with PE highlighted in orange. Blue line (round end) indicates inhibitory effect of APC on VIIIa and Va. (**b**) Schematic of potential pathway from missense variants in *F5* and *F10* to PE risk. Factor V Leiden variant had null associations with F5 levels (β_F5 levels_=0.21, *p =* 0.091). Dashed blue lines suggest effect of the variants on PE risk which we assume under MR framework acts through factor levels (solid blue lines). Grey box and arrows represent known pathway for Factor V Leiden mutation. *GOF: Gain of function, APC: Activated protein C, MR: Mendelian randomisation, PE: Pulmonary embolism*.

We found known and novel associations with pulmonary embolism risk, including two rare variant associations (average MAF < 1%) in genes encoding components of the coagulation cascade at the convergent common pathway (Extended Data Fig. 7). For instance, we discovered a rare missense mutation in *F10*, enriched by approximately fivefold in FG (rs61753266:A; Glu142Lys; MAF = 0.33% (UKB) and 1.85% (FG)), and a venous thromboembolism risk-reducing variant in *F5* (rs4525:C, His865Arg; MAF = 27.2% (UKB) and 22.3% (FG)), to be protective against pulmonary embolism (log(OR)_*F10*_ = −0.44, *P*_*F10*_ = 2.9 × 10^−9^; log(OR)_*F5*_ = −0.14, *P*_*F5*_ = 1.2 × 10^−15^). The effects of these associations on the levels of their respective circulating factors and thromboembolic diseases, Mendelian randomization analyses that support developing drugs inhibiting factors V and X for pulmonary embolism and findings on additional clotting factors are discussed in (Supplementary Information ‘New roles for coagulation proteins in PE’).

### Rare variants yield mechanistic insights

**Fig. 2:**
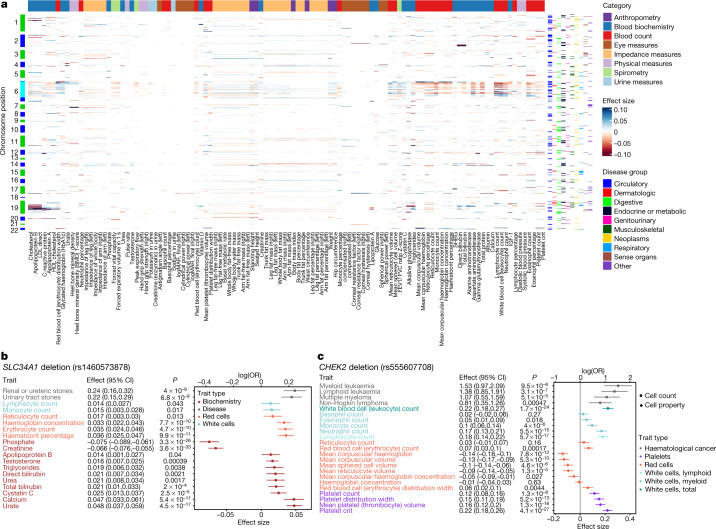
Biomarker associations with sentinel variants. **a**, Heat map of sentinel associations with biomarkers. Only significant associations (*P* < 10^−6^) are shown. Colours on the left axis indicate chromosomes, with cyan indicating the MHC region. Colours on the right axis indicate sentinel association with disease by group. Colours along the top indicate the category of biomarkers. **b**, Forest plot of associations (unadjusted regression effect estimates with 95% confidence intervals (CI)) between *SLC34A1* deletion (rs1460573878) with haematological and biochemistry biomarkers. associations with *P* < 0.05 are shown. **c**, Forest plot of associations (unadjusted regression effect estimate with 95% confidence interval (CI)) between *CHEK2* deletion (rs555607708) with haematological biomarkers. Unadjusted *P* values are shown. Disease associations *n* = 653,219 biologically independent samples. Specific sample sizes for biomarker associations are listed in Supplementary Table 8. IGF-1, insulin-like growth factor 1; LDL, low-density lipoprotein; SHBG, sex hormone binding globulin.

We interrogated the sentinel variants identified in this study for associations with 117 quantitative biomarkers spanning eight categories in UKB (Supplementary Table 8). At a multiple testing adjusted threshold of *P* < 1 × 10^−6^, we found 112 of the biomarkers to be associated with at least one of 433 sentinel variants across 247 regions (Fig. 2a, Supplementary Table 9, Supplementary Information). Ninety-five of the regions were associated with five or more biomarker categories (Extended Data Fig. 6c, Supplementary Table 9), including pleiotropic disease loci such as *MHC*, *APOE*, *GCKR*, *SH2B3* and *FUT2*.

#### SLC34A1 deletion and fluid biomarkers

Cross-referencing disease with biomarker associations provided mechanistic insights into novel findings. For instance, a low-frequency in-frame deletion in *SLC34A1* (rs1460573878; MAF = 2.6% (UKB) and 2.7% (FG); p.Val91_Ala97del) coding for the sodium phosphate cotransporter NPT2a expressed in proximal tubular cells was associated with increased risk of renal (log(OR) = 0.24, *P* = 4.0 × 10^−9^) and urinary tract stones (log(OR) = 0.21, *P* = 6.8 × 10^−9^). The deletion has previously been implicated in hypercalciuric renal stones^15,16^ and autosomal recessive idiopathic infantile hypercalcaemia^17^ in family studies. The variant is also associated with increased serum calcium (*β* = 0.047, *P* = 5.4 × 10^−11^) and reduced phosphate (*β* = −0.075, *P* = 3.3 × 10^−26^), consistent with a disrupted function or cell surface expression of the transporter^17^ (Fig. 2b). We further find associations with increased levels of serum urate (*β* = 0.048, *P* = 4.5 × 10^−17^), also suggesting an increased risk of uric acid stones. Additionally, we found associations with increased erythrocyte count (*β* = 0.035, *P* = 4.7 × 10^−10^), haemoglobin concentration (*β* = 0.033, *P* = 7.7 × 10^−10^) and haematocrit percentage (*β* = 0.036, *P* = 9.9 × 10^−11^), suggesting increased renal-driven erythropoiesis (Fig. 2b). Serum creatinine was not increased in carriers of the deletion (*β* = −0.07, *P* = 3.6 × 10^−33^), suggesting that renal function is not adversely affected in deletion carriers. Among 11,114 renal or ureteric, and 13,319 urinary tract stone cases, we identified 735 (renal or ureteric) and 863 (urinary tract) carriers of the deletion who may benefit from clinical interventions targeting NPT2A-related pathways and monitoring for disturbed biochemical and haematological biomarkers.

#### CHEK2 deletion and haematological signs

A frameshift deletion in *CHEK2* (rs555607708; MAF = 0.64% (FG), 0.24% (UKB)) that increases breast cancer risk has also been previously implicated in myeloproliferative neoplasms through GWAS^18^ and lymphoid leukaemia in a candidate variant study^19^. Consistently, we found nominally significant associations with risks of both, myeloid (log(OR) = 1.52, *P* = 9.5 × 10^−8^) and lymphoid (log(OR) = 1.38, *P* = 3.1 × 10^−7^) leukaemia, but also multiple myeloma (log(OR) = 1.07, *P* = 5.1 × 10^−5^) and non-Hodgkin lymphoma (log(OR) = 0.81, *P* = 4.7 × 10^−4^). Association of rs555607708 with clinical haematology traits showed statistically significant associations with increased blood cell counts for both myeloid (leukocytes, neutrophils and platelets at *P* < 1 × 10^−6^; monocyte and erythrocytes at *P* < 1 × 10^−3^) and lymphoid (lymphocytes, *P* = 5.7 × 10^−17^) lineages (Fig. 2c). Furthermore, we found associations with increased mean platelet volume (*P* = 1.3 × 10^−16^) and platelet distribution width (*P* = 5.2 × 10^−13^), consistent with increased platelet activation and previous associations of mean platelet volume and platelet distribution width with chronic myeloid leukaemia^20^. We also found associations with decreased mean corpuscular haemoglobin (*P* = 7.8 × 10^−12^) and mean corpuscular volume (*P* = 5.3 × 10^−10^), suggesting that predisposition to haematological cancers by loss of *CHEK2* function is accompanied by a microcytic red blood cell phenotype (Fig. 2c).

### Coding associations aid drug development

We cross-referenced genes with significant coding variant associations with drug targets^21^. We found 66 genes with trait cluster associations that are the targets of either approved drugs (26 genes) or drugs currently being tested in clinical trials (40 genes), 14 of which are in phase III trials (Supplementary Table 10). We found a statistically significant enrichment of significant genes in our study that were also approved drug targets (26 out of 482, compared with a background of 569 approved targets out of 19,955 genes, OR = 1.9, *P* = 0.0024), which is in line with previous estimates of a higher success rate for drug targets supported by genetics^22,23^. Sensitivity analyses using more stringent association *P*-value thresholds further increased these probability estimates (*P* = 5 × 10^−9^ (OR = 2.3, *P* = 0.00070); *P* = 5 × 10^−10^ (OR = 2.5, *P* = 0.00037)), supporting previous observations of higher likelihood of therapeutic success with stronger genetic associations (Supplementary Table 11). Specific examples are highlighted in the Supplementary Information.

Atrial fibrillation (AF). GWAS have yielded a sizeable number of loci^24,25^. We chose AF to exemplify how results from our study can further elucidate the genetics and biological basis of one distinct human trait. Notably, we report several coding variant associations (Supplementary Table 3) in which prior GWAS^24,25^ had fallen short for resolving GWAS loci to coding genes and explaining disease mechanisms.

#### METTL11B methylase missense variant in AF

The AF GWAS sentinel variant rs72700114 is an intergenic variant located between *METTL11B* and *LINC01142* with no obvious candidate gene^24–26^. Our study revealed that a low-frequency missense variant in the methylase *METTL11B* (rs41272485:G; Ile127Met; MAF = 3.9% (UKB) and 3.8% (FG)) was associated with increased AF risk (log(OR) = 0.14, *P* = 4.0 × 10^−11^). This variant locates to the enzyme’s *S*-adenosylmethionine–*S*-adenosyl-l-homocysteine ligand-binding site^27^ and is expected to perturb methylation of other AF risk genes with N-terminal (Ala/Pro/Ser)-Pro-Lys methylation motifs that are enriched in cardiomyocytes (Methods, Supplementary Table 12, Supplementary Results), which probably explains the association.

#### Rare variants and ion channel AF loci

Within the *SCN5A–SCN10A* locus, we replicated a common missense variant in *SCN10A* (rs6795970:A; Ala1073Val; MAF = 40.0% (UKB) and 44.6% (FG)) that was previously described as prolonging cardiac conduction^28^. Additionally, we found associations with reduced AF risk (log(OR) = −0.06, *P* = 2.1 × 10^−12^), reduced pulse rate (*β* = −0.02, *P* = 4.8 × 10^−18^), and a suggestive signal for increased risk of atrioventricular block (log(OR) = 0.10, *P* = 1.9 × 10^−7^). It is thus tempting to speculate that loss of function of Na_V_1.8—the sodium channel encoded by *SCN10A*—blunts the effects of vagus nerve activity on the atria. In addition, we found a rare, enriched missense variant in FG (rs45620037:A; Thr220Ile; MAF = 0.11% (UKB) and 0.47% (FG); SIFT = 0.03, PolyPhen = 0.96) in *SCN5A*—which encodes the cardiac sodium channel Na_V_1.5—to be associated with decreased risk of AF (log(OR) = −0.65, *P* = 1.3 × 10^−12^). This missense variant resides within the voltage sensing segment of SCN5A, causes a partial loss of function of the Na_V_1.5 channel in atrial cells and has been associated with dilated cardiomyopathy^29^ and conduction defects including sick sinus syndrome and atrial standstill^30^ in family studies with bradycardic changes. Consistently, we found a nominal association with reduced pulse rate (*β* = −0.078, *P* = 0.023), suggesting that protective effects of the variant will be most beneficial for the common tachycardic form of AF through reducing pulse rate. The *SCN10A* and *SCN5A* variants found here are probably both moderators of AF risk that act by different mechanisms. Potential mechanisms underlying further AF loci are described in Supplementary Discussion.

#### Genetic effects underlying AF and pulse

**Fig. 3:**
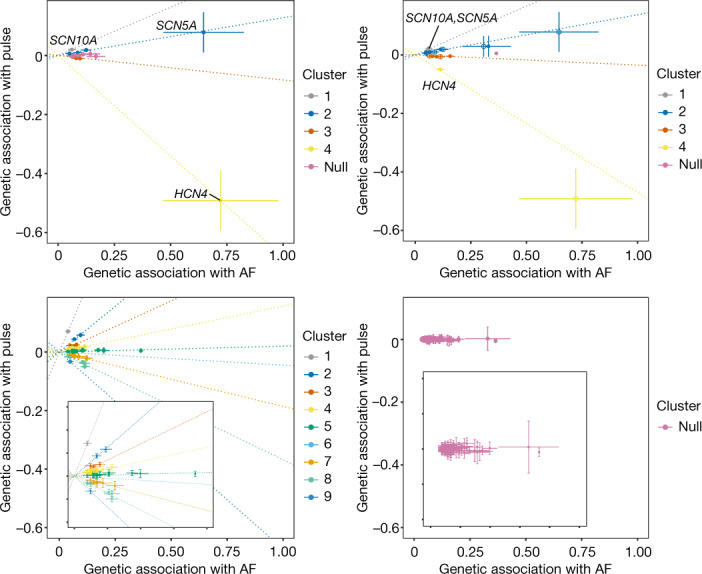
Genetic and functional insights into atrial fibrillation. Clustered Mendelian randomization plot of association of atrial fibrillation loci with pulse rate. Only variants with cluster inclusion probability greater than 0.7 are included. Top left, CWAS loci (sentinels). Top right, overlapping CWAS and atrial fibrillation GWAS loci. Bottom left, all atrial fibrillation GWAS loci from Nielsen et al.^49^ (with zoomed inset). Bottom right, all atrial fibrillation GWAS loci with permuted pulse (null, with zoomed inset).

To further evaluate the hypothesis that distinct genetic mechanisms underlying AF risk inversely modulate pulse rate, we adjusted the clustered Mendelian randomization^31^ (MR-Clust) algorithm to better accommodate rare-variants. We then related expectation maximization clustering of AF associated variants with homogenous directional effects on pulse rate (Methods). We found clusters of CWAS AF sentinel variants in *SCN10A* (rs6795970) and *HCN4* (rs151004999) as two genetic components of AF that can increase and decrease pulse rate, respectively (Fig. 3, Supplementary Table 13). Identifying components of AF with diverging directionality on pulse rate matches clinical observations that AF can be caused by both tachycardia and bradycardia^32^. Using sentinel variants from a recent AF GWAS^24^ for sensitivity analyses yielded concordant patterns. By integrating CWAS and GWAS sentinel variants for AF we found additional clusters with differing effects on pulse rate. Expectedly, within the AF GWAS loci^24^, the two rare missense alleles in *HCN4* (rs151004999:T, log(OR) = 0.72) and *SCN5A* (rs45620037:A, log(OR) = −0.65) identified in our study had much larger effect sizes on AF risk than the respective non-coding sentinel GWAS variants (rs74022964:T (*HCN4* locus), log(OR) = 0.12; rs6790396:C (*SCN5A* and *SCN10A* locus), log(OR) = −0.058) (Fig. 3).

#### Functional effects of PITX2c(Pro41Ser)

**Extended Data Fig. 8:**
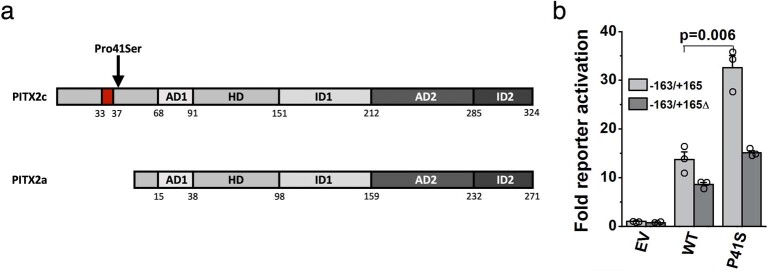
In vitro functional effects of the PITX2c Pro41Ser variant (rs143452464). (**a**) Schematic of the location of the Pro41Ser variant in PITX2c as compared with the PITX2a splicing isoform. Numbers below each row indicate AA number from N-terminal (left) to C-terminal (right). AD1: common sequence, HD: homeodomain, ID1: transcriptional inhibitory domain 1, AD2: second common sequence, ID2: transcriptional inhibitory domain 2. Pro41Ser lies within the terminal domain (grey), near the 5-amino acid LAMAS (single amino acid code) sequence (33 to 37 red), which is important for transcriptional activity of the N-terminal of PITX2c. (**b**) Reporter gene assays: TM-1 cells (Transformed human trabecular meshwork cells) were co-transfected with a firefly luciferase reporter plasmid containing a PITX2c binding element, a β-galactosidase control vector and expression vector for PTIX2c (wild-type, Pro41Ser (P41S) or empty control vector (EV)). The activity of firefly luciferase upon activation by PITX2c (n = 3 transfections per condition) was normalized to β-galactosidase. Experiments with a truncated reporter construct ("−163/+165Δ"), containing a deletion of 8bp within the predicted PITX2 binding site, are shown as additional control. Data are presented as mean values +/− SEM, unadjusted *p*-value derived from two-sided t-test (EVΔ vs EV: 0.122; WTΔ vs WT: 0.074; P41SΔ vs P41S: 0.018; WT vs EV: 0.015; P41S vs WT: 0.0056; WTΔ vs EVΔ: 0.002; P41SΔ vs WTΔ: 0.0005).

Finally, we found a rare missense variant in *PITX2* as associated with increased risk of AF (log(OR) = 0.38, *P* = 1.1 × 10^−9^). This variant is enriched nearly 50-fold in FG (rs143452464:A; Pro41Ser; MAF = 0.023% (UKB) and 1.1% (FG)) and was independently identified in a French family with AF (Supplementary Information), whereas GWAS had linked intergenic variants between *PITX2* and *FAM241A* to AF risk. PITX2 is a bicoid type homeobox transcription factor previously assumed to play a role in cardiac rhythm control^33^. The Pro41Ser variant lies in the N-terminal domain that is only present in the PITX2c isoform expressed in cardiac muscle. In reporter assays comparing the ability of PITX2c wild-type and Pro41Ser protein constructs to transactivate a luciferase reporter plasmid containing a putative PITX2c-binding element, PITX2c(Pro41Ser) showed an approximately 2.4-fold higher activation of the reporter than the wild type (*P* = 0.006, Extended Data Fig. 8). This effect was abrogated upon deletion of the putative PITX2c-binding site. In cultured cardiac muscle HL-1 cells, the Pro41Ser mutation increased the transcription of several presumed PITX2c target genes (Supplementary Table 14, Supplementary Information). Together, these results are consistent with a putative gain-of-function mechanism of Pro41Ser on PITX2c transactivation potential and AF risk.

## Discussion

Here we have conducted the largest association study of protein-coding genetic variants so far against hundreds of disease endpoints ascertained from two massive population biobanks, UK Biobank and FinnGen. We report novel disease associations, most notably in the rare and low-allelic frequency spectrum, replicate and assign putative causalities to many previously reported GWAS associations, and leverage the insights gained to elucidate disease mechanisms, demonstrating that the step from association to biological insight may be considerably shorter for coding variant association studies than it has traditionally been for GWAS. In addition to a substantial gain in power over previous studies, our analyses benefit from replication between two population cohorts, increasing the robustness of our findings and setting the stage for future similar studies in ethnically more diverse populations.

Notably, our study identifies both pathogenic variants residing in monogenic disease genes to impact the risk for related complex conditions as well as new, probably causal sentinel variants within GWAS loci in genes with known and novel biological roles in the respective GWAS trait. With this, our study is one of the first to help bridge the gap between common and rare disease genetics across a broad range of conditions and provides support for the hypothesis that the genetic architecture of many diseases is continuous^1^. Of the 975 associations identified in our study, 145 are driven by unique variants in the so far little-interrogated rare and low-allelic frequency spectrum between 0.1 and 2% that neither GWAS nor sequencing studies have been able to thoroughly interrogate across a range of diseases and that is hypothesized to contribute to the ‘missing heritability’ of many human diseases^34^.

Our approach benefits considerably from the Finnish genetic background, where certain alleles are stochastically enriched to unusually high allele frequencies^6–8^, at times exceeding population frequencies in the UK Biobank by more than 50-fold. Our theoretical and empirical results suggest the increasing utility of enriched variants for identifying associations quantitatively towards lower allelic frequencies. Notably, we identify the most prominent relative power gain in the rarest variant frequency spectrum, highlighting a role for sequencing studies and integrating additional population cohorts with enriched variants for identifying novel disease associations at scale. We identify several alleles with comparatively high effect sizes and a prevalence in the population that warrants follow-up, both experimentally as well as potentially directly in clinical settings to help improve disease outcomes. For instance, our data propose that 6.5% of UKB and FG participants with kidney or urinary tract stones, conditions debilitating more than 15% of men and 5% of women by 70 years of age^35^, carry a deletion in *SLC34A1*. Monitoring patients for the clinical biomarkers identified here as associated with this deletion might help to differentiate aetiologies and guide individualized treatments. Similarly, coding variant associations identified in our study may serve as an attractive source to generate hypotheses for drug discovery programs. Our results support previous studies^22,23^ that drug targets supported by human genetics have an increased likelihood of success, which can be considered particularly high when the genetic effect on a drug target closely mimics that of a pharmacological intervention^36^.

Our results foreshadow the discovery of many additional coding and non-coding associations from cross-biobank analyses at even larger sample sizes. With the continued growth of population biobanks with comprehensive health data in non-European populations, the emergence of more and more cost-effective technologies for sequencing and genotyping, and computational advances to analyse genetic and non-genetic data at scale, future studies will be able to assess the genetic contribution to health and disease at even finer resolution.

## Methods

### Samples and participants

UKB is a UK population study of approximately 500,000 participants aged 40–69 years at recruitment^2^. Participant data (with informed consent) include genomic, electronic health record linkage, blood, urine and infection biomarkers, physical and anthropometric measurements, imaging data and various other intermediate phenotypes that are constantly being updated. Further details are available at https://biobank.ndph.ox.ac.uk/showcase/. Analyses in this study were conducted under UK Biobank Approved Project number 26041. Ethic protocols are provided by the UK Biobank Ethics Advisory Committee (https://www.ukbiobank.ac.uk/learn-more-about-uk-biobank/about-us/ethics).

FG is a public-private partnership project combining electronic health record and registry data from six regional and three Finnish biobanks. Participant data (with informed consent) include genomics and health records linked to disease endpoints. Further details are available at https://www.finngen.fi/. More details on FG and ethics protocols are provided in Supplementary Information. We used data from FG participants with completed genetic measurements (R5 data release) and imputation (R6 data release). FinnGen participants provided informed consent for biobank research. Recruitment protocols followed the biobank protocols approved by Fimea, the National Supervisory Authority for Welfare and Health. The Coordinating Ethics Committee of the Hospital District of Helsinki and Uusimaa (HUS) approved the FinnGen study protocol Nr HUS/990/2017. The FinnGen study is approved by Finnish Institute for Health and Welfare.

### Disease phenotypes

FG phenotypes were automatically mapped to those used in the Pan UKBB (https://pan.ukbb.broadinstitute.org/) project. Pan UKBB phenotypes are a combination of Phecodes^37^ and ICD10 codes. Phecodes were translated to ICD10 (https://phewascatalog.org/phecodes_icd10, v.2.1) and mapping was based on ICD-10 definitions for FG endpoints obtained from cause of death, hospital discharge and cancer registries. For disease definition consistency, we reproduced the same Phecode maps using the same ICD-10 definitions in UKB. In particular, we expertly curated 15 neurological phenotypes using ICD10 codes. We retained phenotypes where the similarity score (Jaccard index: ICD10_FG_ ∩ ICD10_UKB_ / ICD10_FG_ ∪ ICD10_UKB_) was >0.7 and additionally excluded spontaneous deliveries and abortions.

Phecodes and ICD10 coded phenotypes were first mapped to unified disease names and disease groups using mappings from Phecode, PheWAS and icd R packages followed by manual curation of unmapped traits and diseases groups, mismatched and duplicate entries. Disease endpoints were mapped to Experimental Factor Ontology (EFO) terms using mappings from EMBL-EBI and Open Targets based on exact disease entry matches followed by manual curation of unmapped traits.

Disease trait clusters were determined through first calculating the phenotypic similarity via the cosine similarity, then determining clusters via hierarchical clustering on the distance matrix (1-similarity) using the Ward algorithm and cutting the hierarchical tree, after inspection, at height 0.8 to provide the most semantically meaningful clusters.

### Genetic data processing

#### UKB genetic QC

UKB genotyping and imputation were performed as described previously^2^. Whole-exome sequencing data for UKB participants were generated at the Regeneron Genetics Center (RGC) as part of a collaboration between AbbVie, Alnylam Pharmaceuticals, AstraZeneca, Biogen, Bristol-Myers Squibb, Pfizer, Regeneron and Takeda with the UK Biobank. Whole-exome sequencing data were processed using the RGC SBP pipeline as described^3,38^. RGC generated a QC-passing ‘Goldilocks’ set of genetic variants from a total of 454,803 sequenced UK Biobank participants for analysis. Additional quality control (QC) steps were performed prior to association analyses as detailed below.

#### FG genetic QC

Samples were genotyped with Illumina and Affymetrix arrays (Thermo Fisher Scientific). Genotype calls were made with GenCall and zCall algorithms for Illumina and AxiomGT1 algorithm for Affymetrix data. Sample, genotyping as well as imputation procedures and QC are detailed in Supplementary Information.

#### Coding variant selection

GnomAD v.2.0 variant annotations were used for FinnGen variants^39^. The following gnomAD annotation categories are included: pLOF, low-confidence loss-of-function (LC), in-frame insertion–deletion, missense, start lost, stop lost, stop gained. Variants have been filtered to imputation INFO score > 0.6. Additional variant annotations were performed using variant effect predictor (VEP)^40^ with SIFT and PolyPhen scores averaged across the canonical annotations.

### Disease endpoint association analyses

For optimized meta-analyses with FG, analyses in UKB were performed in the subset of exome-sequence UKB participants with white European ancestry for consistency with FG (*n* = 392,814). We used REGENIE v1.0.6.7 for association analyses via a two-step procedure as detailed in ref. ^41^. In brief, the first step fits a whole genome regression model for individual trait predictions based on genetic data using the leave one chromosome out (LOCO) scheme. We used a set of high-quality genotyped variants: MAF > 5%, MAC > 100, genotyping rate >99%, Hardy–Weinberg equilibrium (HWE) test *p* > 10^−15^, <5% missingness and linkage-disequilibrium pruning (1,000 variant windows, 100 sliding windows and *r*^2^ < 0.8). Traits where the step 1 regression failed to converge due to case imbalances were subsequently excluded from subsequent analyses. The LOCO phenotypic predictions were used as offsets in step 2 which performs variant association analyses using the approximate Firth regression detailed in ref. ^41^ when the *P* value from the standard logistic regression score test is below 0.01. Standard errors were computed from the effect size estimate and the likelihood ratio test *P*-value. To avoid issues related to severe case imbalance and extremely rare variants, we limited association test to phenotypes with >100 cases and for variants with MAC ≥ 5 in total samples and MAC ≥ 3 in cases and controls. The number of variants used for analyses varies for different diseases as a result of the MAC cut-off for different disease prevalence. The association models in both steps also included the following covariates: age, age^2^, sex, age*sex, age^2^*sex, first 10 genetic principal components (PCs).

Association analyses in FG were performed using mixed model logistic regression method SAIGE v0.39^42^. Age, sex, 10 PCs and genotyping batches were used as covariates. For null model computation for each endpoint each genotyping batch was included as a covariate for an endpoint if there were at least 10 cases and 10 controls in that batch to avoid convergence issues. One genotyping batch need be excluded from covariates to not have them saturated. We excluded Thermo Fisher batch 16 as it was not enriched for any particular endpoints. For calculating the genetic relationship matrix, only variants imputed with an INFO score >0.95 in all batches were used. Variants with >3% missing genotypes were excluded as well as variants with MAF < 1%. The remaining variants were linkage-disequilibrium pruned with a 1-Mb window and *r*^2^ threshold of 0.1. This resulted in a set of 59,037 well-imputed not rare variants for GRM calculation. SAIGE options for null computation were: “LOCO=false, numMarkers=30, traceCVcutoff=0.0025, ratioCVcutoff=0.001”. Association tests were performed phenotypes with case counts >100 and for variants with minimum allele count of 3 and imputation INFO >0.6 were used.

We additionally performed sex-specific associations for a subset of gender-specific diseases (60 female diseases and in 50 disease clusters, 14 male diseases and in 13 disease clusters) in both FG and UKB using the same approach without inclusion of sex-related covariates (Supplementary Table 2)

We performed fixed-effect inverse-variance meta-analysis combining summary effect sizes and standard errors for overlapping variants with matched alleles across FG and UKB using METAL^43^.

### Definition and refinement of significant regions

To define significance, we used a combination of (1) multiple testing corrected threshold of *P* < 2 × 10^−9^ (that is, 0.05/(approximately 26.8 × 10^6^), the sum of the mean number of variants tested per disease cluster)), to account for the fact that some traits are highly correlated disease subtypes, (2) concordant direction of effect between UKB and FG associations, and (3) *P* < 0.05 in both UKB and FG.

We defined independent trait associations through linkage-disequilibrium-based (*r*^2^ = 0.1) clumping ±500 kb around the lead variants using PLINK^44^, excluding the HLA region (chr6:25.5-34.0Mb) which is treated as one region due to complex and extensive linkage-disequilibrium patterns. We then merged overlapping independent regions (±500 kb) and further restricted each independent variant (*r*^2^ = 0.1) to the most significant sentinel variant for each unique gene. For overlapping genetic regions that are associated with multiple disease endpoints (pleiotropy), to be conservative in reporting the number of associations we merged the overlapping (independent) regions to form a single distinct region (indexed by the region ID column in Supplementary Table 3).

### Cross-reference with known associations

We cross-referenced the sentinel variants and their proxies (*r*^2^ > 0.2) for significant associations (*P* < 5 × 10^−8^) of mapped EFO terms and their descendants in GWAS Catalog^11^ and PhenoScanner^12^. To be more conservative with reporting of novel associations, we also considered whether the most-severe associated gene in our analyses were reported in GWAS Catalog and PhenoScanner. In addition, we also queried our sentinel variants in ClinVar^13^ to define known associations with rarer genetic diseases and further manually curated novel associations (where the association is a novel variant association and a novel gene association) for previous genome-wide significant (*P* < 5 × 10^−8^) associations.

To assess medical actionability of associated genes, we cross-referenced the associated genes with the latest ACMG v3. (75 unique genes linked to 82 conditions, linked to cancer (*n* = 28), cardiovascular (*n* = 34), metabolic (*n* = 3), or miscellaneous conditions (*n* = 8)). This list was supplemented by 20 ‘ACMG watchlist genes’^14^ for which evidence for inclusion to ACMG 3.0 list was considered too preliminary based on either technical, penetrance or clinical management concerns

### Biomarker associations of lead variants

For the lead sentinel variants, we performed association analyses using the two-step REGENIE approach described above with 117 biomarkers including anthropometric traits, physical measurements, clinical haematology measurements, blood and urine biomarkers available in UKB (detailed in Supplementary Table 8). Additional biochemistry subgroupings were based on UKB biochemistry subcategories: https://www.ukbiobank.ac.uk/media/oiudpjqa/bcm023_ukb_biomarker_panel_website_v1-0-aug-2015-edit-2018.pdf

### Drug target mapping and enrichment

We mapped the annotated gene for each sentinel variant to drugs using the therapeutic target database (TTD)^21^. We retained only drugs which have been approved or are in clinical trial stages. For enrichment analysis of approved drugs with genetic associations, we used Fisher’s exact test on the proportion of significant genes targeted by approved drug against a background of all approved drugs in TTD^21^ (*n* = 595) and 20,437 protein coding genes from Ensembl annotations^45^.

### Mendelian randomization analyses

#### *F5* and *F10* effects on pulmonary embolism

The missense variants rs4525 and rs61753266 in *F5* and *F10* genes were taken as genetic instruments for Mendelian randomization analyses. To assess potential that each factor level is causally associated with pulmonary embolism we used two-sample Mendelian randomization using summary statistics, with effect of the variants on their respective factor levels obtained from previous large scale (protein quantitative trait loci) pQTL studies^46,47^. Let ${\beta }_{{XY}}$ denote the estimated causal effect of a factor level on pulmonary embolism risk and ${\beta }_{X}$, ${\beta }_{Y}$ be the genetic association with a factor level (FV, FX or FXa) and pulmonary embolism risk respectively. Then, the Mendelian randomization ratio-estimate of ${\beta }_{{XY}}$ is given by:

$${\beta }_{{XY}}=\frac{{\beta }_{Y}}{{\beta }_{X}}$$

where the corresponding standard error ${\rm{se}}({\beta }_{{XY}})$, computed to leading order, is:

$${\rm{se}}({\beta }_{{XY}})=\frac{{\rm{se}}({\beta }_{Y})}{\left|{\beta }_{X}\right|}$$

#### Clustered Mendelian randomization

To assess evidence of several distinct causal mechanisms by which AF may influence pulse rate (PR) we used MR-Clust^31^. In brief, MR-Clust is a purpose-built clustering algorithm for use in univariate Mendelian randomization analyses. It extends the typical Mendelian randomization assumption that a risk factor can influence an outcome via a single causal mechanism^48^ to a framework that allows one or more mechanisms to be detected. When a risk-factor affects an outcome via several mechanisms, the set of two-stage ratio-estimates can be divided into clusters, such that variants within each cluster have similar ratio-estimates. As shown in^31^, two or more variants are members of the same cluster if and only if they affect the outcome via the same distinct causal pathway. Moreover, the estimated causal effect from a cluster is proportional to the total causal effect of the mechanism on the outcome. We included variants within clusters where the probability of inclusion >0.7. We used MR-Clust algorithm allowing for singletons/outlier variants to be identified as their own ‘clusters’ to reflect the large but biologically plausible effect sizes seen with rare and low-frequency variants.

### Bioinformatic analyses for *METTL11B*

We searched [Ala/Pro/Ser]-Pro-Lys motif containing proteins using the ‘peptide search’ function on UniProt^49^, filtering for reviewed Swiss-Prot proteins and proteins listed in Human Protein Atlas^50^ (HPA) (*n* = 7,656). We obtained genes with elevated expression in cardiomyocytes (*n* = 880) from HPA based on the criteria: ‘cell_type_category_rna: cardiomyocytes; cell type enriched, group enriched, cell type enhanced’ as defined by HPA at https://www.proteinatlas.org/humanproteome/celltype/Muscle+cells#cardiomyocytes (accessed 20th March 2021) with filtering for those with valid UniProt IDs (Swiss-Prot, *n* = 863). Enrichment test was performed using Fisher’s exact test. Additionally, we performed enrichment analyses using any [Ala/Pro/Ser]-Pro-Lys motif positioned within the N-terminal half of the protein (*n* = 4,786).

**Additional methods** Additional methods on further FinnGen QC; theoretical description and simulation of the effect of MAF enrichment on inverse-variance weighted (IVW) meta-analysis *Z*-scores; and functional characterization of PITX2c(Pro41Ser) are provided in the Supplementary Information.

### Reporting summary

Further information on research design is available in the Nature Research Reporting Summary linked to this paper.

## Online content

Any methods, additional references, Nature Research reporting summaries, source data, extended data, supplementary information, acknowledgements, peer review information; details of author contributions and competing interests; and statements of data and code availability are available at 10.1038/s41586-022-04394-w.

## Supplementary information

Supplementary Information This file contains Supplementary Methods, results and discussions, Supplementary Figs. 1–6, full legends for Supplementary Tables 1–14, Biobank contributions to FinnGen, FinnGen ethics statement details, a list of FinnGen consortium contributors, PITX2 Function Study Group contributors, a list of Biogen Biobank Team contributors and Supplementary References.

Reporting Summary

Supplementary File 1 Interactive Manhattan plot summary of novel sentinel associations. Size of the point is proportional to effect size. -log_10_(p) capped at -log_10_(10^-50^). Colours indicate disease groups (click [select/deselect trace]/double click [isolate one trace] on the legend to toggle selection). “+” indicate novel variant and gene, “×” indicate novel variant (r2<0.2) not reported in GWAS Catalog/PhenoScanner for the disease. Dotted horizontal lines indicate -log_10_(2x10^-9^) [brown] and -log_10_(5x10^-8^) [grey]. Hover over the points for detailed information and double click/click on colors/shape in the legends to filter on select groups. Tooltip is available at top right for additional interactive options including: zooming and panning, selection, toggling multiple highlighting of nearby regions on hover and saving as static images.

Supplementary Tables Supplementary Tables 1–14 – see Supplementary Information document for full table descriptions.

Peer Review File

Supplementary File 2 This zipped file contains Supplementary Files 2a–c which show interactive surface plot of effects of cohort specific allele enrichment on inverse variant weighted meta-analysis z-scores (IVW uplift) across MAFs (up to MAF 1%). Uplift is defined as the ratio of meta-analysed IVW Z-score to the Z-score of an individual study. (**a**) theoretically predicted IVW uplift. (**b**) observed IVW uplift. (**c**) Median absolute relative error (MARE, %) between simulated and theoretical IVW uplift values. For each combination of MAF and allelic enrichment, we simulated 1,000 datasets for two binary variables reflecting disease status. Study sample size and disease prevalence were fixed (matching values estimates from UKB and FG), genomic effects were randomly sampled from the set of positive effect sizes in UKB and FG (Supplementary Table 3), MAF was varied from 0.01% to 1% and allele enrichment (in the smaller study) ranged from 1 to 50. Tooltip is available at top right for additional interactive options including zooming, panning and saving as static images.

## Data Availability

Full summary association results of this study are accessible at 10.5281/zenodo.5571000. Summary and individual-level whole-exome sequencing data from UKB participants have been deposited with UKB and will be freely available to approved researchers via The UK Biobank Research Analysis Platform (https://www.ukbiobank.ac.uk/enable-your-research/research-analysis-platform). FG summary association results are being released bi-annually via https://www.finngen.fi/en/access_results and can be explored in a public results browser (https://r5.finngen.fi). All analyses in this manuscript which rely on variants that were directly interrogated through chip-based genotyping with the FG array rely on FG data freeze 5 (from 11 May 2021). Analyses in this manuscript that are based on imputed variants rely on FG data freeze 6, which is anticipated to become public in November 2021. Individual-level genotypes and register data from FG participants can be accessed by approved researchers via the Fingenious portal (https://site.fingenious.fi/en/) hosted by the Finnish Biobank Cooperative FinBB (https://finbb.fi/en/). Data release to FinBB is timed to the bi-annual public release of FG summary results which occurs twelve months after FG consortium members can start working with the data. Further datasets underlying this study have been derived from: Therapeutic Target Database (http://db.idrblab.net/ttd/); Phecode-ICD10 data (https://phewascatalog.org/phecodes_icd10); GWAS Catalog (https://www.ebi.ac.uk/gwas/); PhenoScanner (http://www.phenoscanner.medschl.cam.ac.uk/); ClinVar (https://www.ncbi.nlm.nih.gov/clinvar/); gnomAD (https://gnomad.broadinstitute.org/); Human Protein Atlas (https://www.proteinatlas.org/); and Ensembl (https://www.ensembl.org/index.html).

## Code availability

Custom analysis scripts used are available at https://github.com/cnfoley/Sun-et-al-2021-protein-coding-variants-in-human-disease.
