# Supplementary material for: Genetic associations of protein-coding variants in human disease
Source: Nature. 2022 Feb 23;603(7899):95–102. doi: 10.1038/s41586-022-04394-w (PMC8891017; doi:10.1038/s41586-022-04394-w)
Supplement: Supplementary file 1 — This file contains Supplementary Methods, results and discussions, Supplementary Figs. 1–6, full legends for Supplementary Tables 1–14, Biobank contributions to FinnGen, FinnGen ethics statement details, a list of FinnGen consortium contributors, PITX2 Function Study Group contributors, a list of Biogen Biobank Team contributors and Supplementary References. [file 41586_2022_4394_MOESM1_ESM.pdf]

nature portfolio

https://doi.org/10.1038/s41586-022-04394-w

Supplementary information

Genetic associations of protein-coding variants in human disease

In the format provided by the authors and unedited

Supplementary Information

Table of Contents

Table of Contents 1

Supplementary Methods: FinnGen genetic QC details 2

Supplementary Figure 1. Imputation info score distribution of FinnGen data 4

Supplementary information on choice of significance threshold 5

Supplementary Methods: Theoretical description and simulation of the impact of MAF enrichment on inverse-variance weighted (IVW) meta-analysis Z-scores 6

Supplementary Results: Simulations of MAF enrichment effect on inverse-variance weighted meta-analysis Z-scores 13

Supplementary Results: Summary of theoretical, simulation and observed results 16

Supplementary Figure 2. MAF enrichment on Z-scores 18

Supplementary Results: New roles for coagulation proteins in PE 19

Supplementary Results: *ADH1B* missense variant and alcohol related effects 20

Supplementary Results: Examples of coding associations in aiding drug development 21

Supplementary Results: METTL11B methylase missense variant in AF 22

Supplementary Discussion on coding associations in *SCN5A-SCN10A* and *HCN4-REC114* AF loci 23

Supplementary Methods: Functional characterization of PITX2c Pro41Ser 25

Supplementary Results on PITX2c Pro41Ser 28

Supplementary Discussion on PITX2c Pro41Ser 28

Supplementary Figures PITX2c-Pro41Ser 30

Supplementary File 1 Legend 34

Supplementary Files 2a-c Legend 35

Supplementary Table Legends 36

Biobank contributions to FinnGen 39

FinnGen ethics statement details 40

FinnGen consortium contributors 41

Biogen Biobank Team contributors 51

PITX2 Function Study Group contributors 52

Supplementary Information References 53

Supplementary Methods: FinnGen genetic QC details

Samples were genotyped with Illumina (Illumina Inc., San Diego, CA, USA) and Affymetrix arrays (Thermo Fisher Scientific, Santa Clara, CA, USA). Genotype calls were made with GenCall and zCall algorithms for Illumina and AxiomGT1 algorithm for Affymetrix data. Chip genotyping data produced with previous chip platforms and reference genome builds were lifted over to build version 38 (GRCh38/hg38) following the protocol described here: dx.doi.org/10.17504/protocols.io.nqtddwn.

Sample and initial variant QC

Individuals with ambiguous sex, high genotype missingness (>5%), excess heterozygosity (±4 SD) and non-Finnish ancestry were removed. In variant-wise quality control variants with high missingness (>2%), low Hardy-Weinberg equilibrium (HWE) p-value ($<1x10^{-6}$) and minor allele count, MAC<3 were removed. Chip genotyped samples were pre-phased with Eagle 2.3.5 (https://data.broadinstitute.org/alkesgroup/Eagle/) with the default parameters, except the number of conditioning haplotypes was set to 20,000.

High-coverage (25-30x) WGS data (N= 3,775) used to develop the SISu v3 reference panel were generated at the Broad Institute of MIT and Harvard and at the McDonnell Genome Institute at Washington University; and jointly processed at the Broad Institute. Variant callset was produced with GATK HaplotypeCaller algorithm by following GATK best-practices for variant calling.

Additional genotyping QC

Batchwise filters:

1) Not passing Axiom best practices and not recommended by Thermo Fisher Finngen team
2) Proportion of samples missing variant (F_MISS) > 0.02.
3) Hardy-Weinberg equilibrium test p-value ≤ 0.000001.
4) Genotype duplicates marker discrepancy > 2.
5) Mendelian discrepancy in known trios > 2.
6) Allele frequency differences to whole genome reference (SISu v3 imputation panel) and exome datasets (gnomAD exome version 2.1 (Finnish participants) and Finnish exons me collection) log2 fold change ±5 or allele frequency difference 0.1.

7) Variants showing significant differences to population reference panel in GWAS analysis (Sisu v3 imputation panel) p-value < dynamic p-value limit ($5x10^{-8}$/lambda3) (PLINK2, glm firth-fallback).

8) Common variants (AF > 0.05) that are not in reference panel WGS or exon data.

Batches were merged and variants were further filtered:

1) Variant call rate ≥ 80%

2) Hardy-Weinberg Equilibrium test p-value ≤ 0.000001

3) Variant call rate 97.8 % for variants that cannot be validated with allele frequency comparison to reference panel

4) Allele frequency differences to whole genome reference (SISu v3 imputation panel) and exome datasets (gnomAD exome 2.1 panel (Finnish participants) and Finnish exome collection) log2 fold change ±5 or allele frequency difference 0.1.

Imputation QC

Genotype-, sample- and variant-wise QC was applied in an iterative manner by using the Hail framework (https://github.com/hail-is/hail) v0.1 and the resulting high-quality WGS data for 3,775 individuals were phased with Eagle 2.3.5 as described above. Genotype imputation was carried out by using the population specific SISu v3 imputation reference panel with Beagle 4.1 (version 08Jun17.d8b, https://faculty.washington.edu/browning/beagle/b4_1.html) as described in the following protocol: dx.doi.org/10.17504/protocols.io.nmndc5e. Post-imputation quality-control involved non-reference concordance analyses, checking expected conformity of the imputation INFO-values distribution, MAF differences between the target dataset and the imputation reference panel and checking chromosomal continuity of the imputed genotype calls. The distributions of imputation INFO scores for all FinnGen variants and sentinel associated variants (Supplementary Table 3), stratified by MAF of 1% are shown in **Supplementary Figure 1 (Imputation info score distribution of FinnGen data).**

Supplementary Figure 1. Imputation info score distribution of FinnGen data

A) All FinnGen variants B) All FinnGen variants MAF < 1% C) All imputed variants shown in Supplementary Table 3 D) MAF <1% variants shown in Supplementary Table 3.

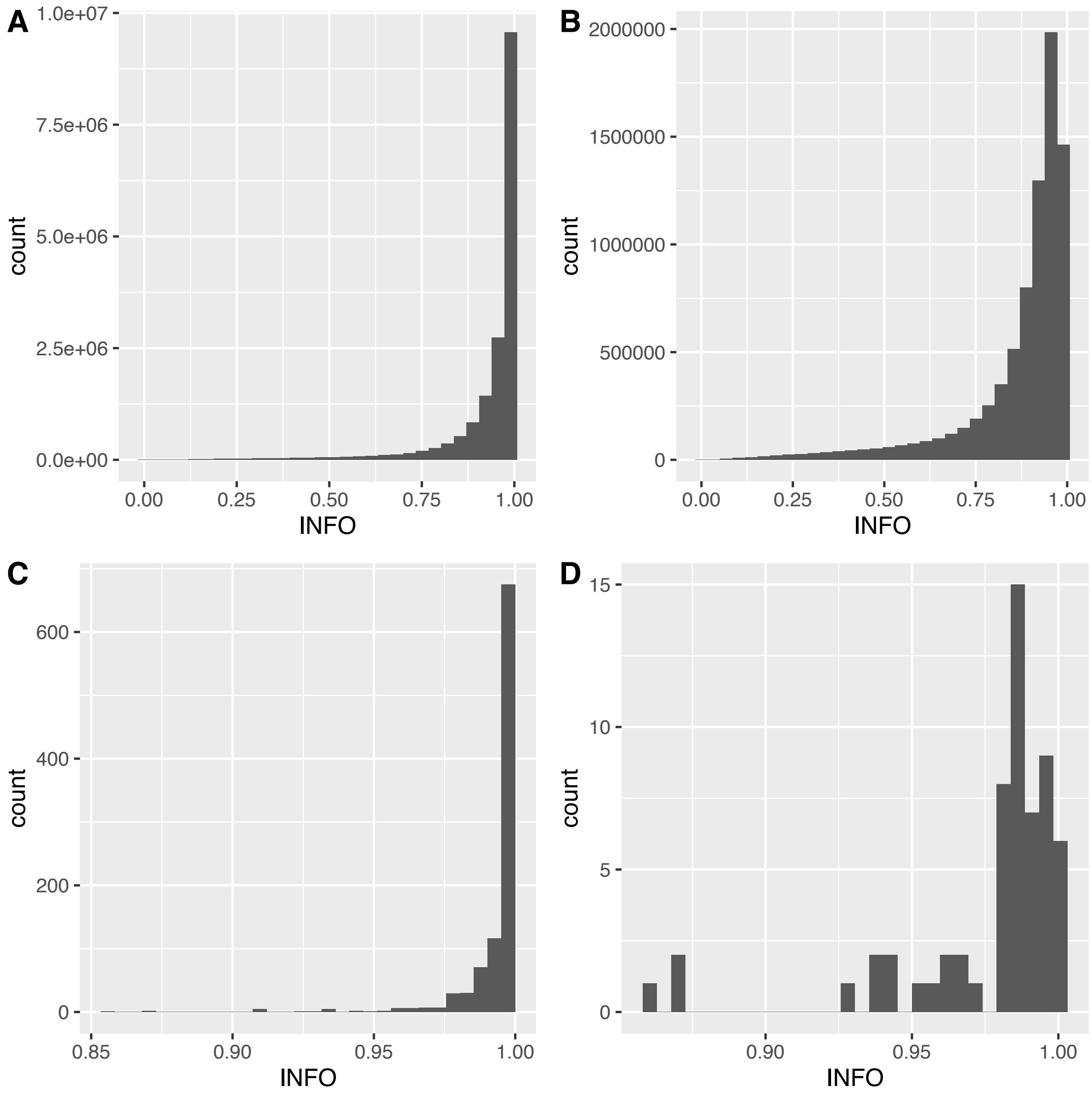

Supplementary information on choice of significance threshold

To define significance, we used a combination of (1) multiple testing corrected threshold of $p<2x10^{-9}$, $0.05/(\sim 26.8x10^{6})$ [sum (mean number of variants tested per disease cluster)], to account for the fact that some traits are highly correlated disease subtypes, (2) concordant direction of effect between UKB and FG associations, and (3) $p<0.05$ in both UKB and FG consistent with approaches in previous large-scale GWAS[51]. Here we assume all coding variants tested are independent, which is a stringent assumption as we ignore the LD, and we tested only the small subset of coding variants rather than the complete genome-wide. Our meta-analysis threshold should control for multiple testing whilst our added controls of concordant beta and $p<0.05$ should increase the robustness of the association across population cohorts without ignoring many potential true positives.

Our approach is also supported by empirical analyses in which we permuted the disease labels simultaneously across all diseases for each individual in the UKB samples to estimate (approximately) the number of expected associations under the null. The permutated sample data preserves the genetic LD and correlations between phenotypes. Then we ran the same type of genetic association analyses and subsequent meta-analyses, then used the same thresholds of concordant betas, $p<0.05$ in both cohorts, and meta-analysed $p<5x10^{-8}$, followed by clumping of the associations, in the same manner as our main analysis (**Methods)**. Under the null permuted scenario (permuted 10 times as sensitivity analyses without being too computationally cumbersome), we expected to see on average 39 associations (range: 32-47) compared to 975 associations that we actually observed, giving an approximate empirical false discovery rate (~39 expected false positives/all 975 positives observed) of ~0.04 (<0.05) on average (and 0.048 (47/975) in worst case scenario of 10 simulations). Thus, our choice of thresholds, even with a meta-analysis cut-off of $5x10^{-8}$, are also supported by empirical estimates in controlling for false positives.

Supplementary Methods: Theoretical description and simulation of the impact of MAF enrichment on inverse-variance weighted (IVW) meta-analysis Z-scores

Here we provide the details of theoretical frameworks and empirical simulations relating MAF enrichment and IVW meta-analysis Z-scores and the interpretation of the results.

Let the parameter b_i denote the effect of genotype g_i on disease endpoint Y_i in the ith study, for a total of $i = 1, 2, \ldots, m : m \geq 2$ studies. The meta-analysis inverse variance weighted Z-score, which aggregates information across all m studies, is given by

$$Z_{ivw} = \frac{\sum_{i=1}^{m} Z_i / \hat{\sigma}_{b_i}}{\sqrt{\sum_{i=1}^{m} 1 / \hat{\sigma}_{b_i}^2}},$$

where Z_i is the Z-score from the ith study, i.e.,

$$Z_i = \frac{\hat{b}_i}{\hat{\sigma}_{b_i}},$$

with $\hat{b}_i$ denoting the sample-based estimate of b_i and $\hat{\sigma}_{b_i}$ being its corresponding standard error. We re-write the above to illustrate the impact of integrating additional independent study information relative to a reference study, which we arbitrarily take to be study $i = 1$. That is, we write Z_{ivw} as:

$$Z_{ivw} = Z_1 \left(\frac{1 + \sum_{i=2}^{m} \frac{\sigma_1 Z_i}{\sigma_i Z_1}}{\sqrt{1 + \sum_{i=2}^{m} \sigma_1^2 / \sigma_i^2}} \right)$$

$$= Z_1 * \alpha$$

$$\Rightarrow \quad \alpha = \frac{Z_{ivw}}{Z_1}.$$

(1)

Hence, α denotes the increase/decrease in Z-score computed on aggregating results across m studies relative to the reference study. When $\alpha > 1$ the aggregated Z_{ivw} score is larger than the reference Z-score (Z_1) - a scenario we refer to as 'IVW uplift'. Our goal is to assess changes in α as a function of MAF and MAF-enrichment between $m = 2$ studies. To fix ideas, we are

interested in assessing uplift when (i) the reference study is the largest study and (ii) MAF is enriched in the smaller study, i.e., study 2. Hence, $N_1 \geq N_2$ and $MAF_2 \geq MAF_1$. The scenario in which the reference study has larger sample size and also enriched MAF, i.e., $N_1 \geq N_2$ and $MAF_1 \geq MAF_2$, is considered later.

Theoretical description of uplift α

In this section we present a formula which relates uplift α to the core parameters underpinning computation of the test statistic Z_{ivw}, i.e., MAF_i, disease prevalence (π_i) and sample size (N_i), for each of the $i \in \{1, 2\}$ studies. In doing so, we will (a) illustrate complexity in the relationship between α and the core parameters and (b) highlight the expected utility of a MAF-enriched study design, in particular how the probability of detecting novel (rare variant) associations increases as a function of enrichment.

Let the probability of disease status follow a logistic model, i.e.,

$$\pi_{y_i|g} = P(Y_i = 1 \mid g) = \frac{e^{(a_i + b_i g)}}{1 + e^{(a_i + b_i g)}},$$

(2)

where g denotes a genotype putatively associated with disease risk, a_i is the population baseline effect and b_i is the effect of genotype on disease risk. Given observed data $\{\boldsymbol{y}_i, \boldsymbol{g}_i\}$, estimates of the effect parameters, denoted $\{\hat{a}_i, \hat{b}_i\}$, are typically derived by maximizing the log-likelihood function

$$L(a_i, b_i) = \log P(\boldsymbol{y}_i \mid a_i, b_i, \boldsymbol{g}).$$

That is, values for $\{a_i, b_i\}$ which satisfy:

$$\frac{\partial L(a_i, b_i)}{\partial b_i} = \boldsymbol{g}^T \left(\boldsymbol{y} - \pi_{y_i|g}\right) = 0.$$

(3)

Assuming that the effect of genotype on disease risk is small, in the sense that $|b_i g| < 1$, it follows that

$$\pi_{y_i|g} = P(Y_i = 1 \mid g) = \frac{e^{(a_i + b_i g)}}{1 + e^{(a_i + b_i g)}}$$

$$= \frac{e^{a_i}}{1 + e^{a_i}} + \left(\frac{e^{a_i}}{(1 + e^{a_i})^2}\right) b_i g + \mathcal{O}\left({b_i}^2\right)$$

$$= a_i^* + b_i^* g + \mathcal{O}(b_i{}^2)$$

$$= \boldsymbol{\beta}_i \widetilde{\boldsymbol{g}} + \mathcal{O}(b_i{}^2),$$

(4)

where we have made the transformation of variables:

$$\boldsymbol{\beta}_i = (a_i^*, b_i^*),$$

$$\widetilde{\boldsymbol{g}} = (1, g)^T.$$

Hence, when $|b_i g| < 1$ the conditional probability of disease is well approximated by the linear predictor $\boldsymbol{\beta}_i \widetilde{\boldsymbol{g}}$. As it will be important later, the relationship between parameters in the logistic and the linear predictor is

$$b_i = \frac{b_i^*}{a_i^*(1 - a_i^*)} \quad \text{and} \quad a_i = \log\left(\frac{a_i^*}{1 - a_i^*}\right).$$

(5)

Combining equations (3) and (4), it follows that the score of the logistic model satisfies

$$\frac{\partial L(\boldsymbol{\beta}_i)}{\partial \boldsymbol{\beta}_i} = \widetilde{\boldsymbol{g}}_i{}^T\left(\boldsymbol{y}_i - \pi_{y_i|\widetilde{\boldsymbol{g}}_i}\right) \approx \widetilde{\boldsymbol{g}}_i{}^T(\boldsymbol{y}_i - \boldsymbol{\beta}_i \widetilde{\boldsymbol{g}}_i), \qquad |b_i g_{ij}| < 1, \quad j = 1,2, \dots, N_i,$$

which is approximately zero when

$$\widetilde{\boldsymbol{g}}_i{}^T(\boldsymbol{y}_i - \boldsymbol{\beta}_i \widetilde{\boldsymbol{g}}_i) = 0$$

$$\Rightarrow \ \widehat{\boldsymbol{\beta}}_i = \left(\widetilde{\boldsymbol{g}}_i{}^T \widetilde{\boldsymbol{g}}_i\right)^{-1} \widetilde{\boldsymbol{g}}_i{}^T \boldsymbol{y}_i,$$

(6)

note that these are the familiar ordinary least-squares estimates of main effect parameters in a linearized model. When the vector of genotypes has been centered, i.e., $\bar{\boldsymbol{g}} = 0$, it is straightforward to show that:

$$\hat{a}_i^* = \frac{\sum_{j=1}^{N_i} y_{ij}}{N_i} = \frac{N_i^*}{N_i} \quad \text{and} \quad \hat{\sigma}_{a_i}^* = \frac{\sqrt{\sum_{j=1}^{N_i} \pi_{y_{ij}|g_{ij}}\left(1 - \pi_{y_{ij}|g_{ij}}\right)}}{N_i},$$

$$\hat{b}_i^* = \frac{\sum_{j=1}^{N_i} y_{ij} g_{ij}}{\sum_{j=1}^{N_i} g_{ij}^2} \quad \text{and} \quad \hat{\sigma}_{b_i}^* = \frac{\sqrt{\sum_{j=1}^{N_i} \pi_{y_{ij}|g_{ij}}\left(1 - \pi_{y_{ij}|g_{ij}}\right) g_{ij}^2}}{\sum_{j=1}^{N_i} g_{ij}^2}.$$

(7)

We have used N_i^* to denote the number of cases in the ith study, thus $\hat{a}_i^*$ is a measure of population baseline disease prevalence. The linearized genetic effect in equation (4), however, can be interpreted as

$$\hat{b}_i^* = \frac{\sum_{j=1}^{N_i} y_{ij} g_j}{\sum_{j=1}^{N_i} g_j^2} = \frac{MAF_i^*}{MAF_i(1 - MAF_i)} \frac{N_i^*}{(N_i - 1)}, \qquad N_i \gg 1,$$

(8)

where MAF_i^* is an estimate of the MAF_i in the cases only:

$$MAF_i^* = \frac{1}{2N_i^*} \sum_{j=1}^{N_i} g_{ij} I(y_{ij} = 1) - MAF_i.$$

(9)

We will use equations (8) and (9) later, when aiming to interpret uplift via core model parameters.

Recall the relationship between the parameters in the logistic and linearized models presented in equation (5). After an application of the delta method, the variance of $\hat{b}_i$ in the logistic model (1) can be written as:

$$\hat{\sigma}_{\hat{b}_i}^2 = \operatorname{var}\left(\frac{\hat{b}_i^*}{\hat{a}_i^*(1 - \hat{a}_i^*)}\right) = \left|\frac{\hat{\sigma}_{b_i}^*}{\hat{a}_i^*(1 - \hat{a}_i^*)}\right|^2 \left(1 + \mathcal{O}\left(\frac{\hat{b}_i^*}{\hat{a}_i^*(1 - \hat{a}_i^*)} \frac{\hat{\sigma}_{a_i}^*}{\hat{\sigma}_{b_i}^*}\right)\right).$$

(10)

On combining equations (5) and (7), we reveal that the Z-score of the genetic effect in the logistic model (1) is well approximated by the estimated Z-score from the linearized effects (equation (7)), i.e.,

$$Z_i = \frac{\hat{b}_i}{\hat{\sigma}_{\hat{b}_i}} \approx \frac{\dfrac{\hat{b}_i^*}{\hat{a}_i^*(1 - \hat{a}_i^*)}}{\left|\dfrac{\hat{\sigma}_{b_i}^*}{\hat{a}_i^*(1 - \hat{a}_i^*)}\right|} = \frac{\hat{b}_i^*}{\hat{\sigma}_{b_i}^*} \operatorname{sgn}\left(\hat{a}_i^*(1 - \hat{a}_i^*)\right) = Z_i^*,$$

where we have used the fact that $\operatorname{sgn}\left(a_i^*(1 - a_i^*)\right) = 1$ from equation (7). Thus,

$$Z_i \approx Z_i^*$$

(11)

and it follows therefore, that the uplift α can be written as:

$$\alpha = \frac{1 + \dfrac{\hat{\sigma}_1 Z_2}{\hat{\sigma}_2 Z_1}}{\sqrt{1 + \hat{\sigma}_1^2 / \hat{\sigma}_2^2}}$$

$$\approx \frac{1 + \dfrac{\hat{a}_2^*(1-\hat{a}_2^*)\hat{\sigma}_{b_1}^* Z_2^*}{\hat{a}_1^*(1-\hat{a}_1^*)\hat{\sigma}_{b_2}^* Z_1^*}}{\sqrt{1 + \left(\dfrac{\hat{a}_2^*(1-\hat{a}_2^*)\hat{\sigma}_{b_1}^*}{\hat{a}_1^*(1-\hat{a}_1^*)\hat{\sigma}_{b_2}^*}\right)^2}}.$$

(12)

Explicit description of MAF enrichment, disease prevalence, sample size and log odds-ratio on IVW uplift (α)

In this section our goal is to gain some intuition as to how uplift can vary in terms of core parameters, e.g., MAF_i, MAF_i^*, N_i, N_i^*, for each study $i \in \{1,2\}$. We have demonstrated that equation (12) provides an accurate approximation to uplift (see **Extended Data Figures 5a-c, Supplementary Files 2a-c**), however these core parameters are implicitly defined in the approximation. To help provide some guidance on their explicit role, we replace $\hat{a}_i^*, \hat{\sigma}_{b_i}^*$ and Z_i^* in equation (12) with large sample (population) estimates, i.e., on assuming $N_i \gg 1$ for $i \in \{1,2\}$. In addition, we make the assumption of weak genetic effects (i.e., $|b_i| < 1$), low disease prevalence ($\pi_i \ll 1$) and rare/low MAF (in both studies), so that:

$$\hat{a}_i^*(1-\hat{a}_i^*) \approx \hat{a}_i^* \approx \pi_i \;:\quad N_i \gg 1 \text{ and } \pi_i \ll 1,$$

$$\pi_{y_{ij}|g_{ij}}\left(1-\pi_{y_{ij}|g_{ij}}\right) \approx \pi_{y_{ij}|g_{ij}} \;:\; \pi_{y_{ij}|g_{ij}} \ll 1,$$

$$\sum_{j=1}^{N_i} g_{ij}^2 \approx 2(N_i-1)MAF_i(1-MAF_i)$$

$$\approx 2MAF_i(N_i-1), \quad N_i \gg 1.$$

(13)

Recall that MAF_i^* denote the MAF in the cases, i.e.,

$$MAF_i^* = 0.5\sum_{j=1}^{N_i} I(y_{ij}=1)g_{ij} - MAF_i,$$

then, in combination with equations (4) and (7), it follows that

$$\pi_{y_{ij}|g_{ij}} \approx \pi_i\left(1 + \frac{MAF_i^*}{MAF_i}g_{ij}\right), \qquad N_i \gg 1 \text{ and } |b_i| < 1,$$

which, with equations (7), (12) and (13), returns:

$$\alpha \approx \frac{1+\kappa\frac{N_2}{N_1}\frac{MAF_2^*}{MAF_1^*}}{\sqrt{1+\kappa\frac{N_2}{N_1}\frac{MAF_2}{MAF_1}}}, \qquad N_i \gg 1 \text{ and } i \in \{1,2\},$$

(14)

where

$$\kappa = \frac{\pi_2}{\pi_1}\left(\frac{1+\frac{MAF_1^*}{MAF_1}}{1+\frac{MAF_2^*}{MAF_2}}\right).$$

(15)

Note that $X^* = \frac{MAF_2^*}{MAF_1^*}$ denotes the X^*-fold enrichment of MAF in the cases and $X = \frac{MAF_2}{MAF_1}$ the X-fold enrichment in the base-line population. Equation (14) makes clear that the relationship between the core parameters and uplift is complex. Nevertheless, there are some similarities between how power can increase with additional samples, via the ratio $\frac{N_2}{N_1}$, and increases in power due to X^*-fold and X-fold enrichment of MAF via the ratios $\frac{MAF_2^*}{MAF_1^*}$ and $\frac{MAF_2}{MAF_1}$ in (14). We explore this relationship further in section '**Analysis of Z-score boost due to MAF enrichment and increased sample size in simulated data**'.

To emphasize the role of effect sizes on uplift, recall that b_i in equation (2) is the 'log odds-ratio' (LOR_i) for the i-th study, i.e., $b_i = LOR_i$. By combining equation (5) with equations (7) and (8), it follows that

$$\widehat{LOR}_i = \hat{b}_i = \frac{\hat{b}_i^*}{\hat{a}_i^*(1-\hat{a}_i^*)} \approx \frac{\frac{MAF_i^*}{MAF_i(1-MAF_i)}\frac{N_i^*}{(N_i-1)}}{\frac{N_i^*}{N_i}\left(1-\frac{N_i^*}{N_i}\right)},$$

$$\Rightarrow \qquad LOR_i \approx \frac{MAF_i^*}{MAF_i}\frac{1}{(1-\pi_i)}, \qquad N_i \gg 1 \text{ and } i \in \{1,2\}.$$

(16)

From the above, the variable κ in (14) can be written as:

$$\kappa \approx \frac{\pi_2}{\pi_1}\left(\frac{1+LOR_1(1-\pi_1)}{1+LOR_2(1-\pi_2)}\right)$$

$$\approx \frac{\pi_2}{\pi_1}\left(\frac{1+LOR_1}{1+LOR_2}\right), \qquad \pi_i \ll 1 \text{ and } i \in \{1,2\},$$

which returns:

$$\alpha \approx \frac{1 + \frac{\pi_2}{\pi_1}\left(\frac{1 + LOR_1}{1 + LOR_2}\right)\frac{N_2}{N_1}\frac{MAF_2^*}{MAF_1^*}}{\sqrt{1 + \frac{\pi_2}{\pi_1}\left(\frac{1 + LOR_1}{1 + LOR_2}\right)\frac{N_2}{N_1}\frac{MAF_2}{MAF_1}}}, \qquad N_i \gg 1,\ \ \pi_i \ll 1 \ \text{ and } \ i \in \{1,2\}.$$

(17)

Equation (17) highlights the influence of disease prevalence (π_i), study sample size (N_i), MAF enrichment $\left(\frac{MAF_2^*}{MAF_1^*} \text{ and } \frac{MAF_2}{MAF_1}\right)$ and the log odds-ratio (LOR_i), i.e., regression effect size b_i, on IVW uplift α from studies $i \in \{1,2\}$. Owing to some dependencies between variables, e.g., equation (16) relates the log odds ratio to MAF and disease prevalence, it is difficult to make general statements about expected uplift based on increasing MAF enrichment, while attempting to fix the value of other variables. Broadly speaking, however, our theoretical, simulated and observed results indicate that IVW uplift α increases with increasing MAF-enrichment (**Extended Data Figures 5a-d, Supplementary Files 2a-c and Supplementary Figure 2 (MAF enrichment on Z-scores)**).

MAF enrichment in the larger study: $N_1 \geq N_2$ and $MAF_1 \geq MAF_2$.

From equation (14), if $MAF_1 \geq MAF_2$ and $MAF_1^* \geq MAF_2^*$ then both $X \leq 1$ and $\tilde{X} \leq 1$ and thus uplift decreases as a function of increased MAF enrichment in study 1.

Analysis of Z-score boost due to MAF enrichment and increased sample size in observed data

In an ideal scenario, one would also be able to compare the Z-score boost for enriched variants using an additional UKB like cohort matched for the sample size of FG which is not currently readily available for all diseases. However, we are able to subset the larger UKB cohort into a FG sample size (i.e., N=260405) matched cohort and use the remaining (N=132409) samples as a “base” analysis cohort and compare the Z-scores of base UKB meta-analysed with FG (UKBxFG) against base UKB meta-analysed with FG sample size matched UKB (UKBxUKB).

Focusing on associations with MAF<0.1 from **Supplementary Table 3**, we randomly subsetted UKB into a base UKB and a FG sample size matched UKB cohort (n=4 random subsets). We performed association testing and meta-analysis using the same approach and calculated the ratio of Z-scores ($Z_{UKBxFG}/Z_{UKBxUKB}$), log10 transformed so that >0 means Z-

score higher in UKBxFG than UKBxUKB, and <0 means Z-score higher in UKBxUKB than UKBxFG (**Extended Data Figure 5d**).

Analysis of Z-score boost due to MAF enrichment and increased sample size in simulated data

We further investigate the relative gain in IVW Z-scores across a broader range of enrichment values. For this, we modified our simulation strategy detailed in section **Simulations of MAF enrichment effect on inverse-variance weighted meta-analysis Z-scores** to match the cohort analyses above. Specifically, we followed the identical simulation protocol, yet now simulated results from a UKBxFG and separately UKBxUKB meta-analysis, followed by computing the ratio of IVW Z-scores. Results are presented in **Supplementary Figure 2 (MAF enrichment on Z-scores)**. Again, our theoretical predictions of the relative IVW uplift closely match the simulated results (**Supplementary Figure 2b**). For instance, when considering a 5-fold MAF enrichment in the FG study, our theoretically predicted estimates (equation (12)) from the simulation study closely approximate the observed estimates (**Supplementary Figure 2d and Extended Data Figure 5d)**. Our simulations further support our findings that studies involving cohorts with MAF enriched designs are likely to provide (potentially significant) additional power gains relative to gains achieved by increasing sample size alone.

Supplementary Results: Simulations of MAF enrichment effect on inverse-variance weighted meta-analysis Z-scores

To go some way toward assessing the impact of MAF-enrichment for rare variants (MAF<1%) on IVW uplift in a realistic setting, we performed a simulation study informed by UKB and FG study information. We simulated two binary variables, representing two disease endpoints, with study sample sizes set to $N_1 = 392814$ and $N_2 = 260405$, respectively. To specify disease prevalence parameters, we computed the median ratio of disease prevalence across all $l = 1,2,\ldots,744$ disease endpoints in UKB and FG datasets, i.e., $\text{median}\left({}^{\pi_{2l}}/_{\pi_{1l}}\right)$ (**Supplementary Table 3**). As $\text{median}\left({}^{\pi_{2l}}/_{\pi_{1l}}\right) = 1.5$, we set $\pi_2 = 1.5\pi_1$ and fixed $\pi_1 = 0.005$, which is approximately 2 times the median disease prevalence across the 744 disease endpoints within UKB. To assess impact of MAF enrichment across a range of rare/low MAFs, we varied MAF in the reference study, $MAF_1 \in \{10^{-4}, 5 \times 10^{-4}, 10^{-3}, 2.5 \times 10^{-3}, 5 \times 10^{-3}, 0.01\}$ and X-fold MAF-enrichment in study 2, $X \in \{1, 5, 10, 20, 30, 50\}$. Note that

$MAF_1 = 0.01$ and $X = 50$ results in $MAF_2 = 0.5$, at which point the effect allele switches in study 2. This motivated our choice of maximum MAF and X-fold enrichment values.

Data for disease status were generated under a logistic model, i.e., equation (2):

$$logit\left(\pi_{y_i|g_{ij}}\right) = a_i + b_i * g_{ij},$$

$$a_i \approx logit(\pi_i),$$

where

$$\pi_{y_i|g_{ij}} = P(Y_i = 1 \mid g_{ij}), \qquad i \in \{1,2\}, j = 1,2, \dots, N_i,$$

denotes the probability of disease conditional on genotype g_{ij} for the jth participant in the ith study. Finally, a value for b_i - the effect of genotype on disease – was randomly sampled from the set of positive regression coefficients (to reflect the fact that vast majority of low frequency variants have disease risk-increasing effects), computed in UKB (for b_1 values) and FG (for b_2 values) (**Supplementary Table 3).**

We validate the accuracy of the theoretical approximation to the IVW uplift, i.e., equation (12), by monitoring the median absolute relative error (MARE) across $i = 1,2, \dots, 1000$ simulated datasets and over variety of MAF and enrichment values. Specifically, we computed

$$MARE_{jk} = \text{median}\left|\Omega_{jk}\right|, \quad \Omega_{jk} = \left\{\omega_{1jk}, \omega_{2jk}, \dots, \omega_{1000jk}\right\},$$

where

$$\omega_{ijk} = \left\{\frac{\alpha_{obs_{ijk}} - \alpha_{\exp_{ijk}}}{\alpha_{obs_{ijk}}}\right\}$$

with j indexing the set of values $MAF_1 \in \{10^{-4}, 5 \times 10^{-4}, 10^{-3}, 5 \times 10^{-3}, 0.01\}$ and k indexing the set of X-fold enrichment values $X \in \{1, 5, 10, 20, 30, 50\}$, i.e., $X = \frac{MAF_2}{MAF_1}$. Our findings reveal very good correspondence between the theoretical prediction (equation (12)) and simulated uplift α-values (**Extended Data Figure 5c**; **Supplementary File 2c**). Excluding the scenarios in which $MAF_1 = 10^{-4}$, the MARE (as a percentage) was $\lesssim 5$ % across the range of MAF and enrichment values considered: highlighting the accuracy of the approximation in equation (12) over a wide range or rare MAFs and MAF-enrichment values. The observed increase in MARE when $j = 10^{-4}$ is a consequence of the approximation equation (10) becoming less accurate – this happens when $N_1^* MAF_1 \lesssim 1$ and hence the number of cases with an effect allele in the reference sample is typically very small. **Extended Data Figures 5a-c** and **(Supplementary Files 2a-c)** makes clear that, on approaching and passing

this boundary (which in our simulation set-up occurs when $MAF_1 \approx 5 \times 10^{-4}$) the MARE quickly increases. Despite this, in our simulation the MARE$\lesssim 20\%$ at $MAF_1 = 10^{-4}$.

Supplementary Results: Summary of theoretical, simulation and observed results

We observed the uplift to increase with increasing MAF and allelic enrichment as expected. The gain in IVW uplift is not linear across MAFs, with most of the gain in uplift occurring at MAF<0.5% before plateauing (**Extended Data Figure 5a; Supplementary File 2a**). There is a rapid increase in uplift as baseline MAF increases from 10^{-4} to 10^{-3} suggesting the added potential for novel findings benefitting very rare allele frequency ranges. The increase in utility at rare MAF ranges has practical impact on novel association findings as common and low frequency variant association can be well-powered to be detected already at biobank sizes for prevalent diseases. IVW uplift also increases with allelic enrichment, once again sharp increases in uplift are expected and observed at lower enrichment values, eventually flattening out towards MAF 1% and fold enrichment 50x (**Extended Data Figure 5a-c, Supplementary Files 2a-c**). Note that, MAF ~1% and fold enrichment ~50x would fall within the region of inflection (top right quadrants of **Extended Data Figure 5a and 5b**) where the MAF in the enriched cohort approaches 50%. In practice, the vast majority of rare variants do not fall near the region of inflection as it would suggest, for example, a MAF of 1% in one study and near 50% in the other. Our results make clear that the surface of IVW uplift values using observed data is in general similar to theoretical results.

For the observed sentinel associations (MAF<0.1, **Supplementary Table 3)**, we compared the Z-score ratio of a randomly subsetted "base" UKB cohort (of N=132409 samples) meta-analysed with FG (UKBxFG) against the base UKB meta-analysed with the remaining N=260405 UKB samples (UKBxUKB), to estimate the observed gains in power accounting for sample size (**Extended Data Figure 5d)**. We repeated the process of randomly subsetting the UKB samples a total of n=4 times and aggregated the results - to assess sensitivity to our choice of "base" UKB samples (and equivalently the remaining UKB samples). Note that, a value of n=4 was chosen as the association analyses for each subset is computational cumbersome. Consistent with our theoretical and simulation expectations, we found a clear trend between allelic enrichment and Z-score gains (**Extended Data Figure 5d)**, where higher variant fold enrichment in FG and UKB, on average, led to more significant associations in UKBxFG and UKBxUKB respectively.

Taken together, the simulation results both theoretically and empirically along with observed data show the added utility in boosting association findings through cohort specific allelic enrichment additional to increased power from larger sample size.

Supplementary Figure 2. MAF enrichment on Z-scores

Surface plot illustrating the relative gain in inverse variant weighted meta-analysis z-score (IVW uplift) between MAF enriched meta-analysis and standard (i.e., additional samples) meta-analysis. For each combination of MAF and allelic enrichment, we simulated 1,000 datasets for three binary variables, representing disease status in: (1) a baseline cohort of N=132,409 individuals (simulated using UKB study characteristics and denoted "UKB_1"); (2) a second cohort of N=260,405 individuals (simulated using UKB study characteristics and denoted "UKB_2"); and (3) a third cohort of N=260,405 individuals (simulated using FG study characteristics, denoted "FG"). Disease prevalences were fixed (matching values estimated from UKB and FG data), genomic effects were randomly sampled from the set of positive effect sizes in UKB and FG **(Supplementary Table 3)**, MAF was varied from 0.01% to 1% and allele enrichment (in study-3 only) ranged from 1 to 50. Relative gain is defined as the ratio of MAF enriched to no MAF enrichment IVW Z-scores, i.e, $\frac{Z_{UKB_1 \times FG}}{Z_{UKB_1 \times UKB_2}}$. **(a) theoretically predicted relative IVW uplift. (b) observed relative IVW uplift. (c) Median absolute relative error (MARE, %) between simulated and theoretical relative IVW uplift values. (d) first, second (median) and third quartiles of the distribution of relative IVW uplift when study 3 has 5-fold MAF enrichment - for reference, the distribution of the IVW uplift for studies 1 and 2, i.e., "UKB_1" and "UKB_2", is presented.**

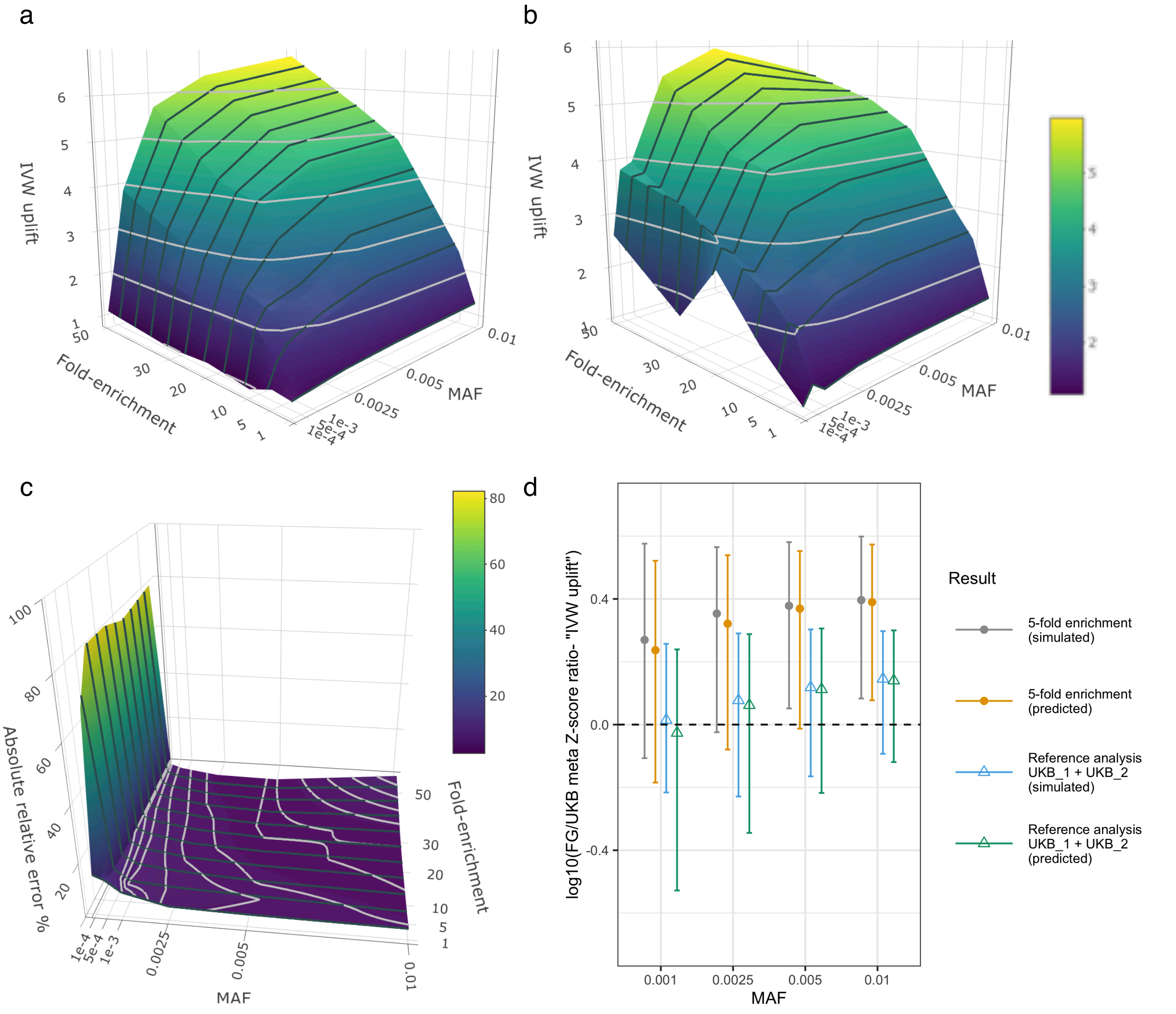

Supplementary Results: New roles for coagulation proteins in PE

We discovered a rare missense mutation in F10, enriched by ~5-fold in FG (rs61753266:A; Glu142Lys; MAF=0.33% [UKB], 1.85% [FG]), to be protective against PE (log[OR]=-0.44, p=2.9x10-9). This variant has been associated with reduced plasma coagulation factor X (beta=-1.12, p=2.0x10^{-8}) and factor Xa (beta=-1.54, p=7.9x10^{-15}) levels previously[51], as well as clinical factor X deficiency[52]. Deficiencies in coagulation factors, including factor X, are associated with increased bleeding liability and reduced thrombotic risk. In a similar fashion, we found a previously reported venous thromboembolism risk-reducing variant (rs4525:C; His865Arg; MAF=27.2% [UKB], 22.3% [FG]) in *F5* that is also protective for PE (log[OR]=-0.14, p=1.2x10^{-15}) and associated with reduced plasma F5 levels[53] (beta=-0.25, p=6.0x10^{-7}). This variant acts opposite to the well-established risk promoting F5 Leiden missense mutation, which leads to increased resistance to activated protein C cleavage[54] and thromboembolism liability, thus unravelling that coding variants in *F5* can have opposite effects on PE risk at the population level (**Extended Data Figure 7b)**.

We also found a rare variant in fibrinogen (*FGB* rs2227434:T; Pro100Ser; MAF=0.13% [UKB], 0.15% [FG], **Extended Data Figure 7a, Supplementary Table 3**) that associated with increased PE risk at nominal GWAS significance (log[OR]=1.03, p=1.5x10^{-8}). Missense mutations in *FGB* have previously been linked to both elevated and reduced fibrinogen levels through GWAS[55,56], as well as congenital afibrinogenemia[57].

We performed Mendelian randomisation (MR) using rs4525 and rs61753266 as instruments to estimate the relative reduction in PE risk due to reduced F5 ($beta_{MR}$=0.57, p=1.0x10^{-15}) and F10 levels (F10: $beta_{MR}$=0.40, p=2.9x10^{-9}; F10a: $beta_{MR}$=0.28, p=2.9x10^{-9}) respectively (**Extended Data Figure 7b)**. MR results support the expected clinical indication of factor X inhibitors in thromboembolic diseases and the hypothesis that developing drugs inhibiting factor V will also likely be beneficial for PE.

Supplementary Results: *ADH1B* missense variant and alcohol related effects

The low-frequency missense variant in *ADH1B* (rs1229984:T; Arg48His; MAF=2.2% [UKB], 0.5% [FG]) that is associated with increased enzymatic activity of alcohol dehydrogenase and reduced alcohol tolerance, is also associated with reduced risk of alcohol-related disorders (alcoholic liver disease: log[OR]=-1.08, p=1.5x10^{-9}; mental and behavioural disorders due to alcohol: log[OR]=-0.82, p=1.2x10^{-33}) and increased risk of gout (log[OR]=0.39, p=3.3x10^{-10}). Notably, the alcohol dependence disorder-promoting *ADH1B* allele (C) is also associated with reduced IGF-1 (beta=-0.11, p=1.5x10^{-51}) and vitamin D levels (beta=-0.049, p=2.6x10^{-10}), increased levels of liver enzymes (alkaline phosphatase: beta=0.087, p=3.1x10^{-37}; gamma-glutamyl transferase: beta=0.041, p=1.62x10^{-9}) and total bilirubin (beta=0.031, p=5.1x10^{-7}), macrocytosis with increased mean corpuscular volume (beta=0.047, p=4.2x10^{-12}) and mean corpuscular haemoglobin (beta=0.048, p=1.4x10^{-12}), as well as reduced erythrocyte count (beta=-0.034, p=3.2x10^{-8}). The gout risk reducing C allele is associated with reduced urate levels (beta=-0.061, p=1.4x10^{-21}).

Supplementary Results: Examples of coding associations in aiding drug development

In addition to providing further support for well-established drug target associations such as between *PCSK9* loss-of-function and hypercholesterolaemia, or *F10* loss-of-function and venous thromboembolism, we also found an association between a common missense variant (rs231775:G) in *CTLA4* with increased risk of thyrotoxicosis (log[OR]=0.12, p=8.5x10^{-13}). Since this variant is also a blood eQTL for decreased *CTLA4* expression[58] (Z-score=-6.91, p=5.0x10^{-12}), the association between genetic CTLA4 reduction and thyroid dysfunction might contribute to the adverse event of hyperthyroidism in cancer patients treated with CTLA4 inhibitors[59].

Genetics can inform drug discovery also on alternative indications for repurposing. For example, *TYK2* inhibitors are being tested in clinical trials for various autoimmune and psoriatic diseases[60]. Consistent with previous GWAS[61], we found a missense variant in *TYK2* (rs34536443:C) to be associated with reduced risk of rheumatoid arthritis and psoriatic diseases (**Supplementary Table 3**). Our analyses establish this variant to also be associated with sarcoidosis (log[OR]=-0.41, p=3.6x10^{-8}), proposing sarcoidosis as a new indication for *TYK2* inhibitors. Similarly, while the pleiotropy of *CHEK2* provides support for exploring CHEK2 inhibitors against a broader spectrum of malignancies, our analyses also highlight a risk for potential haematological perturbations upon CHEK2 inhibitor treatment.

Supplementary Results: METTL11B methylase missense variant in AF

METTL11B is a N-terminal monomethylase that methylates target proteins containing a N-terminal [Ala/Pro/Ser]-Pro-Lys motif[62]. The missense variant Ile127Met (SIFT=0, PolyPhen=1.0) falls within a conserved motif in the enzyme's S-adenosylmethionine/S-adenosyl-l-homocysteine ligand binding site[63]. *METTL11B* expression is enriched in heart and skeletal muscles with highest expression in heart, in particular cardiomyocytes[64,65]. We scanned protein sequences for a presence of the [Ala/Pro/Ser]-Pro-Lys motif and elevated expression in cardiomyocytes (**Methods**, **Supplementary Table 12**). We found statistically significant enrichment of genes encoding [Ala/Pro/Ser]-Pro-Lys motif containing proteins amongst genes with elevated expression in cardiomyocytes (OR=1.34, 95% CI=[1.16, 1.54], p=3.2x10^{-5}), many of which show N-terminal [Ala/Pro/Ser]-Pro-Lys motifs (OR=1.24, 95% CI=[1.06, 1.44], p=5.6x10^{-3}). The group of proteins containing [Ala/Pro/Ser]-Pro-Lys motifs includes several well-established AF genes[66] such as potassium channels (*KCNA5, KCNE4, KCNN3*), sodium channels (*SCN5A, SCN10A*), *NPPA,* and *TTN*. Our data support *METTL11B* as the causal gene in this GWAS locus and a relevance for N-terminal [Ala/Pro/Ser]-Pro-Lys methylation in cardiomyocytes for AF.

Supplementary Discussion on coding associations in *SCN5A-SCN10A* and *HCN4-REC114* AF loci

The SCN10A Ala1073Val variant associated with AF

The *SCN5A-SCN10A* locus is well known to be associated with cardiac arrhythmic disorders including AF. A common variant in *SCN10A* alternatively presents Alanine or Valine at the p.1073 position and is associated with AF. In the present study, the Val1073 variant was associated with a decreased risk of developing of AF. This is consistent with the previously shown association of the Ala1073 variant with higher incidence of AF[67]. The Val variant was also associated with slower cardiac conduction[68]. Functional studies of the Nav1.8 sodium channel encoded by *SCN10A* have shown the Val variant to exhibit lower current density and lower persistent (or late) current than the Ala form[67,69] and faster inactivation and slower and incomplete recovery from inactivation[69]. There is a negligible transcription of Nav1.8 in the human atrial in sinus rhythm and AF subjects[70] but a sizeable expression of Nav1.8 channels in cardiac ganglionated plexi[71,72] localized within the atrial epicardial adipose tissue. Perfusion of these plexi with a specific Nav1.8 inhibitor prevented the ability of vagus nerve stimulation to decrease heart rate, to increase the PR interval and to increase the inducibility of AF by local atrial tachypacing[71]. Of note, the Val1073 variant was also linked to a diminution of mechanical pain sensation[73].

The *SCN5A* Thr220Ile variant associated with AF

The *SCN5A* Thr220Ile variant causes a 5 mV hyperpolarizing shift of the SS-inactivation and a 25% reduction of current density of the Nav1.5 channel[74,75]. The Thr220Ile variant lies in the S4, voltage sensing segment, of the first transmembrane domain DI of Nav1.5[76] and causes a loss of function (LOF) through a 5 mV hyperpolarizing shift of steady-state inactivation and a 25% lower current density[74,75]. This variant has also been associated with conduction defects, sick sinus syndrome and atrial standstill[77] in family studies with bradycardic changes. Importantly, this variant has also been associated with dilated cardiomyopathy (DCM)[78]. The Thr residue implicated is neighbored, in the same DI/S4 voltage sensor domain, by three Arg residues whose mutations Arg219His, Arg222Gln, and Arg225Trp all are also associated with DCM. They share a substitution of the positively charged Arg residue by non-polar ones, and this creates a cation-selective gating pore current that carries a permanent depolarizing current and disturbs ionic homeostasis (reviewed in [79,80]). The moderate LOF found with Thr220Ile is

not likely to explain such a severe phenotype as DCM. An alternative hypothesis is that replacement of the polar Thr by the non-polar Ile might create a cation-selective gating pore[79,80].

The *HCN4* Asp364Asn variant associated with AF

Another AF GWAS locus is tagged by the common intergenic sentinel variant rs74022964 between HCN4 and REC11432,33. We identified a rare, FG enriched variant in HCN4 (rs151004999:T; Asp364Asn; MAF=0.045% [UKB], 0.17% [FG]; SIFT=0.05, PolyPhen=0.41) as associated with increased AF risk (log[OR]=0.72, p=2.8x10-8). HCN4 is a hyperpolarization-activated ion channel contributing to the cardiac pacemaker current known as the funny current (I_f). Mutations in *HCN4* have been associated with familial bradycardia (also known as sick sinus syndrome 2) and Brugada syndrome 8 in family studies[81]. Consistently, in addition to the AF signal, we also found an association with decreased heart rate (beta=-0.49, p=3.8x10^{-21}).The Asp364Asn variant lies in a poorly conserved region of the protein and its effects on the HCN4 channel have not been explored (ClinVar). The association with decreased heart rate and increased AF risk potentially points to a LOF effect of HCN4, although further functional studies are required to elucidate this further.

Supplementary Methods: Functional characterization of PITX2c Pro41Ser

Methods relating to **Independent AF participants, DNA extraction, PCR and Sanger sequencing** relating to the analysis of association of Pro41Ser PITX2 with AF in a French cohort were previously described in[82].

PITX2c cDNA constructs

For gene reporter essay in TM-1 cells (Transformed human trabecular meshwork cells), the Pro41Ser variant was generated by site-directed mutagenesis (QuikChange®, Agilent Technologies, Mississauga, ON) using a pcDNA4/HisMax© plasmid (Invitrogen, Burlington, ON) expressing the wild-type Xpress™-tagged *PITX2c*[83] (accession number for the PITX2 sequence is MIM 601542). The forward mutagenesis primers are similar to those reported before[82]. NdeI/XhoI restricted fragments were subcloned into pcDNA4:*PITX2c*. The plasmids were purified using QIAGEN Maxiprep or QIAprep® Spin Miniprep kits (QIAGEN Inc., Mississauga, ON) and validated by sequencing. For HL-1 cells experiments, we generated a V5-Tag-pcDNA3.1/Zeo vector carrying the open reading frames from human *PITX2* wild type and mutant sequences as previously described[82].

Western blot Analysis

Protein expression of the Pro41Ser variant was assessed in TM-1 cells using Anti-Xpress™ (Invitrogen) antibodies to detect Xpress-tagged recombinant proteins[84]. The PITX2c protein expression was also confirmed in HL-1 cells (mouse immortalized atrial myocardial cells)[85], transfected with V5-tagged vectors. The cells were seeded in 24-well plates and transfected using lipofectamine 2000 (Invitrogen) and 0.2µg of plasmid per well (V5-PITX2c WT or V5-PITX2c Pro41Ser). 24hr after transfection, cells were collected, and Western blot was performed as previously described[82].

Transactivation assays

A pGL3Basic-SLC13A3 reporter plasmid was engineered to contain an upstream promoter fragment of SLC13A3 and contains a putative binding site for PITX2c[83]. The promoter controls the transcription of an appended luciferase-coding sequence.

A PITX2c-expressing plasmid or the empty expression vector were co-transfected (n=3) with the pGL3Basic-SLC13A3 reporter and a pCMVβ transfection control vector (Clontech

Laboratories, Inc, Mountain View, CA) into TM-1 cells (500 ng, 60 ng and 30 ng respectively). Luciferase activity was measured by luminometry (Turner Designs, Sunnyvale, CA) according to the manufacturer's instructions and standardized to the β-galactosidase (internal control, β-galactosidase Enzyme Assay System, Promega, Madison, WI, USA).

Immunolocalization

Immunofluorescence was carried out as previously described[86]. 24hr after transfection, TM-1 cells (grown on coverslips) were fixed with 2% paraformaldehyde/PBS for 20 min. Antibodies: anti-Xpress and Mouse-Cy3 (Jackson ImmunoResearch, West Grove, PA) were used at a dilution of 1:500. The nuclei were labelled with a mounting medium containing DAPI. No significant difference of PITX2c protein expression was seen when normalized to GADPH internal control.

Electrophoretic mobility shift assays

WT and mutated PITX2c proteins' affinity with the DNA bicoid binding site were investigated by non-denaturing polyacrylamide gel electrophoresis of a mixture of double-stranded Cy3-labelled DNA (at 0.5 µM) and whole-cell extracts of TM-1 cells (40 µg) transfected with a *PITX2c* construct.

Quantitative Reverse Transcriptase–PCR Analyses in HL-1 cells

HL-1 cells transfected with V5-tagged vectors were collected 24h after transfection. Total RNA extraction was carried out using SV Total RNA isolation system (Promega) followed by cDNA synthesis using Maxima first strand cDNA synthesis kit for RT-qPCR (Fermentas life science) according to the manufacturers' instructions. qRT-PCR was performed using SsoFast™ EvaGreen (BioRad) supermix in a CFX384 QPCR System (BioRad).

Additional details on antibodies and cell-lines

Antibodies used: For Western blots on HL-1 cell protein extracts, antibodies were a mouse monoclonal anti-V5 (V8012, Sigma-Aldrich) and a mouse monoclonal anti-GAPDH (G8795, Sigma-Aldrich). Batch numbers unknown. Antibodies used with TM-1 cells experiments: Anti-Xpress Monoclonal Antibody was purchased from ThermoFisher Scientific (#R910-25). Batch number unknown. Goat Anti-Mouse-IgG coupled to Cyanine3: Cy™3 AffiniPure Goat Anti-Mouse IgG (H+L) polyclonal, was purchased from Jackson ImmunoResearch Laboratories Inc. (#115-165-003). Batch number unknown. (115-165-003).

Antibody validation:

V8012, Sigma-Aldrich

https://www.sigmaaldrich.com/FR/fr/search/v8012?focus=products&page=1&perPage=30&sort=relevance&term=V8012&type=product

V8012 V5-10, monoclonal WB, ICC

G8795, Sigma-Aldrich

https://www.sigmaaldrich.com/FR/fr/search/g8795?focus=products&page=1&perPage=30&sort=relevance&term=G8795&type=product

G8795 GAPDH-71.1, monoclonal WB, ARR, ICC, ELISA mouse, mink, rabbit, rat, human, hamster, canine, turkey, chicken, monkey, bovine. Sigma-Aldrich quote papers and show images from the literature in which Abs V8012 and G8795 were used, but do not endorse them as a validation.

Cell lines: The TM-1 cell line are also named HTM or HTMC (immortalized Human Trabecular Meshwork cells). The TM-1 cell line (immortalized human Trabecular Meshwork cells) was a gift from Dr. Vincent Raymond, and was cultured in Dulbecco′s Modified Eagle′s Medium - low glucose (Sigma, #D6046). HL-1 cell were a gift from Dr. W.C. Claycomb and were cultured using his dedicated "Claycomb Medium" ordered from Sigma-Aldrich (product 51800C) and the Fetal Calf Serum lot certified by Dr. Claycomb, ordered from Sigma-Aldrich (product F2442 lot #058K8426). Since Dr. W.C. Claycomb passed-away the HL-1 cells are provided by SIGMA and other firms. HL-1 cells were authenticated by Dr. W.C. Claycomb. No authentication tests have been performed in our laboratories. The ISLAC register searches for TM-1 or HTM or HTMC and for HL-1 did not report any match.

All experimental quantitative data were analyzed using unpaired two-tailed Student's t-test. Unless otherwise mentioned, assays and measurements were done in triplicate. Data are reported as mean ± SEM.

Supplementary Results on PITX2c Pro41Ser

We genotyped the *PITX2* gene in a cohort of 60 unrelated patients with a history of atrial fibrillation and 389 controls. We identified a novel heterozygous variant, a single-base substitution of a cytosine by a thymidine at nucleotide 121 (c.121C>T) resulting in an amino acid change from proline to serine at position 41 (Pro41Ser) (**Supplementary Figure 3**). The propositus is a 65-years old female. She was first diagnosed with paroxysmal AF without heart disease when she was 48 years old.

Reporter gene assay

We verified that the increased transactivation effects did not result from a higher protein expression of the variant (see Western blot images in **Supplementary Figure 4**) or from a change in the intracellular distribution of the recombinant proteins since the nuclear localization was similar to that of the WT (**Supplementary Figure 5a**). Moreover, they were not linked to the ability of WT and mutated PITX2c proteins to interact with the DNA bicoid binding site as tested by EMSA assay (**Supplementary Figure 5b**). The full images of the EMSA gel and corresponding Western blot are shown in **Supplementary Figure 6**.

qRT-PCR determination of connexins and ion channels

Control experiments show the differences in expression of PITX2c (**Supplementary Table 14**) that were taken into account to normalize the mRNA level measurements. Further, it was verified that cells transfected with an empty vector had mRNA levels similar to those in non-transfected cells (**Supplementary Table 14**).

Supplementary Discussion on PITX2c Pro41Ser

PITX2c Pro41Ser is the second PITX2c gain-of-function mutation identified as underlying AF and appears to impact PITX2c in a similar manner as a previously described Met207Val mutation that had been identified in a family study[82]. The Pro41Ser variant is situated in exon 4, which encodes a 52 amino-acid N-terminal sequence specific to PITX2c and humans. This domain has been ascribed a crucial role in cardiac asymmetric morphogenesis[87]. Its overexpression on the left side of the chick embryo randomized the direction of heart looping, an effect that was lost when a Lysine at amino acid position 41 (amino acid position 33 in human PITX2c) was artificially mutated to an Arginine[88].

We observed a gain-of-function in the transactivation activity of Pro41Ser-PITX2c (**Extended Data Figure 8**). PITX2c has been found to be over-expressed in human atrial myocytes isolated from patients with chronic atrial fibrillation[89]. The Pro41Ser mutant in HL-1 cells increased KCNH2 mRNA 1.81-fold. Such an increase in the rapid outward rectifier current I_{Kr} (encoded by KCNH2), likely shortens atrial action potential duration and refractory period, which favors re-entry of excitation and thus AF[90]. Such I_{Kr} gain-of-function induced by mutations of KCNH2 has been associated with familial AF[91,92]. In HL-1 cells, the Pro41Ser mutant caused an increase in Cx40 and Cx43 mRNAs by1.76-fold and 1.85-fold respectively (**Supplementary Table 14**). Such a parallel increase was found in Cx40 and Cx43 proteins in the left atrial myocardium of patients with AF[93]. In summary, the results obtained for Pro41Ser PITX2c experimentally are likely to explain the association of this variant with increased risk of AF.

Supplementary Figures PITX2c-Pro41Ser

Supplementary Figure 3. Electropherogram of the proband carrying the Pro41Ser variant. Arrows show the cytosine to thymidine change and box shows the resulting codon change.

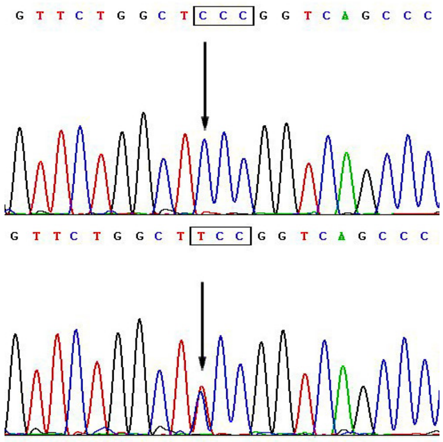

Supplementary Figure 4. Western Blots related to PITX2c transactivation reporter assays of Extended Data Figure 8. (a) Western blot of PITX2c proteins in TM-1 cells: the WT and Pro41Ser PITX2c proteins were expressed comparably in TM-1 cells. Molecular weight markers were cropped from a visible light image of lane 1. The numbers on the left are the molecular weights in kDa. (**b**) Superimposed labeling of PITX2c and the α-tubulin loading control. Rectangles in panel b delineate the regions that were cropped to provide panel c. "empty" is empty vector transfection; P41S stands for Pro41Ser, NR: unrelated to the present study. (**c**) Cropped data. EV is empty vector. These data are representative of n=3 separate transfection experiments.

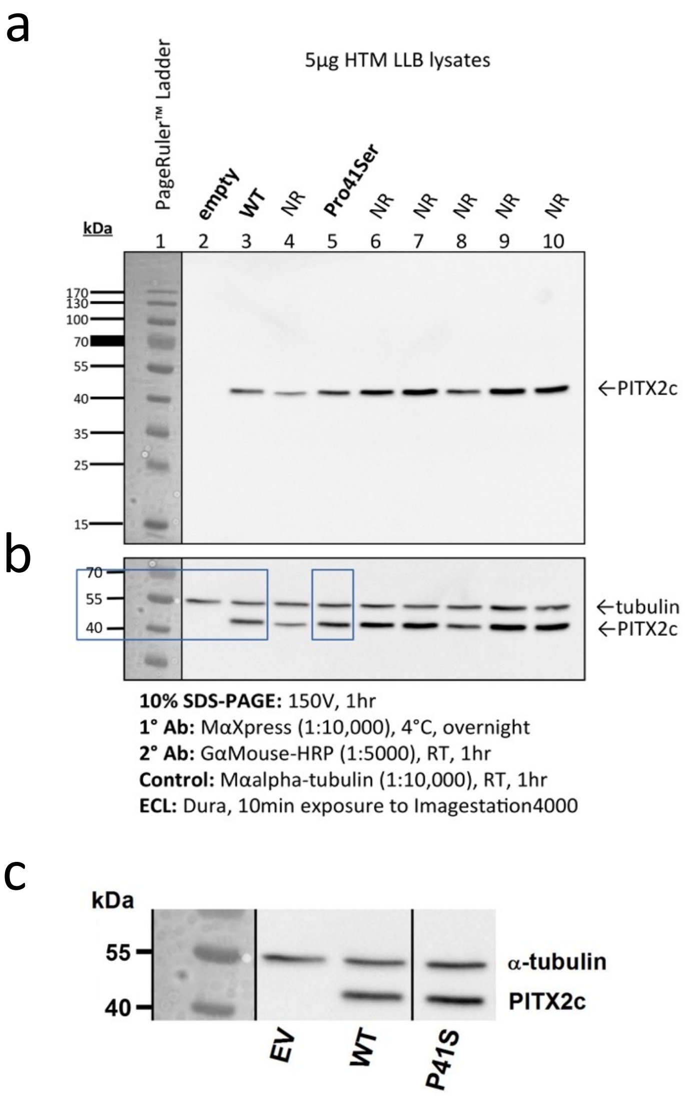

Supplementary Figure 5. (a) Immunofluorescence detection of recombinant PITX2c in TM-1 cells. Merged images of DAPI-labelled nuclei (blue colour) and anti-PITX2c labelling with Anti-Xpress Ab (1:500) as detected by goat Anti-Mouse-IgG coupled to Cyanine3 (1:500) (red colour) in cells transfected with the WT cDNA (left) or the Pro41Ser variant cDNA (right). Red labelling was undetectable outside of nuclear location in TM-1 cells. The scale bar is 20μm**. (b) Upper panel: Electrophoretic mobility shift assays (EMSA) analysis.** Whole-cell extracts from TM-1 cells transfected with cDNA4: Xpress-PITX2c plasmids. Negative control: probe alone (**P**) or with empty vector (**P+EV**) exhibited a single low-mobility shifted background band. Complexes of probe bound to recombinant PITX2c constructs (**WT** and **Pro41Ser**), separated by blank lanes (**B**), were shifted to the same location in the respective lanes, with unbound probe migrating to the bottom of the gel. **Lower panel**: Western analysis shows the expression levels of the Xpress-PITX2c constructs, PITX2c WT (**WT**) and PITX2c **Pro41Ser** in TM-1 cells, in relation to the α-tubulin internal lane control. The data are representative of n=3 separate transfection experiments. Full EMSA gel and WB membrane images are provided in **Supplementary Figure 6**.

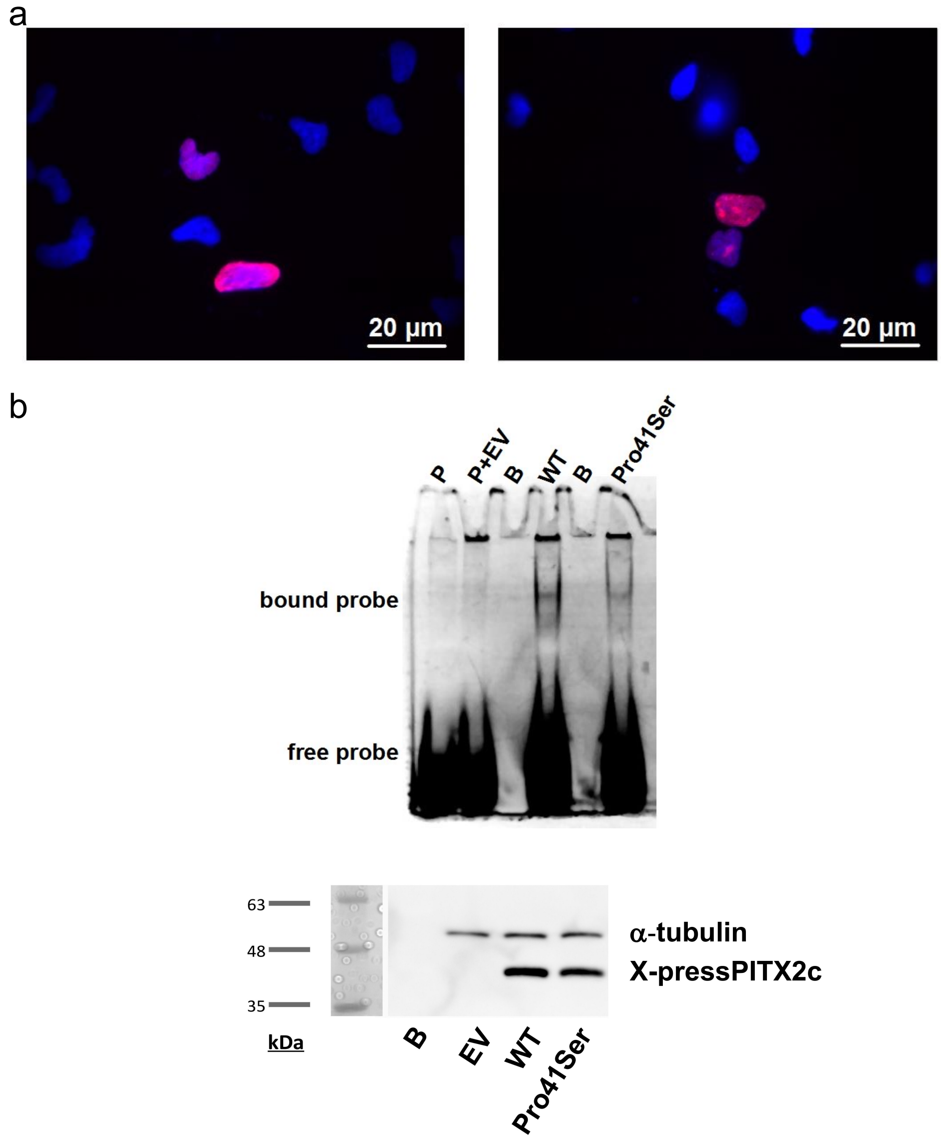

Supplementary Figure 6. Full size images of the EMSA gel. (**a**) and corresponding western blot (**b**). The molecular weight markers (**c**) were run on a separate gel in the same conditions. A cropped image of the markers lane in c was juxtaposed to the western blot image to produce the image in (**d**). **P**: probe only; **P+WT**: probe + PITX2c WT; **B**: blank; **WT**: PITX2c WT; **EV**: empty vector; **Pro41Ser**: PITX2c Pro41Ser; **NR**: not relevant to this study. This figure is provided for showing the absence of undue image manipulation for producing **Supplementary Figures 5b** and **5c.** The data are representative of n=3 separate transfection experiments.

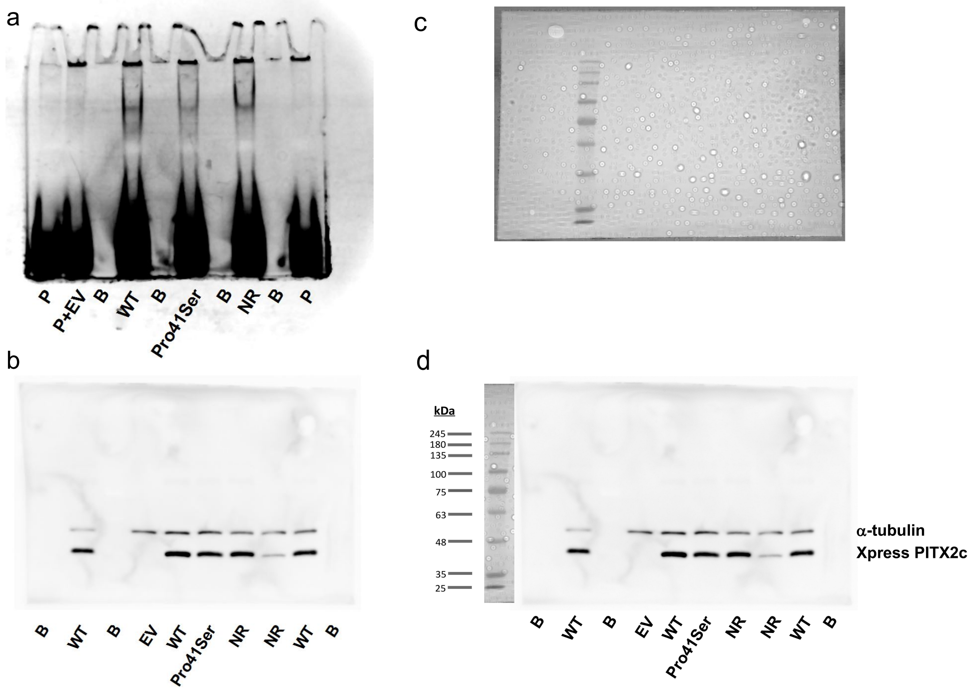

Supplementary File 1 Legend

Interactive Manhattan plot summary of novel sentinel associations. Size of the point is proportional to effect size. $-\log_{10}(p)$ capped at $-\log_{10}(10^{-50})$. Colours indicate disease groups (click [select/deselect trace]/double click [isolate one trace] on the legend to toggle selection). "+" indicate novel variant and gene, "×" indicate novel variant ($r^2<0.2$) not reported in GWAS Catalog/PhenoScanner for the disease. Dotted horizontal lines indicate $-\log_{10}(2x10^{-9})$ [brown] and $-\log_{10}(5x10^{-8})$ [grey]. Hover over the points for detailed information and double click/click on colors/shape in the legends to filter on select groups. Tooltip is available at top right for additional interactive options including: zooming and panning, selection, toggling multiple highlighting of nearby regions on hover and saving as static images.

Supplementary Files 2a-c Legend

Interactive surface plot of effects of cohort specific allele enrichment on inverse variant weighted meta-analysis z-scores (IVW uplift) across MAFs (up to MAF 1%). Uplift is defined as the ratio of meta-analysed IVW Z-score to the Z-score of an individual study. **(a) theoretically predicted IVW uplift. (b) observed IVW uplift. (c) Median absolute relative error (MARE, %) between simulated and theoretical IVW uplift values.** For each combination of MAF and allelic enrichment, we simulated 1000 datasets for two binary variables reflecting disease status. Study sample size and disease prevalence were fixed (matching values estimates from UKB and FG), genomic effects were randomly sampled from the set of positive effect sizes in UKB and FG **(Supplementary Table 3)**, MAF was varied from 0.01% to 1% and allele enrichment (in the smaller study) ranged from 1 to 50. Tooltip is available at top right for additional interactive options including zooming, panning and saving as static images.

Supplementary Table Legends

ST1. Demographics of UKB and FG cohorts
Abbreviations: UKB (UK Biobank); FG (FinnGen); R5 and R6; (FinnGen release 5 and 6 respectively); International Statistical Classification of Diseases and Related Health Problems 10th Revision (ICD10).

ST2. Disease definition and counts in UKB and FG
Phecode/ICD10: Unique disease code identifier, either Phecode or ICD10 code.
FG trait ID: identifier used in FG
FG-UKB similarity score: similarity between FG and UKB defined disease phenotypes (see **Methods**).
Disease cluster ID: unique identifier of grouped diseases (similar disease grouped as one) as defined in **Methods**
UKB/FG cases: UKB/FG number of cases in each cohort (R5 and R6 denote release 5 and 6 of FG respectively)
Additional sex specific analysis: whether additional male/female specific analysis were performed
Post-QC variants analysed: number of genetic variants analysed post-QC filtering (see **Methods).**

ST3. Genome-wide significant associations identified in UKB-FG CWAS meta-analysis
Abbreviations: CWAS (Coding-Wide Association Study); CHR: chromosome; POS: genetic position (hg38); A0, A1 (effect): variant alleles with A1 denoted effect allele which beta relates to; MAF: minor allele frequency in UKB/FG; SE: standard error; META: meta-analysis.

Variant ID: unique variant ID
Region ID: unique (distinct) genetic region ID, where any overlapping regions across all associations are merged into one region (identified by the region ID). See **Methods.**
Disease cluster ID: defined above.
Sex: whether the association came from analysis of both males and females (all), or males/females only
BETA/SE/LOGP_[META/UKB/FG]: effect size (log([OR])/SE/-$\log_{10}(p)$ for meta-analysis/UKB/FG respectively
1-SIFT/PolyPhen: (1-SFIT score)/PolyPhen scores where available.
Novel variant-trait association: indicator (1=novel, 0=reported) for whether the sentinel variant or its proxy (r^2>0.2) for the same/similar trait has been reported previously in GWAS Catalog or PhenoScanner at $p<5x10^{-8}$ (see **Methods**).
Novel gene-trait implication: indicator (1=novel, 0=reported) for whether the associated coding gene has been reported previously (as the annotated gene) in GWAS Catalog (GC) or PhenoScanner (PS) at $p<5x10^{-8}$ (see **Methods**).
Novel ClinVar variant/ ClinVar pathogenicity: indicator (1=novel, 0=reported) for whether the sentinel variant has been reported previously in ClinVar, and if reported previously, what is the assigned ClinVar pathogenicity. See **Methods** and **ST7** for further details.
Manual curation of novel associations: indicator (1=novel, 0=reported) for whether the novel *variant* or *gene* associations were previously reported at $p<5x10^{-8}$ (see **Methods).** Only associations with **Novel variant-trait association** (=1 for both GC and PS) AND **Novel gene-trait implication** (=1 for both GC and PS) AND **Novel ClinVar** (=1) were followed up in manual curations to see whether the associations were novel.

ST4 and ST5. Significant sentinel associations identified in UKB/FG alone
Sentinel associations ($p<5x10^{-8}$) using UKB (ST4) and FG(ST5) cohorts alone and indicator (1=replicated, 0=not replicated) to indicate whether the association was replicated in FG (ST4) or UKB (ST5) at $p<0.05$. Abbreviations and column legend are defined above.

ST6. Allele frequency enrichment between UKB and FG for sentinel CWAS variants
Ordered by magnitude of enrichment. Green: >2 fold enrichment in UKB, Red: >2 fold enrichment in FG.
A1 freq UKB/FG: Allele 1 frequency in UKB/FG.
log2(FG/UKB freq): $\log_2$(FG A1 frequency/UKB A1 frequency).
Enrichment (Fisher's) p: p-value of allele frequencies for UKB and FG from Fisher’s test (two-sided).

ST7. ClinVar annotation of sentinel variants
Gene Info: Gene identified in ClinVar.
Clinical disease: Clinical disease in ClinVar
Matched Trait (FG trait ID): CWAS associated disease trait (FG trait ID – used for more interpretable naming) matching one of the *clinical diseases*.
Putative related traits for unmatched traits (FG ID [possible clinical connection]): curated putatively related CWAS associated diseases (FG trait ID) for clinical diseases with no direct *matched trait.* The possible clinical connection of the relationship is summarised in square parentheses.
ACMG actionable gene: whether the gene is an ACMG (American College of Medical Genetics) clinically actionable gene (see **Methods**).

ST8. List of biomarkers tested in UKB
UKB Field ID: UKB unique field identifier.
Participants: number of individuals with measurement.
Category: Broad category of biomarkers.
Biochemistry subcategory: Additional subcategory for "blood biochemistry" and "urine assays" categories, as defined by UKB in https://www.ukbiobank.ac.uk/media/oiudpjqa/bcm023_ukb_biomarker_panel_website_v1-0-aug-2015-edit-2018.pdf

ST9. Significant associations ($p<10^{-6}$) of CWAS sentinel variants with biomarkers
BETA: effect size (*c.f.* log[OR] in disease association BETA columns).
Other column names as defined above.

ST10. List of drugs targeting genes with a significant coding association
Abbreviations: CT (Clinical trial); MOA (Mechanism of Action); TTD (Therapeutic Target Database)
Target Gene: The gene/gene encoding the protein target of the drug
TTD Target ID/Drug ID: unique identifier used for the target/drug in TTD

ST11. Enrichment of significant genes for approved drug target
Abbreviations: OR (Odds Ratio); CI (Confidence Interval)
Fisher's test (two-sided) used.

ST12. Proteins containing METTL11B target motifs with elevated expression in cardiomyocytes
Uniprot: UniProt identifier
Entry name: UniProt entry name
Gene names: Gene name and aliases for the protein
Motif (single letter amino acids): protein position of motif: P (Proline), A (Alanine), L (Lysine), S (Serine)

ST13. Clustered-MR estimates of overlapping GWAS and CWAS gene set
Abbreviations: MR (Mendelian randomization)
_gwa: MR using AF GWAS results (Nielsen et al)
Cluster label: ID label of the MR cluster
Cluster mean: MR estimate of the cluster
Ratio est: MR estimate of each gene locus
Cluster class: whether the locus belongs to one of the cluster labels or part of the "null" class (see **Methods**)

ST14. PITX2c Pro41Ser variant effects on the mRNA levels for selected proteins involved in AF.
Quantification of PITX2c expression in HL-1 cells transfected with either the WT or the Pro41Ser-mutated PITX2c plasmid (Pro41Ser) normalized to GADPH internal control.
Levels of mRNA for connexins and ion channels in HL-1 cells, either non-transfected (NT) or transfected with an empty vector (EV) (i.e: the V5-Tag-pcDNA3.1/Zeo vector devoid of any PITX2 sequence).

Abbreviations: WT (Wild-type); SEM (Standard Error of the Mean).
n=3 transfections with n=3 repeats of dosage on each cell dish extract. Two-sided t-test used.

Biobank contributions to FinnGen

Auria Biobank (www.auria.fi/biopankki),
THL Biobank (www.thl.fi/biobank)
Helsinki Biobank (www.helsinginbiopankki.fi)
Biobank Borealis of Northern Finland (https://www.ppshp.fi/Tutkimus-ja-opetus/Biopankki/Pages/Biobank-Borealis-briefly-in-English.aspx)
Finnish Clinical Biobank Tampere (www.tays.fi/en-US/Research_and_development/Finnish_Clinical_Biobank_Tampere)
Biobank of Eastern Finland (www.ita-suomenbiopankki.fi/en)
Central Finland Biobank (www.ksshp.fi/fi-FI/Potilaalle/Biopankki)
Finnish Red Cross Blood Service Biobank (www.veripalvelu.fi/verenluovutus/biopankkitoiminta)
Terveystalo Biobank (www.terveystalo.com/fi/Yritystietoa/Terveystalo-Biopankki/Biopankki/)

All Finnish Biobanks are members of BBMRI.fi infrastructure (www.bbmri.fi) and FinBB (https://finbb.fi/).

FinnGen ethics statement details

Patients and control subjects in FinnGen provided informed consent for biobank research, based on the Finnish Biobank Act. Alternatively, separate research cohorts, collected prior the Finnish Biobank Act came into effect (in September 2013) and start of FinnGen (August 2017), were collected based on study-specific consents and later transferred to the Finnish biobanks after approval by Fimea, the National Supervisory Authority for Welfare and Health. Recruitment protocols followed the biobank protocols approved by Fimea. The Coordinating Ethics Committee of the Hospital District of Helsinki and Uusimaa (HUS) approved the FinnGen study protocol Nr HUS/990/2017.

The FinnGen study is approved by Finnish Institute for Health and Welfare (permit numbers: THL/2031/6.02.00/2017, THL/1101/5.05.00/2017, THL/341/6.02.00/2018, THL/2222/6.02.00/2018, THL/283/6.02.00/2019, THL/1721/5.05.00/2019, THL/1524/5.05.00/2020, and THL/2364/14.02/2020), Digital and population data service agency (permit numbers: VRK43431/2017-3, VRK/6909/2018-3, VRK/4415/2019-3), the Social Insurance Institution (permit numbers: KELA 58/522/2017, KELA 131/522/2018, KELA 70/522/2019, KELA 98/522/2019, KELA 138/522/2019, KELA 2/522/2020, KELA 16/522/2020 and Statistics Finland (permit numbers: TK-53-1041-17 and TK-53-90-20).

The Biobank Access Decisions for FinnGen samples and data utilized in FinnGen Data Freeze 6 include: THL Biobank BB2017_55, BB2017_111, BB2018_19, BB_2018_34, BB_2018_67, BB2018_71, BB2019_7, BB2019_8, BB2019_26, BB2020_1, Finnish Red Cross Blood Service Biobank 7.12.2017, Helsinki Biobank HUS/359/2017, Auria Biobank AB17-5154, Biobank Borealis of Northern Finland_2017_1013, Biobank of Eastern Finland 1186/2018, Finnish Clinical Biobank Tampere MH0004, Central Finland Biobank 1-2017, and Terveystalo Biobank STB 2018001.

FinnGen consortium contributors

Steering Committee

Aarno Palotie	Institute for Molecular Medicine Finland, HiLIFE, University of Helsinki, Finland
Mark Daly	Institute for Molecular Medicine Finland, HiLIFE, University of Helsinki, Finland

Pharmaceutical companies

Bridget Riley-Gills	Abbvie, Chicago, IL, United States
Howard Jacob	Abbvie, Chicago, IL, United States
Dirk Paul	Astra Zeneca, Cambridge, United Kingdom
Heiko Runz	Biogen, Cambridge, MA, United States
Sally John	Biogen, Cambridge, MA, United States
Robert Plenge	Celgene, Summit, NJ, United States/Bristol Myers Squibb, New York, NY, United States
Mark McCarthy	Genentech, San Francisco, CA, United States
Julie Hunkapiller	Genentech, San Francisco, CA, United States
Meg Ehm	GlaxoSmithKline, Brentford, United Kingdom
Kirsi Auro	GlaxoSmithKline, Brentford, United Kingdom
Caroline Fox	Merck, Kenilworth, NJ, United States
Anders Mälarstig	Pfizer, New York, NY, United States
Katherine Klinger	Sanofi, Paris, France
Deepak Raipal	Sanofi, Paris, France
Tim Behrens	Maze Therapeutics, San Francisco, CA, United States
Robert Yang	Janssen Biotech, Beerse, Belgium
Richard Siegel	Novartis, Basel, Switzerland

University of Helsinki & Biobanks

Tomi Mäkelä	HiLIFE, University of Helsinki, Finland, Finland
Jaakko Kaprio	Institute for Molecular Medicine Finland, HiLIFE, Helsinki, Finland, Finland
Petri Virolainen	Auria Biobank / University of Turku / Hospital District of Southwest Finland, Turku, Finland
Antti Hakanen	Auria Biobank / University of Turku / Hospital District of Southwest Finland, Turku, Finland
Terhi Kilpi	THL Biobank / The National Institute of Health and Welfare Helsinki, Finland
Markus Perola	THL Biobank / The National Institute of Health and Welfare Helsinki, Finland
Jukka Partanen	Finnish Red Cross Blood Service / Finnish Hematology Registry and Clinical Biobank, Helsinki, Finland
Anne Pitkäranta	Helsinki Biobank / Helsinki University and Hospital District of Helsinki and Uusimaa, Helsinki
Juhani Junttila	Northern Finland Biobank Borealis / University of Oulu / Northern Ostrobothnia Hospital District, Oulu, Finland
Raisa Serpi	Northern Finland Biobank Borealis / University of Oulu / Northern Ostrobothnia Hospital District, Oulu, Finland
Tarja Laitinen	Finnish Clinical Biobank Tampere / University of Tampere / Pirkanmaa Hospital District, Tampere, Finland
Johanna Mäkelä	Finnish Clinical Biobank Tampere / University of Tampere / Pirkanmaa Hospital District, Tampere, Finland
Veli-Matti Kosma	Biobank of Eastern Finland / University of Eastern Finland / Northern Savo Hospital District, Kuopio, Finland
Urho Kujala	Central Finland Biobank / University of Jyväskylä / Central Finland Health Care District, Jyväskylä, Finland

Other Experts/ Non-Voting Members

Outi Tuovila	Business Finland, Helsinki, Finland
Raimo Pakkanen	Business Finland, Helsinki, Finland

Scientific Committee

Pharmaceutical companies

Jeffrey Waring	Abbvie, Chicago, IL, United States
Ali Abbasi	Abbvie, Chicago, IL, United States
Mengzhen Liu	Abbvie, Chicago, IL, United States
Ioanna Tachmazidou	Astra Zeneca, Cambridge, United Kingdom
Chia-Yen Chen	Biogen, Cambridge, MA, United States
Heiko Runz	Biogen, Cambridge, MA, United States

Shameek Biswas	Celgene, Summit, NJ, United States/Bristol Myers Squibb, New York, NY, United States
Julie Hunkapiller	Genentech, San Francisco, CA, United States
Meg Ehm	GlaxoSmithKline, Brentford, United Kingdom
Neha Raghavan	Merck, Kenilworth, NJ, United States
Adriana Huertas-Vazquez	Merck, Kenilworth, NJ, United States
Anders Mälarstig	Pfizer, New York, NY, United States
Xinli Hu	Pfizer, New York, NY, United States
Katherine Klinger	Sanofi, Paris, France
Matthias Gossel	Sanofi, Paris, France
Robert Graham	Maze Therapeutics, San Francisco, CA, United States
Tim Behrens	Maze Therapeutics, San Francisco, CA, United States
Beryl Cummings	Maze Therapeutics, San Francisco, CA, United States
Wilco Fleuren	Janssen Biotech, Beerse, Belgium
Dawn Waterworth	Janssen Biotech, Beerse, Belgium
Nicole Renaud	Novartis, Basel, Switzerland
Aviv Madar	Novartis, Basel, Switzerland
Maen Obeidat	Novartis, Basel, Switzerland
**University of Helsinki & Biobanks**	
Samuli Ripatti	Institute for Molecular Medicine Finland, HiLIFE, Helsinki, Finland
Johanna Schleutker	Auria Biobank / Univ. of Turku / Hospital District of Southwest Finland, Turku, Finland
Markus Perola	THL Biobank / The National Institute of Health and Welfare Helsinki, Finland
Mikko Arvas	Finnish Red Cross Blood Service / Finnish Hematology Registry and Clinical Biobank, Helsinki, Finland
Olli Carpén	Helsinki Biobank / Helsinki University and Hospital District of Helsinki and Uusimaa, Helsinki
Reetta Hinttala	Northern Finland Biobank Borealis / University of Oulu / Northern Ostrobothnia Hospital District, Oulu, Finland
Johannes Kettunen	Northern Finland Biobank Borealis / University of Oulu / Northern Ostrobothnia Hospital District, Oulu, Finland
Johanna Mäkelä	Finnish Clinical Biobank Tampere / University of Tampere / Pirkanmaa Hospital District, Tampere, Finland
Arto Mannermaa	Biobank of Eastern Finland / University of Eastern Finland / Northern Savo Hospital District, Kuopio, Finland
Jari Laukkanen	Central Finland Biobank / University of Jyväskylä / Central Finland Health Care District, Jyväskylä, Finland
Urho Kujala	Central Finland Biobank / University of Jyväskylä / Central Finland Health Care District, Jyväskylä, Finland

Clinical Groups

Neurology Group

Reetta Kälviäinen	Northern Savo Hospital District, Kuopio, Finland
Valtteri Julkunen	Northern Savo Hospital District, Kuopio, Finland
Hilkka Soininen	Northern Savo Hospital District, Kuopio, Finland
Anne Remes	Northern Ostrobothnia Hospital District, Oulu, Finland
Mikko Hiltunen	Northern Savo Hospital District, Kuopio, Finland
Jukka Peltola	Pirkanmaa Hospital District, Tampere, Finland
Pentti Tienari	Hospital District of Helsinki and Uusimaa, Helsinki, Finland
Juha Rinne	Hospital District of Southwest Finland, Turku, Finland
Roosa Kallionpää	Hospital District of Southwest Finland, Turku, Finland
Ali Abbasi	Abbvie, Chicago, IL, United States
Adam Ziemann	Abbvie, Chicago, IL, United States
Jeffrey Waring	Abbvie, Chicago, IL, United States
Sahar Esmaeeli	Abbvie, Chicago, IL, United States
Nizar Smaoui	Abbvie, Chicago, IL, United States
Anne Lehtonen	Abbvie, Chicago, IL, United States
Susan Eaton	Biogen, Cambridge, MA, United States
Heiko Runz	Biogen, Cambridge, MA, United States
Sanni Lahdenperä	Biogen, Cambridge, MA, United States

Janet van Adelsberg	Celgene, Summit, NJ, United States/ Bristol Myers Squibb, New York, NY, United States
Shameek Biswas	Celgene, Summit, NJ, United States/ Bristol Myers Squibb, New York, NY, United States
Julie Hunkapiller	Genentech, San Francisco, CA, United States
Natalie Bowers	Genentech, San Francisco, CA, United States
Edmond Teng	Genentech, San Francisco, CA, United States
Sarah Pendergrass	Genentech, San Francisco, CA, United States
Onuralp Soylemez	Merck, Kenilworth, NJ, United States
Kari Linden	Pfizer, New York, NY, United States
Fanli Xu	GlaxoSmithKline, Brentford, United Kingdom
David Pulford	GlaxoSmithKline, Brentford, United Kingdom
Kirsi Auro	GlaxoSmithKline, Brentford, United Kingdom
Laura Addis	GlaxoSmithKline, Brentford, United Kingdom
John Eicher	GlaxoSmithKline, Brentford, United Kingdom
Minna Raivio	Hospital District of Helsinki and Uusimaa, Helsinki, Finland
Sarah Pendergrass	Genentech, San Francisco, CA, United States
Beryl Cummings	Maze Therapeutics, San Francisco, CA, United States
Juulia Partanen	Institute for Molecular Medicine Finland, HiLIFE, University of Helsinki, Finland

Gastroenterology Group

Martti Färkkilä	Hospital District of Helsinki and Uusimaa, Helsinki, Finland
Jukka Koskela	Hospital District of Helsinki and Uusimaa, Helsinki, Finland
Sampsa Pikkarainen	Hospital District of Helsinki and Uusimaa, Helsinki, Finland
Airi Jussila	Pirkanmaa Hospital District, Tampere, Finland
Katri Kaukinen	Pirkanmaa Hospital District, Tampere, Finland
Timo Blomster	Northern Ostrobothnia Hospital District, Oulu, Finland
Mikko Kiviniemi	Northern Savo Hospital District, Kuopio, Finland
Markku Voutilainen	Hospital District of Southwest Finland, Turku, Finland
Ali Abbasi	Abbvie, Chicago, IL, United States
Graham Heap	Abbvie, Chicago, IL, United States
Jeffrey Waring	Abbvie, Chicago, IL, United States
Nizar Smaoui	Abbvie, Chicago, IL, United States
Fedik Rahimov	Abbvie, Chicago, IL, United States
Anne Lehtonen	Abbvie, Chicago, IL, United States
Keith Usiskin	Celgene, Summit, NJ, United States/ Bristol Myers Squibb, New York, NY, United States
Tim Lu	Genentech, San Francisco, CA, United States
Natalie Bowers	Genentech, San Francisco, CA, United States
Danny Oh	Genentech, San Francisco, CA, United States
Sarah Pendergrass	Genentech, San Francisco, CA, United States
Kirsi Kalpala	Pfizer, New York, NY, United States
Melissa Miller	Pfizer, New York, NY, United States
Xinli Hu	Pfizer, New York, NY, United States
Linda McCarthy	GlaxoSmithKline, Brentford, United Kingdom
Onuralp Soylemez	Merck, Kenilworth, NJ, United States
Mark Daly	Institute for Molecular Medicine Finland, HiLIFE, University of Helsinki, Finland

Rheumatology Group

Kari Eklund	Hospital District of Helsinki and Uusimaa, Helsinki, Finland
Antti Palomäki	Hospital District of Southwest Finland, Turku, Finland
Pia Isomäki	Pirkanmaa Hospital District, Tampere, Finland
Laura Pirilä	Hospital District of Southwest Finland, Turku, Finland
Oili Kaipiainen-Seppänen	Northern Savo Hospital District, Kuopio, Finland
Johanna Huhtakangas	Northern Ostrobothnia Hospital District, Oulu, Finland
Ali Abbasi	Abbvie, Chicago, IL, United States
Jeffrey Waring	Abbvie, Chicago, IL, United States
Fedik Rahimov	Abbvie, Chicago, IL, United States
Apinya Lertratanakul	Abbvie, Chicago, IL, United States
Nizar Smaoui	Abbvie, Chicago, IL, United States
Anne Lehtonen	Abbvie, Chicago, IL, United States
David Close	Astra Zeneca, Cambridge, United Kingdom

Marla Hochfeld	Celgene, Summit, NJ, United States/ Bristol Myers Squibb, New York, NY, United States
Natalie Bowers	Genentech, San Francisco, CA, United States
Sarah Pendergrass	Genentech, San Francisco, CA, United States
Onuralp Soylemez	Merck, Kenilworth, NJ, United States
Kirsi Kalpala	Pfizer, New York, NY, United States
Nan Bing	Pfizer, New York, NY, United States
Xinli Hu	Pfizer, New York, NY, United States
Jorge Esparza Gordillo	GlaxoSmithKline, Brentford, United Kingdom
Kirsi Auro	GlaxoSmithKline, Brentford, United Kingdom
Dawn Waterworth	Janssen Biotech, Beerse, Belgium
Nina Mars	Institute for Molecular Medicine Finland, HiLIFE, Helsinki, Finland

Pulmonology Group

Tarja Laitinen	Pirkanmaa Hospital District, Tampere, Finland
Margit Pelkonen	Northern Savo Hospital District, Kuopio, Finland
Paula Kauppi	Hospital District of Helsinki and Uusimaa, Helsinki, Finland
Hannu Kankaanranta	Pirkanmaa Hospital District, Tampere, Finland
Terttu Harju	Northern Ostrobothnia Hospital District, Oulu, Finland
Riitta Lahesmaa	Hospital District of Southwest Finland, Turku, Finland
Nizar Smaoui	Abbvie, Chicago, IL, United States
Alex Mackay	Astra Zeneca, Cambridge, United Kingdom
Glenda Lassi	Astra Zeneca, Cambridge, United Kingdom
Susan Eaton	Biogen, Cambridge, MA, United States
Steven Greenberg	Celgene, Summit, NJ, United States/ Bristol Myers Squibb, New York, NY, United States
Hubert Chen	Genentech, San Francisco, CA, United States
Sarah Pendergrass	Genentech, San Francisco, CA, United States
Natalie Bowers	Genentech, San Francisco, CA, United States
Joanna Betts	GlaxoSmithKline, Brentford, United Kingdom
Soumitra Ghosh	GlaxoSmithKline, Brentford, United Kingdom
Kirsi Auro	GlaxoSmithKline, Brentford, United Kingdom
Rajashree Mishra	GlaxoSmithKline, Brentford, United Kingdom
Sina Rüeger	Institute for Molecular Medicine Finland, HiLIFE, University of Helsinki, Finland

Cardiometabolic Diseases Group

Teemu Niiranen	The National Institute of Health and Welfare Helsinki, Finland
Felix Vaura	The National Institute of Health and Welfare Helsinki, Finland
Veikko Salomaa	The National Institute of Health and Welfare Helsinki, Finland
Markus Juonala	Hospital District of Southwest Finland, Turku, Finland
Kaj Metsärinne	Hospital District of Southwest Finland, Turku, Finland
Mika Kähönen	Pirkanmaa Hospital District, Tampere, Finland
Juhani Junttila	Northern Ostrobothnia Hospital District, Oulu, Finland
Markku Laakso	Northern Savo Hospital District, Kuopio, Finland
Jussi Pihlajamäki	Northern Savo Hospital District, Kuopio, Finland
Daniel Gordin	Hospital District of Helsinki and Uusimaa, Helsinki, Finland
Juha Sinisalo	Hospital District of Helsinki and Uusimaa, Helsinki, Finland
Marja-Riitta Taskinen	Hospital District of Helsinki and Uusimaa, Helsinki, Finland
Tiinamaija Tuomi	Hospital District of Helsinki and Uusimaa, Helsinki, Finland
Jari Laukkanen	Central Finland Health Care District, Jyväskylä, Finland
Benjamin Challis	Astra Zeneca, Cambridge, United Kingdom
Dirk Paul	Astra Zeneca, Cambridge, United Kingdom
Julie Hunkapiller	Genentech, San Francisco, CA, United States
Natalie Bowers	Genentech, San Francisco, CA, United States
Sarah Pendergrass	Genentech, San Francisco, CA, United States
Onuralp Soylemez	Merck, Kenilworth, NJ, United States
Jaakko Parkkinen	Pfizer, New York, NY, United States
Melissa Miller	Pfizer, New York, NY, United States
Russell Miller	Pfizer, New York, NY, United States
Audrey Chu	GlaxoSmithKline, Brentford, United Kingdom
Kirsi Auro	GlaxoSmithKline, Brentford, United Kingdom

Keith Usiskin Celgene, Summit, NJ, United States/ Bristol Myers Squibb, New York, NY, United States
Amanda Elliott Institute for Molecular Medicine Finland, HiLIFE, University of Helsinki, Finland / Broad Institute, Cambridge, MA, United States
Joel Rämö Institute for Molecular Medicine Finland, HiLIFE, University of Helsinki, Finland
Samuli Ripatti Institute for Molecular Medicine Finland, HiLIFE, University of Helsinki, Finland
Mary Pat Reeve Institute for Molecular Medicine Finland, HiLIFE, University of Helsinki, Finland
Sanni Ruotsalainen Institute for Molecular Medicine Finland, HiLIFE, University of Helsinki, Finland

Oncology Group

Tuomo Meretoja Hospital District of Helsinki and Uusimaa, Helsinki, Finland
Heikki Joensuu Hospital District of Helsinki and Uusimaa, Helsinki, Finland
Olli Carpén Hospital District of Helsinki and Uusimaa, Helsinki, Finland
Lauri Aaltonen Hospital District of Helsinki and Uusimaa, Helsinki, Finland
Johanna Mattson Hospital District of Helsinki and Uusimaa, Helsinki, Finland
Annika Auranen Pirkanmaa Hospital District , Tampere, Finland
Peeter Karihtala Northern Ostrobothnia Hospital District, Oulu, Finland
Saila Kauppila Northern Ostrobothnia Hospital District, Oulu, Finland
Päivi Auvinen Northern Savo Hospital District, Kuopio, Finland
Klaus Elenius Hospital District of Southwest Finland, Turku, Finland
Johanna Schleutker Hospital District of Southwest Finland, Turku, Finland
Relja Popovic Abbvie, Chicago, IL, United States
Jeffrey Waring Abbvie, Chicago, IL, United States
Bridget Riley-Gillis Abbvie, Chicago, IL, United States
Anne Lehtonen Abbvie, Chicago, IL, United States
Jennifer Schutzman Genentech, San Francisco, CA, United States
Julie Hunkapiller Genentech, San Francisco, CA, United States
Natalie Bowers Genentech, San Francisco, CA, United States
Sarah Pendergrass Genentech, San Francisco, CA, United States
Andrey Loboda Merck, Kenilworth, NJ, United States
Aparna Chhibber Merck, Kenilworth, NJ, United States
Heli Lehtonen Pfizer, New York, NY, United States
Stefan McDonough Pfizer, New York, NY, United States
Marika Crohns Sanofi, Paris, France
Sauli Vuoti Sanofi, Paris, France
Diptee Kulkarni GlaxoSmithKline, Brentford, United Kingdom
Kirsi Auro GlaxoSmithKline, Brentford, United Kingdom
Esa Pitkänen Institute for Molecular Medicine Finland, HiLIFE, University of Helsinki, Finland
Nina Mars Institute for Molecular Medicine Finland, HiLIFE, University of Helsinki, Finland
Mark Daly Institute for Molecular Medicine Finland, HiLIFE, University of Helsinki, Finland

Ophthalmology Group

Kai Kaarniranta Northern Savo Hospital District, Kuopio, Finland
Joni A Turunen Hospital District of Helsinki and Uusimaa, Helsinki, Finland
Terhi Ollila Hospital District of Helsinki and Uusimaa, Helsinki, Finland
Sanna Seitsonen Hospital District of Helsinki and Uusimaa, Helsinki, Finland
Hannu Uusitalo Pirkanmaa Hospital District, Tampere, Finland
Vesa Aaltonen Hospital District of Southwest Finland, Turku, Finland
Hannele Uusitalo-Järvinen Pirkanmaa Hospital District, Tampere, Finland
Marja Luodonpää Northern Ostrobothnia Hospital District, Oulu, Finland
Nina Hautala Northern Ostrobothnia Hospital District, Oulu, Finland
Mengzhen Liu Abbvie, Chicago, IL, United States
Heiko Runz Biogen, Cambridge, MA, United States
Stephanie Loomis Biogen, Cambridge, MA, United States
Erich Strauss Genentech, San Francisco, CA, United States
Natalie Bowers Genentech, San Francisco, CA, United States
Hao Chen Genentech, San Francisco, CA, United States
Sarah Pendergrass Genentech, San Francisco, CA, United States
Anna Podgornaia Merck, Kenilworth, NJ, United States
Juha Karjalainen Institute for Molecular Medicine Finland, HiLIFE, University of Helsinki, Finland / Broad Institute, Cambridge, MA, United States
Esa Pitkänen Institute for Molecular Medicine Finland, HiLIFE, University of Helsinki, Finland

Dermatology Group

Kaisa Tasanen	Northern Ostrobothnia Hospital District, Oulu, Finland
Laura Huilaja	Northern Ostrobothnia Hospital District, Oulu, Finland
Katariina Hannula-Jouppi	Hospital District of Helsinki and Uusimaa, Helsinki, Finland
Teea Salmi	Pirkanmaa Hospital District, Tampere, Finland
Sirkku Peltonen	Hospital District of Southwest Finland, Turku, Finland
Leena Koulu	Hospital District of Southwest Finland, Turku, Finland
Kirsi Kalpala	Pfizer, New York, NY, United States
Ying Wu	Pfizer, New York, NY, United States
David Choy	Genentech, San Francisco, CA, United States
Sarah Pendergrass	Genentech, San Francisco, CA, United States
Nizar Smaoui	Abbvie, Chicago, IL, United States
Fedik Rahimov	Abbvie, Chicago, IL, United States
Anne Lehtonen	Abbvie, Chicago, IL, United States
Dawn Waterworth	Janssen Biotech, Beerse, Belgium

Odontology Group

Pirkko Pussinen	Hospital District of Helsinki and Uusimaa, Helsinki, Finland
Aino Salminen	Hospital District of Helsinki and Uusimaa, Helsinki, Finland
Tuula Salo	Hospital District of Helsinki and Uusimaa, Helsinki, Finland
David Rice	Hospital District of Helsinki and Uusimaa, Helsinki, Finland
Pekka Nieminen	Hospital District of Helsinki and Uusimaa, Helsinki, Finland
Ulla Palotie	Hospital District of Helsinki and Uusimaa, Helsinki, Finland
Juha Sinisalo	Hospital District of Helsinki and Uusimaa, Helsinki, Finland
Maria Siponen	Northern Savo Hospital District, Kuopio, Finland
Liisa Suominen	Northern Savo Hospital District, Kuopio, Finland
Päivi Mäntylä	Northern Savo Hospital District, Kuopio, Finland
Ulvi Gursoy	Hospital District of Southwest Finland, Turku, Finland
Vuokko Anttonen	Northern Ostrobothnia Hospital District, Oulu, Finland
Kirsi Sipilä	Northern Ostrobothnia Hospital District, Oulu, Finland
Sarah Pendergrass	Genentech, San Francisco, CA, United States

Women's Health and Reproduction Group

Hannele Laivuori	Institute for Molecular Medicine Finland, HiLIFE, University of Helsinki, Finland
Venla Kurra	Pirkanmaa Hospital District, Tampere, Finland
Oskari Heikinheimo	Hospital District of Helsinki and Uusimaa, Helsinki, Finland
Ilkka Kalliala	Hospital District of Helsinki and Uusimaa, Helsinki, Finland
Laura Kotaniemi-Talonen	Pirkanmaa Hospital District, Tampere, Finland
Kari Nieminen	Pirkanmaa Hospital District, Tampere, Finland
Päivi Polo	Hospital District of Southwest Finland, Turku, Finland
Kaarin Mäkikallio	Hospital District of Southwest Finland, Turku, Finland
Eeva Ekholm	Hospital District of Southwest Finland, Turku, Finland
Marja Vääräsmäki	Northern Ostrobothnia Hospital District, Oulu, Finland
Outi Uimari	Northern Ostrobothnia Hospital District, Oulu, Finland
Laure Morin-Papunen	Northern Ostrobothnia Hospital District, Oulu, Finland
Marjo Tuppurainen	Northern Savo Hospital District, Kuopio, Finland
Katja Kivinen	Institute for Molecular Medicine Finland, HiLIFE, University of Helsinki, Finland
Elisabeth Widen	Institute for Molecular Medicine Finland, HiLIFE, University of Helsinki, Finland
Taru Tukiainen	Institute for Molecular Medicine Finland, HiLIFE, University of Helsinki, Finland
Mary Pat Reeve	Institute for Molecular Medicine Finland, HiLIFE, University of Helsinki, Finland
Mark Daly	Institute for Molecular Medicine Finland, HiLIFE, University of Helsinki, Finland
Liu Aoxing	Institute for Molecular Medicine Finland, HiLIFE, University of Helsinki, Finland
Eija Laakkonen	University of Jyväskylä, Jyväskylä, Finland
Niko Välimäki	University of Helsinki, Helsinki, Finland
Lauri Aaltonen	Hospital District of Helsinki and Uusimaa, Helsinki, Finland
Johannes Kettunen	Northern Ostrobothnia Hospital District, Oulu, Finland
Mikko Arvas	Finnish Red Cross Blood Service, Helsinki, Finland
Jeffrey Waring	Abbvie, Chicago, IL, United States
Bridget Riley-Gillis	Abbvie, Chicago, IL, United States
Mengzhen Liu	Abbvie, Chicago, IL, United States
Janet Kumar	GlaxoSmithKline, Brentford, United Kingdom
Kirsi Auro	GlaxoSmithKline, Brentford, United Kingdom

Andrea Ganna	Institute for Molecular Medicine Finland, HiLIFE, University of Helsinki, Finland
Sarah Pendergrass	Genentech, San Francisco, CA, United States

FinnGen Analysis working group

Justin Wade Davis	Abbvie, Chicago, IL, United States
Bridget Riley-Gillis	Abbvie, Chicago, IL, United States
Danjuma Quarless	Abbvie, Chicago, IL, United States
Fedik Rahimov	Abbvie, Chicago, IL, United States
Sahar Esmaeeli	Abbvie, Chicago, IL, United States
Slavé Petrovski	Astra Zeneca, Cambridge, United Kingdom
Eleonor Wigmore	Astra Zeneca, Cambridge, United Kingdom
Adele Mitchell	Biogen, Cambridge, MA, United States
Benjamin Sun	Biogen, Cambridge, MA, United States
Ellen Tsai	Biogen, Cambridge, MA, United States
Denis Baird	Biogen, Cambridge, MA, United States
Paola Bronson	Biogen, Cambridge, MA, United States
Ruoyu Tian	Biogen, Cambridge, MA, United States
Stephanie Loomis	Biogen, Cambridge, MA, United States
Yunfeng Huang	Biogen, Cambridge, MA, United States
Joseph Maranville	Celgene, Summit, NJ, United States/ Bristol Myers Squibb, New York, NY, United States
Shameek Biswas	Celgene, Summit, NJ, United States/ Bristol Myers Squibb, New York, NY, United States
Elmutaz Mohammed	Celgene, Summit, NJ, United States/ Bristol Myers Squibb, New York, NY, United States
Samir Wadhawan	Celgene, Summit, NJ, United States/ Bristol Myers Squibb, New York, NY, United States
Erika Kvikstad	Celgene, Summit, NJ, United States/ Bristol Myers Squibb, New York, NY, United States
Minal Caliskan	Celgene, Summit, NJ, United States/ Bristol Myers Squibb, New York, NY, United States
Diana Chang	Genentech, San Francisco, CA, United States
Julie Hunkapiller	Genentech, San Francisco, CA, United States
Tushar Bhangale	Genentech, San Francisco, CA, United States
Natalie Bowers	Genentech, San Francisco, CA, United States
Sarah Pendergrass	Genentech, San Francisco, CA, United States
Kirill Shkura	Merck, Kenilworth, NJ, United States
Victor Neduva	Merck, Kenilworth, NJ, United States
Xing Chen	Pfizer, New York, NY, United States
Åsa Hedman	Pfizer, New York, NY, United States
Karen S King	GlaxoSmithKline, Brentford, United Kingdom
Padhraig Gormley	GlaxoSmithKline, Brentford, United Kingdom
Jimmy Liu	GlaxoSmithKline, Brentford, United Kingdom
Clarence Wang	Sanofi, Paris, France
Ethan Xu	Sanofi, Paris, France
Franck Auge	Sanofi, Paris, France
Clement Chatelain	Sanofi, Paris, France
Deepak Rajpal	Sanofi, Paris, France
Dongyu Liu	Sanofi, Paris, France
Katherine Call	Sanofi, Paris, France
Tai-He Xia	Sanofi, Paris, France
Beryl Cummings	Maze Therapeutics, San Francisco, CA, United States
Matt Brauer	Maze Therapeutics, San Francisco, CA, United States
Huilei Xu	Novartis, Basel, Switzerland
Amy Cole	Novartis, Basel, Switzerland
Jonathan Chung	Novartis, Basel, Switzerland
Jaison Jacob	Novartis, Basel, Switzerland
Katrina de Lange	Novartis, Basel, Switzerland
Jonas Zierer	Novartis, Basel, Switzerland

Mitja Kurki Institute for Molecular Medicine Finland, HiLIFE, University of Helsinki, Finland / Broad Institute, Cambridge, MA, United States
Samuli Ripatti Institute for Molecular Medicine Finland, HiLIFE, University of Helsinki, Finland
Mark Daly Institute for Molecular Medicine Finland, HiLIFE, University of Helsinki, Finland
Juha Karjalainen Institute for Molecular Medicine Finland, HiLIFE, University of Helsinki, Finland / Broad Institute, Cambridge, MA, United States
Aki Havulinna Institute for Molecular Medicine Finland, HiLIFE, University of Helsinki, Finland
Juha Mehtonen Institute for Molecular Medicine Finland, HiLIFE, University of Helsinki, Finland
Priit Palta Institute for Molecular Medicine Finland, HiLIFE, University of Helsinki, Finland
Shabbeer Hassan Institute for Molecular Medicine Finland, HiLIFE, University of Helsinki, Finland
Pietro Della Briotta Parolo Institute for Molecular Medicine Finland, HiLIFE, University of Helsinki, Finland
Wei Zhou Broad Institute, Cambridge, MA, United States
Mutaamba Maasha Broad Institute, Cambridge, MA, United States
Shabbeer Hassan Institute for Molecular Medicine Finland, HiLIFE, University of Helsinki, Finland
Susanna Lemmelä Institute for Molecular Medicine Finland, HiLIFE, University of Helsinki, Finland
Manuel Rivas University of Stanford, Stanford, CA, United States
Aarno Palotie Institute for Molecular Medicine Finland, HiLIFE, University of Helsinki, Finland
Arto Lehisto Institute for Molecular Medicine Finland, HiLIFE, University of Helsinki, Finland
Andrea Ganna Institute for Molecular Medicine Finland, HiLIFE, University of Helsinki, Finland
Vincent Llorens Institute for Molecular Medicine Finland, HiLIFE, University of Helsinki, Finland
Hannele Laivuori Institute for Molecular Medicine Finland, HiLIFE, University of Helsinki, Finland
Mari E Niemi Institute for Molecular Medicine Finland, HiLIFE, University of Helsinki, Finland
Taru Tukiainen Institute for Molecular Medicine Finland, HiLIFE, University of Helsinki, Finland
Mary Pat Reeve Institute for Molecular Medicine Finland, HiLIFE, University of Helsinki, Finland
Henrike Heyne Institute for Molecular Medicine Finland, HiLIFE, University of Helsinki, Finland
Nina Mars Institute for Molecular Medicine Finland, HiLIFE, University of Helsinki, Finland
Kimmo Palin University of Helsinki, Helsinki, Finland
Javier Garcia-Tabuenca University of Tampere, Tampere, Finland
Harri Siirtola University of Tampere, Tampere, Finland
Tuomo Kiiskinen Institute for Molecular Medicine Finland, HiLIFE, University of Helsinki, Finland
Jiwoo Lee Institute for Molecular Medicine Finland, HiLIFE, University of Helsinki, Finland / Broad Institute, Cambridge, MA, United States
Kristin Tsuo Institute for Molecular Medicine Finland, HiLIFE, University of Helsinki, Finland / Broad Institute, Cambridge, MA, United States
Amanda Elliott Institute for Molecular Medicine Finland, HiLIFE, University of Helsinki, Finland / Broad Institute, Cambridge, MA, United States
Kati Kristiansson THL Biobank / The National Institute of Health and Welfare Helsinki, Finland
Mikko Arvas Finnish Red Cross Blood Service / Finnish Hematology Registry and Clinical Biobank, Helsinki, Finland
Kati Hyvärinen Finnish Red Cross Blood Service, Helsinki, Finland
Jarmo Ritari Finnish Red Cross Blood Service, Helsinki, Finland
Miika Koskinen Helsinki Biobank / Helsinki University and Hospital District of Helsinki and Uusimaa, Helsinki
Olli Carpén Helsinki Biobank / Helsinki University and Hospital District of Helsinki and Uusimaa, Helsinki
Johannes Kettunen Northern Finland Biobank Borealis / University of Oulu / Northern Ostrobothnia Hospital District, Oulu, Finland
Katri Pylkäs University of Oulu, Oulu, Finland
Marita Kalaoja University of Oulu, Oulu, Finland
Minna Karjalainen University of Oulu, Oulu, Finland
Tuomo Mantere Northern Finland Biobank Borealis / University of Oulu / Northern Ostrobothnia Hospital District, Oulu, Finland
Eeva Kangasniemi Finnish Clinical Biobank Tampere / University of Tampere / Pirkanmaa Hospital District, Tampere, Finland
Sami Heikkinen University of Eastern Finland, Kuopio, Finland
Arto Mannermaa Biobank of Eastern Finland / University of Eastern Finland / Northern Savo Hospital District, Kuopio, Finland
Eija Laakkonen University of Jyväskylä, Jyväskylä, Finland
Samuel Heron University of Turku, Turku, Finland
Dhanaprakash Jambulingam University of Turku, Turku, Finland

Venkat Subramaniam Rathinakannan University of Turku, Turku, Finland
Nina Pitkänen Auria Biobank / University of Turku / Hospital District of Southwest Finland, Turku, Finland

Biobank directors

Lila Kallio Auria Biobank / University of Turku / Hospital District of Southwest Finland, Turku, Finland
Sirpa Soini THL Biobank / The National Institute of Health and Welfare Helsinki, Finland
Jukka Partanen Finnish Red Cross Blood Service / Finnish Hematology Registry and Clinical Biobank, Helsinki, Finland
Eero Punkka Helsinki Biobank / Helsinki University and Hospital District of Helsinki and Uusimaa, Helsinki
Raisa Serpi Northern Finland Biobank Borealis / University of Oulu / Northern Ostrobothnia Hospital District, Oulu, Finland
Johanna Mäkelä Finnish Clinical Biobank Tampere / University of Tampere / Pirkanmaa Hospital District, Tampere, Finland
Veli-Matti Kosma Biobank of Eastern Finland / University of Eastern Finland / Northern Savo Hospital District, Kuopio, Finland
Teijo Kuopio Central Finland Biobank / University of Jyväskylä / Central Finland Health Care District, Jyväskylä, Finland

FinnGen Teams

Administration
Anu Jalanko Institute for Molecular Medicine Finland, HiLIFE, University of Helsinki, Finland
Huei-Yi Shen Institute for Molecular Medicine Finland, HiLIFE, University of Helsinki, Finland
Risto Kajanne Institute for Molecular Medicine Finland, HiLIFE, University of Helsinki, Finland
Mervi Aavikko Institute for Molecular Medicine Finland, HiLIFE, University of Helsinki, Finland
Analysis
Mitja Kurki Institute for Molecular Medicine Finland, HiLIFE, University of Helsinki, Finland / Broad Institute, Cambridge, MA, United States
Juha Karjalainen Institute for Molecular Medicine Finland, HiLIFE, University of Helsinki, Finland / Broad Institute, Cambridge, MA, United States
Pietro Della Briotta Parolo Institute for Molecular Medicine Finland, HiLIFE, University of Helsinki, Finland
Arto Lehisto Institute for Molecular Medicine Finland, HiLIFE, University of Helsinki, Finland
Juha Mehtonen Institute for Molecular Medicine Finland, HiLIFE, University of Helsinki, Finland
Wei Zhou Broad Institute, Cambridge, MA, United States
Masahiro Kanai Broad Institute, Cambridge, MA, United States
Mutaamba Maasha Broad Institute, Cambridge, MA, United States
Clinical Endpoint Development
Hannele Laivuori Institute for Molecular Medicine Finland, HiLIFE, University of Helsinki, Finland
Aki Havulinna Institute for Molecular Medicine Finland, HiLIFE, University of Helsinki, Finland
Susanna Lemmelä Institute for Molecular Medicine Finland, HiLIFE, University of Helsinki, Finland
Tuomo Kiiskinen Institute for Molecular Medicine Finland, HiLIFE, University of Helsinki, Finland
L. Elisa Lahtela Institute for Molecular Medicine Finland, HiLIFE, University of Helsinki, Finland
Matti Peura Institute for Molecular Medicine Finland, HiLIFE, University of Helsinki, Finland
Communication
Mari Kaunisto Institute for Molecular Medicine Finland, HiLIFE, University of Helsinki, Finland
Data Management and IT Infrastructure
Elina Kilpeläinen Institute for Molecular Medicine Finland, HiLIFE, University of Helsinki, Finland
Timo P. Sipilä Institute for Molecular Medicine Finland, HiLIFE, University of Helsinki, Finland
Georg Brein Institute for Molecular Medicine Finland, HiLIFE, University of Helsinki, Finland
Oluwaseun A. Dada Institute for Molecular Medicine Finland, HiLIFE, University of Helsinki, Finland
Awaisa Ghazal Institute for Molecular Medicine Finland, HiLIFE, University of Helsinki, Finland
Anastasia Shcherban Institute for Molecular Medicine Finland, HiLIFE, University of Helsinki, Finland
Genotyping
Kati Donner Institute for Molecular Medicine Finland, HiLIFE, University of Helsinki, Finland
Timo P. Sipilä Institute for Molecular Medicine Finland, HiLIFE, University of Helsinki, Finland
Sample Collection Coordination
Anu Loukola Helsinki Biobank / Helsinki University and Hospital District of Helsinki and Uusimaa, Helsinki

Sample Logistics

Päivi Laiho	THL Biobank / The National Institute of Health and Welfare Helsinki, Finland
Tuuli Sistonen	THL Biobank / The National Institute of Health and Welfare Helsinki, Finland
Essi Kaiharju	THL Biobank / The National Institute of Health and Welfare Helsinki, Finland
Markku Laukkanen	THL Biobank / The National Institute of Health and Welfare Helsinki, Finland
Elina Järvensivu	THL Biobank / The National Institute of Health and Welfare Helsinki, Finland
Sini Lähteenmäki	THL Biobank / The National Institute of Health and Welfare Helsinki, Finland
Lotta Männikkö	THL Biobank / The National Institute of Health and Welfare Helsinki, Finland
Regis Wong	THL Biobank / The National Institute of Health and Welfare Helsinki, Finland

Registry Data Operations

Hannele Mattsson	THL Biobank / The National Institute of Health and Welfare Helsinki, Finland
Kati Kristiansson	THL Biobank / The National Institute of Health and Welfare Helsinki, Finland
Susanna Lemmelä	Institute for Molecular Medicine Finland, HiLIFE, University of Helsinki, Finland
Sami Koskelainen	THL Biobank / The National Institute of Health and Welfare Helsinki, Finland
Tero Hiekkalinna	THL Biobank / The National Institute of Health and Welfare Helsinki, Finland
Teemu Paajanen	THL Biobank / The National Institute of Health and Welfare Helsinki, Finland

Sequencing Informatics

Priit Palta	Institute for Molecular Medicine Finland, HiLIFE, University of Helsinki, Finland
Kalle Pärn	Institute for Molecular Medicine Finland, HiLIFE, University of Helsinki, Finland
Shuang Luo	Institute for Molecular Medicine Finland, HiLIFE, University of Helsinki, Finland
Vishal Sinha	Institute for Molecular Medicine Finland, HiLIFE, University of Helsinki, Finland

Trajectory Team

Tarja Laitinen	Pirkanmaa Hospital District, Tampere, Finland
Harri Siirtola	University of Tampere, Tampere, Finland
Javier Gracia-Tabuenca	University of Tampere, Tampere, Finland
Mika Helminen	University of Tampere, Tampere, Finland
Tiina Luukkaala	University of Tampere, Tampere, Finland
Iida Vähätalo	University of Tampere, Tampere, Finland

Data protection officer

Tero Jyrhämä	Institute for Molecular Medicine Finland, HiLIFE, University of Helsinki, Finland

FinBB - Finnish biobank cooperative

Marco Hautalahti
Laura Mustaniemi
Mirkka Koivusalo
Sarah Smith
Tom Southerington

Biogen Biobank Team contributors

Steering team: Ellen Tsai, Christopher D. Whelan, Paola Bronson, David Sexton, Sally John, Heiko Runz.
Data management team: Eric Marshall, Mehool Patel, Saranya Duraisamy, Timothy Swan.
Extended scientific team: Dennis Baird, Chia-Yen Chen, Susan Eaton, Jake Gagnon, Feng Gao, Cynthia Gubbels, Yunfeng Huang, Varant Kupelian, Kejie Li, Dawei Liu, Stephanie Loomis, Helen McLaughlin, Adele Mitchell, Benjamin Sun.

Research & Development, Biogen Inc., Cambridge, MA, US

PITX2 Function Study Group contributors

Amelia Aránega-Jiménez
Department of Experimental Biology, Faculty of Experimental Sciences, University of Jaen, Jaen, Spain.

Mohamed Chahine
CERVO Brain Research Center and Department of Medicine, Faculty of Medicine, Université Laval, Quebec City, QC, Canada.

Philippe Chevalier
Université Claude Bernard Lyon 1, EA4612 Neurocardiology, Lyon, France.

Georges Christé
Université Claude Bernard Lyon 1, EA4612 Neurocardiology, Lyon, France.

Tim Footz
Department of Medical Genetics, University of Alberta, Edmonton, Alberta, Canada.

Francisco Hernández-Torres
Department of Experimental Biology, Faculty of Experimental Sciences, University of Jaen, Jaen, Spain.

Asma Mechakra
Université Claude Bernard Lyon 1, EA4612 Neurocardiology, Lyon, France.

Gilles Millat
Université Claude Bernard Lyon 1, EA4612 Neurocardiology, Lyon, France.

Elodie Morel
Université Claude Bernard Lyon 1, EA4612 Neurocardiology, Lyon, France.

Michael Walter
Department of Medical Genetics, University of Alberta, Edmonton, Alberta, Canada.

Yi-Qing Yang
Department of Cardiovascular Research, Shanghai Chest Hospital, Shanghai Jiaotong University, Shanghai 200030, China.

Supplementary Information References

51 Sun, B. B. *et al.* Genomic atlas of the human plasma proteome. *Nature* **558**, 73-79, doi:10.1038/s41586-018-0175-2 (2018).

52 Forberg, E., Huhmann, I., Jimenez-Boj, E. & Watzke, H. H. The impact of Glu102Lys on the factor X function in a patient with a doubly homozygous factor X deficiency (Gla14Lys and Glu102Lys). *Thromb Haemost* **83**, 234-238 (2000).

53 Suhre, K. *et al.* Connecting genetic risk to disease end points through the human blood plasma proteome. *Nat Commun* **8**, 14357-14357, doi:10.1038/ncomms14357 (2017).

54 Kujovich, J. L. Factor V Leiden thrombophilia. *Genetics in Medicine* **13**, 1-16, doi:10.1097/GIM.0b013e3181faa0f2 (2011).

55 de Vries, P. S. *et al.* A meta-analysis of 120 246 individuals identifies 18 new loci for fibrinogen concentration. *Hum Mol Genet* **25**, 358-370, doi:10.1093/hmg/ddv454 (2016).

56 Wassel, C. L. *et al.* Association of genomic loci from a cardiovascular gene SNP array with fibrinogen levels in European Americans and African-Americans from six cohort studies: the Candidate Gene Association Resource (CARe). *Blood* **117**, 268-275, doi:10.1182/blood-2010-06-289546 (2011).

57 Simurda, T. *et al.* Genetic Variants in the FGB and FGG Genes Mapping in the Beta and Gamma Nodules of the Fibrinogen Molecule in Congenital Quantitative Fibrinogen Disorders Associated with a Thrombotic Phenotype. *Int J Mol Sci* **21**, doi:10.3390/ijms21134616 (2020).

58 Võsa, U. *et al.* Unraveling the polygenic architecture of complex traits using blood eQTL metaanalysis. *bioRxiv*, 447367, doi:10.1101/447367 (2018).

59 Girotra, M. *et al.* The Current Understanding of the Endocrine Effects From Immune Checkpoint Inhibitors and Recommendations for Management. *JNCI Cancer Spectr* **2**, pky021, doi:10.1093/jncics/pky021 (2018).

60 Diogo, D. *et al.* Phenome-wide association studies across large population cohorts support drug target validation. *Nat Commun* **9**, 4285, doi:10.1038/s41467-018-06540-3 (2018).

61 Buniello, A. *et al.* The NHGRI-EBI GWAS Catalog of published genome-wide association studies, targeted arrays and summary statistics 2019. *Nucleic acids research* **47**, D1005-D1012, doi:10.1093/nar/gky1120 (2019).

62 Petkowski, J. J. *et al.* NRMT2 is an N-terminal monomethylase that primes for its homologue NRMT1. *Biochem J* **456**, 453-462, doi:10.1042/BJ20131163 (2013).

63 Dong, C. *et al.* An asparagine/glycine switch governs product specificity of human N-terminal methyltransferase NTMT2. *Commun Biol* **1**, 183, doi:10.1038/s42003-018-0196-2 (2018).

64 Consortium, G. T. The Genotype-Tissue Expression (GTEx) project. *Nat Genet* **45**, 580-585, doi:10.1038/ng.2653 (2013).

65 Uhlen, M. *et al.* Proteomics. Tissue-based map of the human proteome. *Science* **347**, 1260419, doi:10.1126/science.1260419 (2015).

66 Feghaly, J., Zakka, P., London, B., MacRae, C. A. & Refaat, M. M. Genetics of Atrial Fibrillation. *J Am Heart Assoc* **7**, e009884, doi:10.1161/JAHA.118.009884 (2018).

67 Jabbari, J. *et al.* Common and rare variants in SCN10A modulate the risk of atrial fibrillation. *Circ Cardiovasc Genet* **8**, 64-73, doi:10.1161/HCG.0000000000000022 (2015).
68 Chambers, J. C. *et al.* Genetic variation in SCN10A influences cardiac conduction. *Nat Genet* **42**, 149-152, doi:10.1038/ng.516 (2010).
69 Duan, G. *et al.* A SCN10A SNP biases human pain sensitivity. *Mol Pain* **12**, doi:10.1177/1744806916666083 (2016).
70 Poulet, C. *et al.* Late Sodium Current in Human Atrial Cardiomyocytes from Patients in Sinus Rhythm and Atrial Fibrillation. *PLoS One* **10**, e0131432, doi:10.1371/journal.pone.0131432 (2015).
71 Qi, B. *et al.* Nav1.8 channels in ganglionated plexi modulate atrial fibrillation inducibility. *Cardiovasc Res* **102**, 480-486, doi:10.1093/cvr/cvu005 (2014).
72 Odening, K. E. The Role of Nav1.8 in Cardiac Electrophysiology-a Matter of the Heart or the Nerve? *Cardiovasc Drugs Ther* **33**, 645-647, doi:10.1007/s10557-019-06931-8 (2019).
73 Coates, M. D. *et al.* Impact of the NaV1.8 variant, A1073V, on post-sigmoidectomy pain and electrophysiological function in rat sympathetic neurons. *J Neurophysiol* **122**, 2591-2600, doi:10.1152/jn.00542.2019 (2019).
74 Benson, D. W. *et al.* Congenital sick sinus syndrome caused by recessive mutations in the cardiac sodium channel gene (SCN5A). *J Clin Invest* **112**, 1019-1028, doi:10.1172/JCI18062 (2003).
75 Gui, J. *et al.* Multiple loss-of-function mechanisms contribute to SCN5A-related familial sick sinus syndrome. *PLoS One* **5**, e10985, doi:10.1371/journal.pone.0010985 (2010).
76 Li, W. *et al.* SCN5A Variants: Association With Cardiac Disorders. *Front Physiol* **9**, 1372, doi:10.3389/fphys.2018.01372 (2018).
77 Zaklyazminskaya, E. & Dzemeshkevich, S. The role of mutations in the SCN5A gene in cardiomyopathies. *Biochim Biophys Acta* **1863**, 1799-1805, doi:10.1016/j.bbamcr.2016.02.014 (2016).
78 Olson, T. M. *et al.* Sodium channel mutations and susceptibility to heart failure and atrial fibrillation. *JAMA* **293**, 447-454, doi:10.1001/jama.293.4.447 (2005).
79 Moreau, A., Gosselin-Badaroudine, P. & Chahine, M. Gating pore currents, a new pathological mechanism underlying cardiac arrhythmias associated with dilated cardiomyopathy. *Channels (Austin)* **9**, 139-144, doi:10.1080/19336950.2015.1031937 (2015).
80 Moreau, A. & Chahine, M. A New Cardiac Channelopathy: From Clinical Phenotypes to Molecular Mechanisms Associated With Nav1.5 Gating Pores. *Front Cardiovasc Med* **5**, 139, doi:10.3389/fcvm.2018.00139 (2018).
81 Verkerk, A. O. & Wilders, R. Pacemaker activity of the human sinoatrial node: effects of HCN4 mutations on the hyperpolarization-activated current. *Europace* **16**, 384-395, doi:10.1093/europace/eut348 (2014).
82 Mechakra, A. *et al.* A Novel PITX2c Gain-of-Function Mutation, p.Met207Val, in Patients With Familial Atrial Fibrillation. *Am J Cardiol* **123**, 787-793, doi:10.1016/j.amjcard.2018.11.047 (2019).
83 Strungaru, M. H. *et al.* PITX2 is involved in stress response in cultured human trabecular meshwork cells through regulation of SLC13A3. *Invest Ophthalmol Vis Sci* **52**, 7625-7633, doi:10.1167/iovs.10-6967 (2011).

84 Kozlowski, K. & Walter, M. A. Variation in residual PITX2 activity underlies the phenotypic spectrum of anterior segment developmental disorders. *Hum Mol Genet* **9**, 2131-2139, doi:10.1093/hmg/9.14.2131 (2000).

85 Claycomb, W. C. *et al.* HL-1 cells: a cardiac muscle cell line that contracts and retains phenotypic characteristics of the adult cardiomyocyte. *Proc Natl Acad Sci U S A* **95**, 2979-2984, doi:10.1073/pnas.95.6.2979 (1998).

86 Saleem, R. A., Banerjee-Basu, S., Murphy, T. C., Baxevanis, A. & Walter, M. A. Essential structural and functional determinants within the forkhead domain of FOXC1. *Nucleic Acids Res* **32**, 4182-4193, doi:10.1093/nar/gkh742 (2004).

87 Galli, D. *et al.* Atrial myocardium derives from the posterior region of the second heart field, which acquires left-right identity as Pitx2c is expressed. *Development* **135**, 1157-1167, doi:10.1242/dev.014563 (2008).

88 Simard, A. *et al.* The Pitx2c N-terminal domain is a critical interaction domain required for asymmetric morphogenesis. *Dev Dyn* **238**, 2459-2470, doi:10.1002/dvdy.22062 (2009).

89 Perez-Hernandez, M. *et al.* Pitx2c increases in atrial myocytes from chronic atrial fibrillation patients enhancing IKs and decreasing ICa,L. *Cardiovasc Res* **109**, 431-441, doi:10.1093/cvr/cvv280 (2016).

90 Nattel, S. New ideas about atrial fibrillation 50 years on. *Nature* **415**, 219-226, doi:10.1038/415219a (2002).

91 Hong, K., Bjerregaard, P., Gussak, I. & Brugada, R. Short QT syndrome and atrial fibrillation caused by mutation in KCNH2. *J Cardiovasc Electrophysiol* **16**, 394-396, doi:10.1046/j.1540-8167.2005.40621.x (2005).

92 Ghouse, J. *et al.* Rare genetic variants previously associated with congenital forms of long QT syndrome have little or no effect on the QT interval. *Eur Heart J* **36**, 2523-2529, doi:10.1093/eurheartj/ehv297 (2015).

93 Wetzel, U. *et al.* Expression of connexins 40 and 43 in human left atrium in atrial fibrillation of different aetiologies. *Heart* **91**, 166-170, doi:10.1136/hrt.2003.024216 (2005).
