# Supplementary material for: Genetic associations of protein-coding variants in human disease
Source: Nature. 2022 Feb 23;603(7899):95–102. doi: 10.1038/s41586-022-04394-w (PMC8891017; doi:10.1038/s41586-022-04394-w)
Supplement: Supplementary file 2 — Reporting Summary [file 41586_2022_4394_MOESM2_ESM.pdf]

nature research

Corresponding author(s): Benjamin Sun and Heiko Runz

Last updated by author(s): Nov 3, 2021

Reporting Summary

Nature Research wishes to improve the reproducibility of the work that we publish. This form provides structure for consistency and transparency in reporting. For further information on Nature Research policies, see our Editorial Policies and the Editorial Policy Checklist.

Statistics

For all statistical analyses, confirm that the following items are present in the figure legend, table legend, main text, or Methods section.

n/a	Confirmed	
☐	☒	The exact sample size (*n*) for each experimental group/condition, given as a discrete number and unit of measurement
☐	☒	A statement on whether measurements were taken from distinct samples or whether the same sample was measured repeatedly
☐	☒	The statistical test(s) used AND whether they are one- or two-sided<br>*Only common tests should be described solely by name; describe more complex techniques in the Methods section.*
☐	☒	A description of all covariates tested
☐	☒	A description of any assumptions or corrections, such as tests of normality and adjustment for multiple comparisons
☐	☒	A full description of the statistical parameters including central tendency (e.g. means) or other basic estimates (e.g. regression coefficient) AND variation (e.g. standard deviation) or associated estimates of uncertainty (e.g. confidence intervals)
☐	☒	For null hypothesis testing, the test statistic (e.g. *F*, *t*, *r*) with confidence intervals, effect sizes, degrees of freedom and *P* value noted<br>*Give P values as exact values whenever suitable.*
☒	☐	For Bayesian analysis, information on the choice of priors and Markov chain Monte Carlo settings
☒	☐	For hierarchical and complex designs, identification of the appropriate level for tests and full reporting of outcomes
☐	☒	Estimates of effect sizes (e.g. Cohen's *d*, Pearson's *r*), indicating how they were calculated

Our web collection on statistics for biologists contains articles on many of the points above.

Software and code

Policy information about availability of computer code

Data collection: No specific software were employed in collection

Data analysis:
R v3.6.1 including packages: PheWAS v0.99.5, icd v4.0.9, MRClust (custom), RSpectra v0.16, GenomicRanges v1.38, ComplexHeatmap v2.7.11
REGENIE v1.0.6.7
SAIGE v0.39
METAL v2011-03-2
PLINK v1.9 and v2.0
AxiomGT1
Eagle v2.3.5
SISu v3
Hail v0.1
VEP v103
Details of specific software and references, including genetic measurements and QC, can be found within text in the relevant Methods and Supplementary Information sections.

Code availability statement included.
Code availability
Custom analysis scripts used are available at https://github.com/cnfoley/Sun-et-al-2021-protein-coding-variants-in-human-disease.

For manuscripts utilizing custom algorithms or software that are central to the research but not yet described in published literature, software must be made available to editors and reviewers. We strongly encourage code deposition in a community repository (e.g. GitHub). See the Nature Research guidelines for submitting code & software for further information.

Data

Policy information about availability of data

All manuscripts must include a data availability statement. This statement should provide the following information, where applicable:

- Accession codes, unique identifiers, or web links for publicly available datasets
- A list of figures that have associated raw data
- A description of any restrictions on data availability

Data availability statement present and updated.
Data availability
Full summary association results of this study are accessible at https://doi.org/10.5281/zenodo.5571000. Summary and individual-level whole exome sequencing data from UKB participants have been deposited with UKB and will be freely available to approved researchers via The UK Biobank Research Analysis Platform (https://www.ukbiobank.ac.uk/enable-your-research/research-analysis-platform). FG summary association results are being released bi-annually via https://www.finngen.fi/en/access_results and can be explored in a public results browser (https://r5.finngen.fi). Individual-level genotypes from FinnGen participants can be accessed by approved researchers via the Fingenious portal (https://site.fingenious.fi/en/) hosted by the Finnish Biobank Cooperative FinBB (https://finbb.fi/en/). Further datasets underlying this study have been derived from: Therapeutic Target Database (http://db.idrblab.net/ttd/); Phecode-ICD10 data (https://phewascatalog.org/phecodes_icd10); GWAS Catalog (https://www.ebi.ac.uk/gwas/); PhenoScanner (http://www.phenoscanner.medschl.cam.ac.uk/); ClinVar (https://www.ncbi.nlm.nih.gov/clinvar/); gnomAD (https://gnomad.broadinstitute.org/); Human Protein Atlas (https://www.proteinatlas.org/); and Ensembl (https://www.ensembl.org/index.html).

Field-specific reporting

Please select the one below that is the best fit for your research. If you are not sure, read the appropriate sections before making your selection.

☒ Life sciences ☐ Behavioural & social sciences ☐ Ecological, evolutionary & environmental sciences

For a reference copy of the document with all sections, see nature.com/documents/nr-reporting-summary-flat.pdf

Life sciences study design

All studies must disclose on these points even when the disclosure is negative.

Sample size	Methods, "Samples and participants"; Introduction sections. Note: this is a discovery study across multiple phenotypes and thus we did not perform specific power calculations to detect pre-specified effect sizes - but we are well-powered as our study combines two of the largest available population cohorts than previous studies. Sample size were chosen from the largest data available to maximise power, since this is a discovery study, the goal is to maximise power rather than to detect at specific effect sizes.
Data exclusions	Methods, "Genetic data processing", "Disease endpoint association analyses", Extended Data Figure 1. Samples failing QC and non-overlapping phenotypes were excluded as per methods.
Replication	Methods, "Definition and refinement of significant regions". In addition to multiple testing correction, we also required replication at p<0.05 in UKB/FG with concordant direction of effects. With in results, we also include cases where replication associations in previous GWAS. Replication/all summary data are available online.
Randomization	No experimental vs control group per se.
Blinding	No experimental vs control group per se. All data are anonymised and analysts were blind to sample statuses.

Reporting for specific materials, systems and methods

We require information from authors about some types of materials, experimental systems and methods used in many studies. Here, indicate whether each material, system or method listed is relevant to your study. If you are not sure if a list item applies to your research, read the appropriate section before selecting a response.

Materials & experimental systems

n/a	Involved in the study	
☐	☒	Antibodies
☐	☒	Eukaryotic cell lines
☒	☐	Palaeontology and archaeology
☒	☐	Animals and other organisms
☐	☒	Human research participants
☒	☐	Clinical data
☒	☐	Dual use research of concern

Methods

n/a	Involved in the study	
☒	☐	ChIP-seq
☒	☐	Flow cytometry
☒	☐	MRI-based neuroimaging

Antibodies

Antibodies used	For Western blots on HL-1 cell protein extracts, antibodies were a mouse monoclonal anti-V5 (V8012, Sigma-Aldrich) and a mouse monoclonal anti-GAPDH (G8795, Sigma-Aldrich). Batch numbers unknown.<br><br>Antibodies used with TM-1 cells experiments:<br>Anti-Xpress Monoclonal Antibody was purchased from ThermoFisher Scientific (#R910-25). Batch number unknown.<br><br>Goat Anti-Mouse-IgG coupled to Cyanine3: Cy™3 AffiniPure Goat Anti-Mouse IgG (H+L) polyclonal, was purchased from Jackson ImmunoResearch Laboratories Inc. (#115-165-003). Batch number unknown. (115-165-003).
Validation	V8012, Sigma-Aldrich<br>https://www.sigmaaldrich.com/FR/fr/search/v8012?<br>focus=products&page=1&perPage=30&sort=relevance&term=V8012&type=product<br>the website says: V8012 V5-10, monoclonal WB, ICC<br>https://www.sigmaaldrich.com/FR/fr/search/v8012?focus=products&page=1&perPage=30&s<br>ort=relevance&term=V8012&type=product<br>the website says: V8012 V5-10, monoclonal WB, ICC<br>G8795, Sigma-Aldrich<br>https://www.sigmaaldrich.com/FR/fr/search/g8795?<br>focus=products&page=1&perPage=30&sort=relevance&term=G8795&type=product<br>the website says: G8795 GAPDH-71.1, monoclonal WB, ARR, ICC, ELISA mouse, mink, rabbit, rat, human, hamster, canine, turkey, chicken, monkey, bovine.<br>Sigma-Aldrich quote papers and show images from the literature in which Abs V8012 and G8795 were used, but do not endorse them as a validation.<br>R910-25, Thermofisher<br>https://www.thermofisher.com/antibody/product/Xpress-Antibody-Monoclonal/R910-25.<br>The website says: "R910-25 is tested in Western blot against 100 ng of an E. coli expressed fusion protein containing the Xpress epitope."<br>115-165-003, Jackson Immunoresearch<br>https://www.jacksonimmuno.com/catalog/products/115-165-003<br>the website does not provide validation.

Eukaryotic cell lines

Policy information about cell lines

Cell line source(s)	The TM-1 cell line (immortalized human Trabecular Meshwork cells) was a gift from Dr. Vincent Raymond, and was cultured in Dulbecco's Modified Eagle's Medium - low glucose (Sigma, #D6046), see Filla et. al., 2002, IOVS. 43:151; PMID: 11773026. HL-1 cells were a gift from Dr. W.C. Claycomb (doi: 10.1073/pnas.95.6.2979) and were cultured using his dedicated "Claycomb Medium" ordered from Sigma-Aldrich (product 51800C) and the Fetal Calf Serum lot certified by Dr. Claycomb, ordered from Sigma-Aldrich (product F2442 lot #058K8426). Since Dr. W.C. Claycomb passed-away the HL-1 cells are provided by SIGMA and other firms.
Authentication	TM-1 cells are not authenticated.<br>HL-1 cells were authenticated by Dr. W.C. Claycomb.<br>No authentication tests have been performed in our laboratories.
Mycoplasma contamination	No cell line was tested for mycoplasma contamination in our laboratories.
Commonly misidentified lines<br>(See ICLAC register)	The ISLAC register searches for TM-1 or HTM or HTMC and for HL-1 did not report any match.

Human research participants

Policy information about studies involving human research participants

Population characteristics	Details can be found in Supplementary table 1 which contains UKB and FG population characteristics. Also described in Introduction and in Methods "Samples and participants" sections. UKB and FG have been commonly described in the public domain - they are two population cohorts from the UK and Finland respectively.
Recruitment	UKB and FG are population cohorts sampled from across UK sites and Finnish biobanks respectively. Detailed in Methods ("Samples and participants") and Supplementary Information.
Ethics oversight	Methods ("Samples and participants") and Supplementary Information for approval and ethics details. In detail:<br>Analyses in this study were conducted under UK Biobank Approved Project number 26041. UK Biobank has approval from the North West Multi-centre Research Ethics Committee (MREC), which covers the UK. It also sought the approval in England and Wales from the Patient Information Advisory Group (PIAG) for gaining access to information that would allow it to invite people to participate. PIAG has since been replaced by the National Information Governance Board for Health & Social Care (NIGB). In Scotland, UK Biobank has approval from the Community Health Index Advisory Group (CHIAG). UK Biobank possesses a Human Tissue Authority (HTA) licence, so a separate HTA licence is not required by researchers who receive samples from the resource, so long as residual samples are destroyed or returned at the end of the research project, and applicants do not transfer the samples to third party premises without the specific approval of UK Biobank. UK Biobank has

sought generic Research Tissue Bank (RTB) approval, which should cover the large majority of research using the resource. This approach is recommended by the National Research Ethics Service and UK Biobank governing Research Ethics Committee (REC), which approved the application in 2010. Researchers should check the UK Biobank Access Procedures for more detail. FinnGen ethics statement details Patients and control subjects in FinnGen provided informed consent for biobank research, based on the Finnish Biobank Act. Alternatively, separate research cohorts, collected prior the Finnish Biobank Act came into effect (in September 2013) and start of FinnGen (August 2017), were collected based on study-specific consents and later transferred to the Finnish biobanks after approval by Fimea, the National Supervisory Authority for Welfare and Health. Recruitment protocols followed the biobank protocols approved by Fimea. The Coordinating Ethics Committee of the Hospital District of Helsinki and Uusimaa (HUS) approved the FinnGen study protocol Nr HUS/990/2017. The FinnGen study is approved by Finnish Institute for Health and Welfare (permit numbers: THL/2031/6.02.00/2017, THL/1101/5.05.00/2017, THL/341/6.02.00/2018, THL/2222/6.02.00/2018, THL/283/6.02.00/2019, THL/1721/5.05.00/2019, THL/1524/5.05.00/2020, and THL/2364/14.02/2020), Digital and population data service agency (permit numbers: VRK43431/2017-3, VRK/6909/2018-3, VRK/4415/2019-3), the Social Insurance Institution (permit numbers: KELA 58/522/2017, KELA 131/522/2018, KELA 70/522/2019, KELA 98/522/2019, KELA 138/522/2019, KELA 2/522/2020, KELA 16/522/2020 and Statistics Finland (permit numbers: TK-53-1041-17 and TK-53-90-20). The Biobank Access Decisions for FinnGen samples and data utilized in FinnGen Data Freeze 6 include: THL Biobank BB2017_55, BB2017_111, BB2018_19, BB_2018_34, BB_2018_67, BB2018_71, BB2019_7, BB2019_8, BB2019_26, BB2020_1, Finnish Red Cross Blood Service Biobank 7.12.2017, Helsinki Biobank HUS/359/2017, Auria Biobank AB17-5154, Biobank Borealis of Northern Finland_2017_1013, Biobank of Eastern Finland 1186/2018, Finnish Clinical Biobank Tampere MH0004, Central Finland Biobank 1-2017, and Terveystalo Biobank STB 2018001.

Note that full information on the approval of the study protocol must also be provided in the manuscript.
